# Proteolytic remodelling of the extracellular matrix by pericytes

**DOI:** 10.1111/febs.70569

**Published:** 2026-05-07

**Authors:** Tina Burkhard, Ella Milne, Kate Qian, Paola Campagnolo, Salvatore Santamaria

**Affiliations:** ^1^ Discipline of Clinical Sciences, School of Biosciences University of Surrey UK; ^2^ Department of Comparative Biomedical Sciences School of Veterinary Medicine Surrey UK

**Keywords:** basement membrane, extracellular matrix, matrisome, pericytes, proteases

## Abstract

Pericytes (PCs) are perivascular cells that lie in close association with endothelial cells (ECs), with both cell types embedded within a shared basement membrane (BM), a specialised form of extracellular matrix (ECM). PCs regulate vascular integrity, angiogenesis and capillary blood flow and are capable of differentiating into other cell types including fibroblasts and smooth muscle cells. In recent years, a central role for PCs in regulating the development and maturation of the vasculature, maintaining tissue homeostasis and directing the pleiotropic remodelling of tissues during regeneration has emerged. Here, we review how PCs contribute to the synthesis and remodelling of the ECM in different pathophysiological conditions. Moreover, we provide an atlas of the PC matrisome, the complex of ECM molecules expressed by PCs, based on recent transcriptomics (in particular single‐cell RNA sequencing) and proteomics datasets, with the caveat that such an entity does not exist in isolation due to the physical and paracrine interactions between PCs, ECs and other cell types. Understanding the role of PCs in modulating their microenvironment through active synthesis and degradation of specific matrisome components is essential to understand the role these plastic cells play in angiogenesis and in different pathologic conditions, including stroke, Alzheimer's disease and cancer.

## Graphical abstract

Pericytes are specialised perivascular cells intimately connected with endothelial cells and essential for the maintenance of vascular beds. They contribute to the formation and remodelling of the extracellular matrix by actively secreting proteases and protease inhibitors. Under pathologic conditions, this delicate proteolytic balance is altered, leading to profound changes that compromise vascular integrity and function.

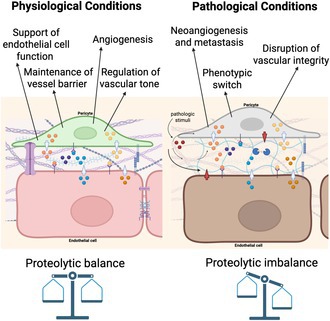

**Abbreviations**
AC: astrocytes
AD: Alzheimer's disease
ADAM: A Disintegrin‐like metalloproteinases
ADAMTS: A Disintegrin‐Like and Metalloproteinase with Thrombospondin motifs
AGE: glycation end‐products
ANGPT: angiopoietin
BBB: blood–brain barrier
BM: basement membrane
BMP1: bone morphogenetic protein 1
BRB: blood‐retinal barrier
CCL: chemokine (C‐C motif) ligand
CCM: cerebral cavernous malformation
CKD: chronic kidney disease
CNS: central nervous system
CSPG4: chondroitin sulfate proteoglycan‐4
CXCL: chemokine (C‐X‐C motif) ligand
DR: diabetic retinopathy
EC: endothelial cells
ECM: extracellular matrix
EGF: epidermal growth factor
FGF: fibroblast growth factor
FSTL1: follistatin like 1
GAG: glycosaminoglycan
GM‐IVH: germinal matrix‐intraventricular haemorrhage
HGF: hepatocyte growth factor
HSCs: hepatic stellate cells
HTRA1: high temperature requirement A1
IL: interleukin
IM: interstitial matrix
LOX: lysyl oxidase
LOXL: LOX‐like
LTBP: latent TGF‐β binding protein
MFAP‐2: microfibrillar‐associated protein 2
MMP: matrix metalloproteases
NG2: Neuron‐glial antigen 2
PAI‐1: Plasminogen Activator Inhibitor 1
PC: pericytes
PDGF: platelet‐derived growth factor
PDGFR: PDGF receptor
scRNA‐seq: single‐cell RNA sequencing
SLRP: small leucine‐rich proteoglycan
TGF: transforming growth factor
TIMP: tissue inhibitor of metalloproteinase
TNF: tumour necrosis factor
tPA: tissue plasminogen activator
uPA: urokinase‐type plasminogen activator
VEGF: vascular endothelial growth factor

## Introduction

**Fig. 1:**
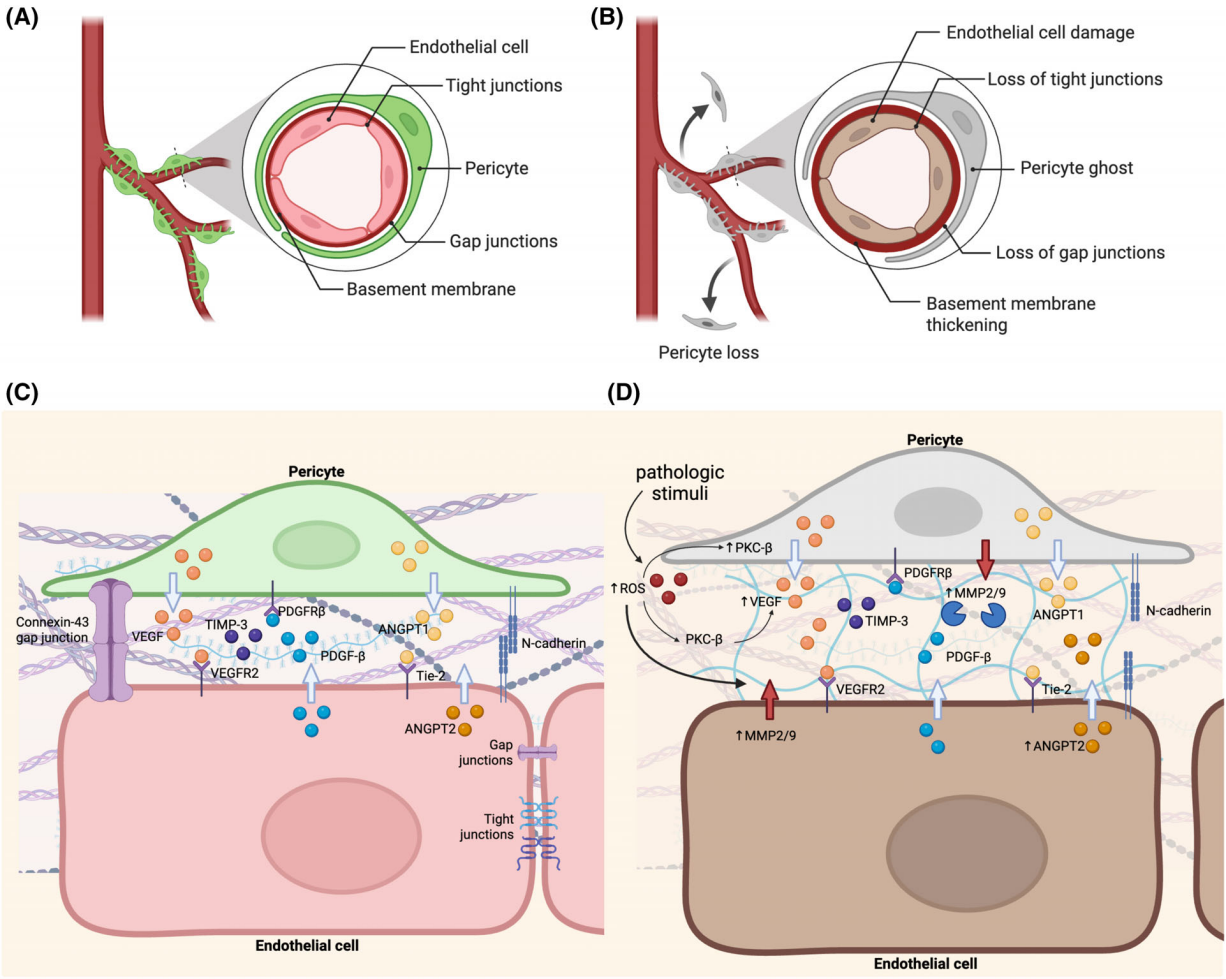
Schematic representation of the interactions between pericytes and endothelial cells. Physiological (A) and pathological (B) cross‐section of a capillary. (C, D) Molecular interactions between endothelial cells (ECs) and pericytes (PCs) under physiological (C) and pathological (D) conditions. Gap junctions formed by Connexin‐43 and N‐cadherins mediate PC‐EC interactions. ECs secrete paracrine factors such as PDGF‐β and ANGPT2. PDGF‐β mediates PC recruitment through PDGFRβ on PCs. PCs secrete VEGF and ANGPT1, which act as pro‐ and anti‐angiogenic factors, respectively, by binding to VEGFR and Tie‐2 on ECs. In the presence of pathological stimuli, for example oxidative stress, interactions between ECs and PCs are disrupted and the protein kinase C (PKC) signalling pathway is upregulated, thus stimulating VEGF expression and subsequent angiogenesis. There is an increased secretion of gelatinases MMP2 and MMP9 which, because of the altered MMP/TIMP‐3 ratio, cause excessive degradation of basement membrane (BM) components such as collagen IV and gap/tight junctions. The macroscopic effects summarised in (B) involve loss of gap junctions leading to PC detachment (leaving behind a so‐called ‘pericyte ghost’), and BM thickening caused by increased expression of specific extracellular matrix molecules such as fibronectin (not shown). ANGPT1, angiopoietin‐1; ANGPT2, angiopoietin‐2; MMP, matrix metalloproteinase; PDGF‐β, platelet‐derived growth factor β; PDGFRβ, platelet‐derived growth factor receptor β; ROS, reactive oxygen species; TIMP‐3, tissue inhibitor of metalloproteinase 3; VEGF, vascular endothelial growth factor; VEGFR, vascular endothelial growth factor receptor. Figure created with BioRender.

Pericytes (PCs) are perivascular cells of mesenchymal nature, intimately connected to endothelial cells (ECs) of the small vessels (i.e. the capillaries) by long branches protruding from their soma [1]. While the soma contains the nucleus and most of the cytoplasm, the processes are enriched in cytoskeletal proteins. Direct interactions between ECs and PCs are established through so‐called peg‐and‐socket structures, as well as adhesion plaques and gap junctions [2] (Fig. 1).

PCs are ubiquitous in the body, and their frequency is related to the vasculature density and characteristics, ranging from 1:1 ECs:PCs in the kidney and retina, up to 1:100 in the skeletal muscle [3, 4]. Regardless of their phenotypic definition (*see* Section ‘The pericyte matrisome: an Atlas’), PC roles include but are not limited to: (1) supporting EC function, (2) regulating vascular tone, (3) promoting angiogenesis (the formation of new blood vessels from existing ones), vessel maturation and wound healing; (4) maintaining vessel barrier, therefore tightly regulating the exchange between blood and organs/tissues [5, 6, 7].

The interactions between PCs and ECs are mediated by soluble paracrine signals, reciprocal physical interactions through gap and tight junctions, cell surface adhesion molecules and the extracellular matrix (ECM), the network of secreted macromolecules surrounding both cell types. In particular, both PCs and ECs are surrounded by the basement membrane (BM), a thin, sheet‐like ECM structure forming a physical barrier whose integrity is crucial to vascular function (Fig. 1).

The BM plays an important role in capillary mechanical stability, which in large vessels is granted by the smooth muscle layer and mediates PC regulation of blood flow by transmitting contraction and relaxation [8]. The blood–brain barrier (BBB), a specialised form of capillary interface that crucially regulates molecule exchange between blood and the brain tissue, is particularly reliant on an intact BM produced by ECs, PCs and astrocytes (ACs) [9].

**Fig. 2:**
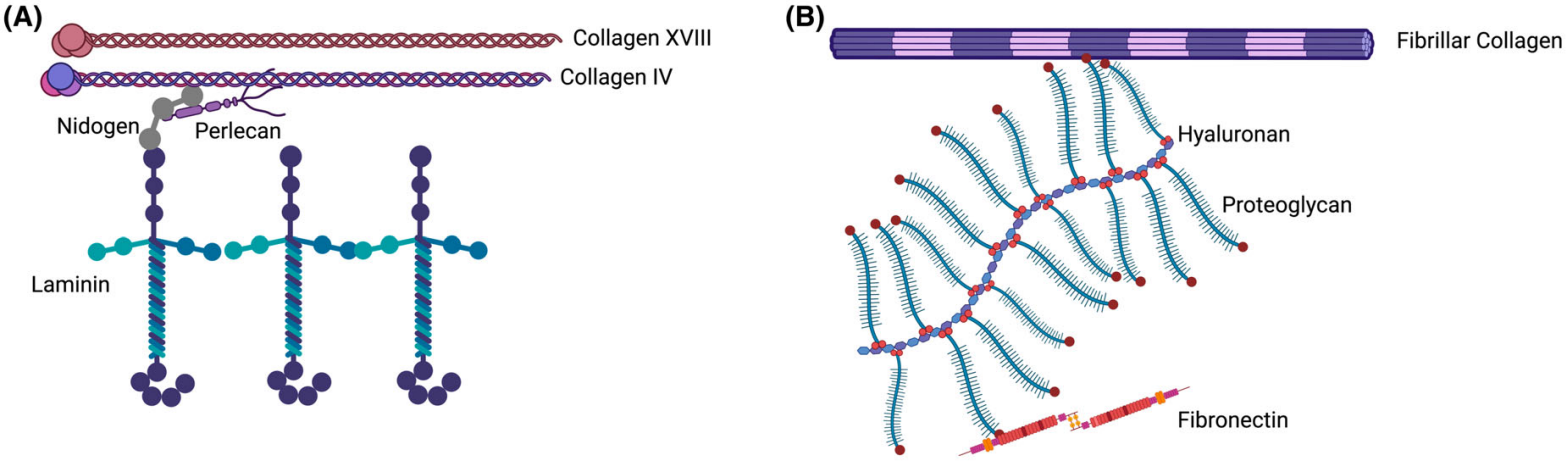
Major components of the basement membrane and interstitial matrix. The basement membrane (A) is a network consisting of collagen type IV, laminins, nidogens, heparan sulfate proteoglycans like perlecan and the hybrid collagen/proteoglycan collagen type XVIII. The interstitial matrix (B) consists instead of fibrillar collagens (type I, II and III), fibronectin and chondroitin sulfate proteoglycans such as aggrecan and versican bound to the unsulfated glycosaminoglycan hyaluronan. Figure created with BioRender.

PCs are crucial for BM deposition, although both PCs and ECs contribute to this process [10, 11]. The core components of the BM include collagens (in particular type IV), laminins, nidogens/entactins, heparan sulfate proteoglycans (in particular agrin and perlecan) and the hybrid collagen/proteoglycan collagen type XVIII [12, 13]. PCs contribute to the dynamicity of BM by releasing proteases (including matrix metalloproteases, MMPs), ECM‐modifying enzymes, protease inhibitors, and other ECM regulators, as well as cytokines, chemokines and growth factors through paracrine signalling and the release of extracellular vesicles [9] (Fig. 1). Excessive proteolytic activity leads to breach of the BM, a process crucial in angiogenesis and pathological processes, including metastasis [14]. Together with fibroblasts located in the surrounding interstitial tissue, PCs also contribute to the deposition and remodelling of the interstitial matrix (IM), a gel‐like scaffold consisting of fibrillar collagens (type I, II and III), fibronectin and proteoglycans [15] (Fig. 2). The IM surrounds capillaries in the tissues, fills gaps between cells and connects to the BM.

PCs are a heterogenous population displaying a range of specific markers, differentiation abilities and ECM production capacity depending on the vasculature type (precapillary, capillary, postcapillary) and organ association, reflecting their varied embryonic origin, even within the same organ. Commonly accepted PC markers include platelet‐derived growth factor (PDGF) receptor β (PDGFRβ), neuron‐glial antigen 2 (NG2), α‐smooth muscle actin, alkaline phosphatase, desmin, aminopeptidases A and N, G‐protein signalling 5, melanoma cell adhesion molecule (CD146) and nestin [4, 16]. A combination of these markers and/or a morphological description are often required for unequivocal identification of PCs [16]. PCs are embryonically diverse as they originate from organ‐specific mesenchymal cells, therefore their specific expression pattern varies in different tissues and anatomic districts [17]. For instance, PCs in the central nervous system (CNS), such as those in the retina and brain, at least partially derive from the neural crest, while those in other organs such as lung, liver, gut and heart are of mesodermal origin [18, 19]. Additionally, expression of PC markers may be altered under pathological conditions. PCs can undergo phenotypic switching to myofibroblasts, especially in fibrosis [20, 21], and respond to a variety of stimuli such as hypoxia, high glucose levels, oxidative stress, shear stress and lipopolysaccharide [22]. The PC matrisome is therefore very plastic.

Recent advancements in multimodal single‐cell analysis such as single‐cell RNA sequencing (scRNA‐seq) and linage tracing have enabled a progressive refinement of the PC profile, identifying organ‐specific markers such as Notch3 in heart and kidney, and phospholipase A1 member A and Cytochrome c oxidase subunit 4 isoform 2 in the brain [23]. Profiling PC subpopulations spatially, and especially in disease, is an active field of research [24, 25].

Here, we highlight how PCs regulate the composition and properties of the perivascular ECM in health and disease, with a particular focus on the BM. We will start by providing an atlas of the ECM proteins secreted by PCs, that is, the PC matrisome. We will then highlight ECM remodelling by PCs in health and disease and provide examples of ECM/PC crosstalk for specific organs. As we focus on one specific aspect of PC biology, that is, their role in proteolytic remodelling of the ECM, we will refer to more comprehensive reviews for further reading.

## The pericyte matrisome: An atlas

**Fig. 3:**
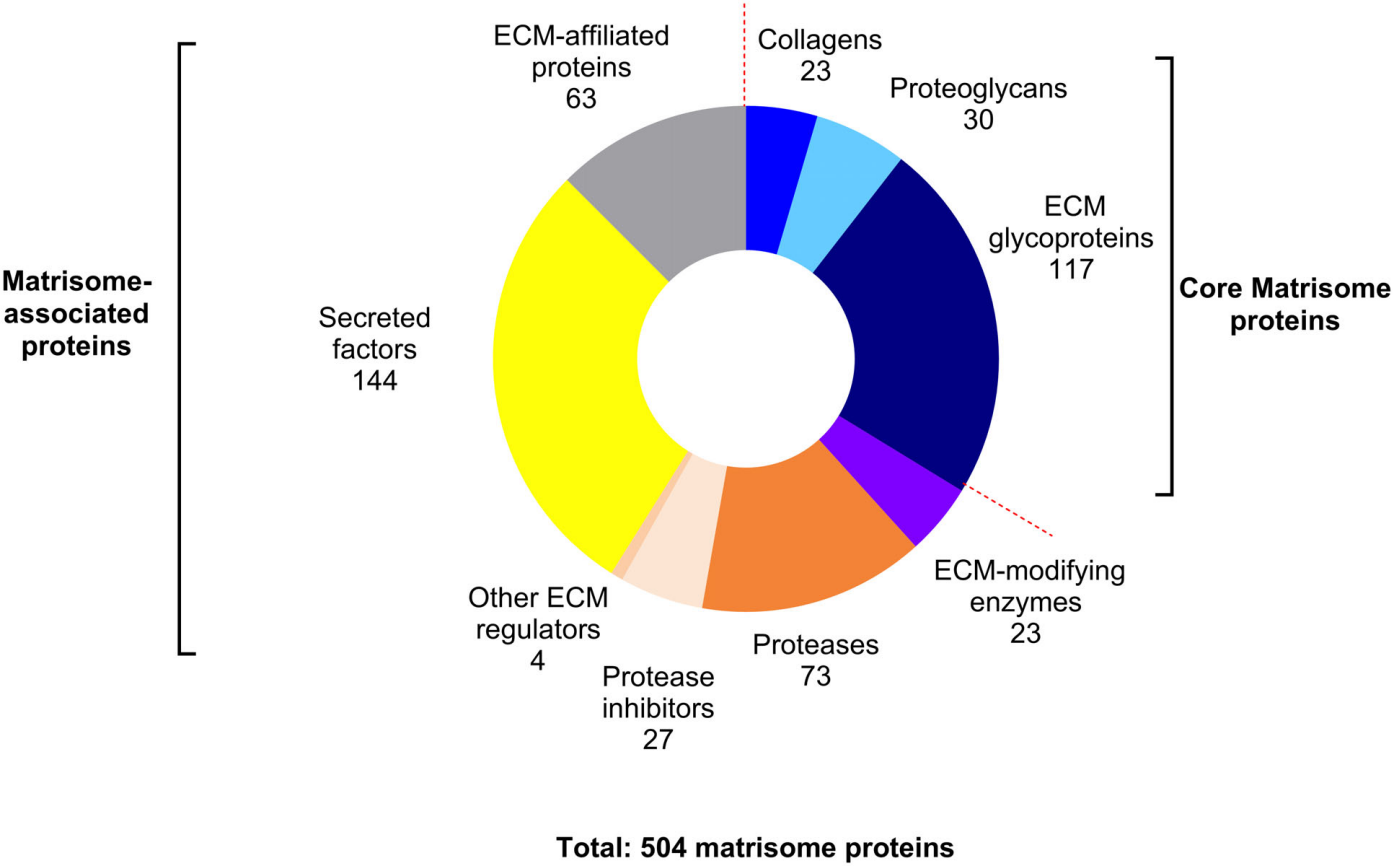
Composition of the pericyte matrisome. The donut plot shows how the 504 matrisome proteins specific to pericytes are classified according to the major categories listed by Naba *et al*. [31]. ECM, extracellular matrix. Figure created with GraphPad Prism.

In this section, we will highlight common features of the PC matrisome, while in the following sections we will describe the changes that this undergoes during development and in pathological states. Table 1 provides a comprehensive list of matrisome components, which are schematically outlined in Fig. 3.

**Table 1:** Components of the pericyte matrisome.

Category	Matrisome component	Detection method	Source	References
*Core matrisome*				
Collagens	Collagen I	IHC	Adult mouse kidney PCs	[3]
		IHC	Human placental PCs	[49]
		Immunoblot	Human placental PCs	[48]
		Proteomics	Human brain PCs	[27, 29]
		RT‐PCR	Adult mouse cardiac PCs	[46]
		RT‐PCR	Mouse/rat HSCs	[267]
		RT‐PCR	Adult rat HSCs	[263]
		scRNA‐seq	Adult mouse pancreatic PCs	[39]
		scRNA‐seq	Adult mouse cardiac PCs	[40]
		scRNA‐seq	Early postnatal mouse brain PCs	[50]
		scRNA‐seq	Infant mouse brain PCs	[44]
		scRNA‐seq	Human brain PCs	[42, 43]
		scRNA‐seq	Mouse post‐natal retinal PCs	[51]
		scRNA‐seq	Embryonic mouse brain PCs	[45]
		scRNA‐seq	Human cardiac PCs	[52]
		scRNA‐seq	PCs in vascular organoids from non‐diabetic donors	[30]
	Collagen III	Proteomics	Human placental PCs	[48]
		Proteomics	Human brain PCs	[27]
		RT‐PCR	Adult rat HSCs	[263]
		scRNA‐seq	Adult mouse pancreatic PCs	[39]
		scRNA‐seq	Early postnatal mouse brain PCs	[50]
		scRNA‐seq	Infant mouse brain PCs	[44]
		scRNA‐seq	Adult mouse cardiac PCs	[40]
		scRNA‐seq	Human brain PCs	[42]
		scRNA‐seq	Embryonic brain PCs	[45]
		scRNA‐seq	PCs in vascular organoids from non‐diabetic donors	[30]
	Collagen IV	IHC	Human placental PCs	[49]
		Immunoblot	Human brain PCs	[38]
		Proteomics	Human placental PCs	[48]
		RT‐PCR	Adult mouse cardiac PCs	[46]
		RT‐PCR	Adult rat HSCs	[263]
		RT‐PCR	Human retinal PCs	[47]
		scRNA‐seq	Adult mouse pancreatic PCs	[39]
		scRNA‐seq	Adult mouse cardiac PCs	[40]
		scRNA‐seq	Adult mouse brain PCs	[41]
		scRNA‐seq	Human brain PCs	[42, 43]
		scRNA‐seq	Infant mouse brain PCs	[44]
		scRNA‐seq	Embryonic brain PCs	[45]
		scRNA‐seq	Adult mouse cardiac PCs	[46]
		scRNA‐seq	PCs in vascular organoids from diabetic and non‐diabetic donors	[30]
	Collagen V	Proteomics	Human placental PCs	[48]
		Proteomics	Human brain PCs	[27]
		scRNA‐seq	Adult mouse pancreatic PCs	[39]
		scRNA‐seq	Human brain PCs	[42, 43]
		scRNA‐seq	Infant mouse brain PCs	[44]
		scRNA‐seq	Adult mouse cardiac PCs	[46]
		scRNA‐seq	Human cardiac PCs	[52]
		scRNA‐seq	Adult mouse cardiac PCs	[40]
		scRNA‐seq	PCs in vascular organoids from non‐diabetic donors	[30]
	Collagen VI	Proteomics	Human placental PCs	[48]
		scRNA‐seq	Adult mouse pancreatic PCs	[39]
		scRNA‐seq	Early postnatal mouse brain PCs	[50]
		scRNA‐seq	Human brain PCs	[42]
		scRNA‐seq	Adult mouse brain PCs	[41]
		scRNA‐seq	Infant mouse brain PCs	[44]
		scRNA‐seq	Adult mouse cardiac PCs	[40, 46]
		scRNA‐seq	PCs in vascular organoids from non‐diabetic donors	[30]
	Collagen VII	scRNA‐seq	Human brain PCs	[42]
	Collagen VIII	Proteomics	Human placental PCs	[48]
		scRNA‐seq	Human DCM cardiac PCs	[52]
	Collagen IX	scRNA‐seq	Human brain PCs	[42]
	Collagen XI	scRNA‐seq	Human brain PCs	[42]
		scRNA‐seq	PCs in vascular organoids from non‐diabetic donors	[30]
	Collagen XII	Proteomics	Human placental PCs	[48]
		scRNA‐seq	PCs in vascular organoids from non‐diabetic donors	[30]
	Collagen XIII	FACS	Adult mouse cardiac PCs	[40]
		scRNA‐seq	Infant mouse brain PCs	[44]
	Collagen XIV	scRNA‐seq	Human brain PCs	[42]
		scRNA‐seq	Human DCM cardiac PCs	[52]
	Collagen XVI	scRNA‐seq	Human brain PCs	[42]
		scRNA‐seq	PCs in vascular organoids from non‐diabetic donors	[30]
	Collagen XVII	scRNA‐seq	Adult mouse brain PCs	[41]
	Collagen XIX	scRNA‐seq	Human brain PCs	[42]
	Collagen XXI	scRNA‐seq	Human brain PCs	[42]
	Collagen XXII	scRNA‐seq	Human brain PCs	[42]
		scRNA‐seq	Human DCM cardiac PCs	[52]
	Collagen XXIII	scRNA‐seq	PCs in vascular organoids from diabetic donors	[30]
	Collagen XXIV	scRNA‐seq	Human brain PCs	[42]
		scRNA‐seq	Human DCM cardiac PCs	[52]
	Collagen XXV	scRNA‐seq	Human DCM cardiac PCs	[52]
	Collagen XXVI	scRNA‐seq	PCs in vascular organoids from diabetic donors	[30]
	Collagen XXVII	scRNA‐seq	Human brain PCs	[42]
		scRNA‐seq	Human cardiac PCs	[52]
	Collagen XXVIII	scRNA‐seq	Human brain PCs	[42]
Proteoglycans	Aggrecan	scRNA‐seq	Human DCM cardiac PCs	[52]
		scRNA‐seq	PCs in vascular organoids from non‐diabetic donors	[30]
	ASPN	scRNA‐seq	Early postnatal mouse brain PCs	[50]
		scRNA‐seq	Embryonic brain PCs	[45]
		scRNA‐seq	Human brain PCs	[42]
	Biglycan	Proteomics	Human placental PCs	[48]
		scRNA‐seq	Adult mouse pancreatic PCs	[39]
		scRNA‐seq	Adult human brain PCs	[42, 43]
		scRNA‐seq	Embryonic brain PCs	[45]
		scRNA‐seq	Human cardiac PCs	[52]
	Brevican	scRNA‐seq	Human brain PCs	[42]
		scRNA‐seq	PCs in vascular organoids from non‐diabetic donors	[30]
	Chondroadherin	scRNA‐seq	Human brain PCs	[42]
	Collagen XV	scRNA‐seq	Adult mouse pancreatic PCs	[39]
		scRNA‐seq	Infant mouse brain PCs	[44]
	Collagen XVIII	scRNA‐seq	Adult mouse pancreatic PCs	[39]
		scRNA‐seq	Adult human pancreatic PCs	[76]
		scRNA‐seq	Adult mouse brain PCs	[41]
		scRNA‐seq	Adult human brain PCs	[42, 43]
		scRNA‐seq	Human cardiac PCs	[52]
		scRNA‐seq	PCs in vascular organoids from non‐diabetic donors	[30]
	CSPG4/NG2	IHC	Adult mouse heart	[66]
		IHC	Adult mouse pancreatic PCs	[39]
		scRNA‐seq	Early post‐natal mouse brain PCs	[50]
		scRNA‐seq	Mouse post‐natal retinal PCs	[51]
		scRNA‐seq	Adult human pancreatic PCs	[76]
		scRNA‐seq	Embryonic mouse brain PCs	[45]
		scRNA‐seq	PCs in vascular organoids from non‐diabetic donors	[30]
	Decorin	scRNA‐seq	Adult mouse pancreatic PCs	[39]
		scRNA‐seq	Adult human brain PCs	[42, 43]
		scRNA‐seq	Human cardiac PCs	[52]
	ESM1	scRNA‐seq	Adult human pancreatic PCs	[76]
	Fibromodulin	Proteomics	Adult mouse heart	[66]
	GPC1	scRNA‐seq	Human cardiac PCs	[52]
	GPC3	scRNA‐seq	Adult mouse brain PCs	[41]
		scRNA‐seq	PCs in vascular organoids from diabetic donors	[30]
	GPC4	scRNA‐seq	Adult mouse brain PCs	[41]
	GPC5	scRNA‐seq	Adult human brain PCs	[42, 43]
	GPC6	scRNA‐seq	Adult mouse brain PCs	[41]
		scRNA‐seq	PCs in vascular organoids from non‐diabetic donors	[30]
	HSPG2/perlecan	Proteomics	Human placental PCs	[48]
		RT‐PCR	Human retinal PCs	[47]
		scRNA‐seq	Infant mouse brain PCs	[44]
		scRNA‐seq	Adult mouse pancreatic PCs	[39]
		scRNA‐seq	PCs in vascular organoids from non‐diabetic donors	[30]
	IMPG1	scRNA‐seq	Human DCM cardiac PCs	[52]
	IMPG2	scRNA‐seq	Human brain PCs	[42]
	Lumican	scRNA‐seq	Adult mouse pancreatic PCs	[39]
		scRNA‐seq	Human brain PCs	[42]
		scRNA‐seq	PCs in vascular organoids from diabetic donors	[30]
	Osteoglycin	IHC/proteomics	Adult mouse heart	[66]
		scRNA‐seq	Adult mouse pancreatic PCs	[39]
		scRNA‐seq	Adult mouse brain PCs	[41]
		scRNA‐seq	Human brain PCs	[42]
	Podocan	Proteomics	Human placental PCs	[48]
		scRNA‐seq	Adult mouse pancreatic PCs	[39]
		scRNA‐seq	Human brain PCs	[42]
	PODNL1	scRNA‐seq	Human brain PCs	[42]
	PRELP	scRNA‐seq	Adult mouse brain PCs	[41]
		scRNA‐seq	Adult human brain PCs	[43]
	PRG4	scRNA‐seq	Human brain PCs	[42]
	SDC1	scRNA‐seq	PCs in vascular organoids from diabetic donors	[30]
	SDC2	Proteomics	Adult mouse heart	[66]
		Proteomics	Human brain PCs	[27]
		scRNA‐seq	Early postnatal mouse brain PCs	[50]
		scRNA‐seq	PCs in vascular organoids from non‐diabetic donors	[30]
	SDC3	scRNA‐seq	Adult mouse brain PCs	[41]
		scRNA‐seq	Human brain PCs	[42]
	SDC4	scRNA‐seq	Adult mouse brain PCs	[41]
	Versican	Proteomics	Human placental PCs	[48]
		scRNA‐seq	PCs in vascular organoids from non‐diabetic donors	[30]
*ECM glycoproteins*	AEBP1	scRNA‐seq	Human DCM cardiac PCs	[52]
		scRNA‐seq	PCs in vascular organoids from diabetic donors	[30]
	Agrin	RT‐PCR	Human retinal PCs	[47]
		Proteomics	Human brain PCs	[27]
		scRNA‐seq	Adult mouse pancreatic PCs	[39]
		scRNA‐seq	Adult mouse brain PCs	[41]
		scRNA‐seq	Human DCM cardiac PCs	[52]
		scRNA‐seq	Adult human brain PCs	[43]
		scRNA‐seq	PCs in vascular organoids from non‐diabetic donors	[30]
	BMPER	scRNA‐seq	PCs in vascular organoids from diabetic donors	[30]
	CILP1	scRNA‐seq	Human brain PCs	[42]
	CILP2	scRNA‐seq	Human brain PCs	[42]
		scRNA‐seq	Human DCM cardiac PCs	[52]
	COMP	scRNA‐seq	Human DCM cardiac PCs	[52]
	CRELD1	scRNA‐seq	PCs in vascular organoids from diabetic donors	[30]
	CRELD2	scRNA‐seq	Human cardiac PCs	[52]
	CRIM1	scRNA‐seq	Adult mouse brain PCs	[41]
		scRNA‐seq	Human DCM cardiac PCs	[52]
		scRNA‐seq	PCs in vascular organoids from non‐diabetic donors	[30]
	CRISPLD1	scRNA‐seq	Human brain PCs	[42]
	CRISPLD2	scRNA‐seq	Adult mouse brain PCs	[41]
		scRNA‐seq	Adult human brain PCs	[43]
		scRNA‐seq	Human cardiac PCs	[52]
	CTF1	scRNA‐seq	Human brain PCs	[42]
		scRNA‐seq	PCs in vascular organoids from non‐diabetic donors	[30]
	CTGF	RT‐PCR	Adult mouse cardiac PCs	[46]
		Proteomics	Human placental PCs	[48]
		scRNA‐seq	Adult mouse brain PCs	[41]
		scRNA‐seq	Human DCM cardiac PCs	[52]
		scRNA‐seq	Adult mouse cardiac PCs	[40]
		scRNA‐seq	PCs in vascular organoids from non‐diabetic donors	[30]
	CTHRC1	scRNA‐seq	Human brain PCs	[42]
	DMBT1	scRNA‐seq	Human brain PCs	[42]
	ECM1	Proteomics	Murine PC secretome	[28]
		scRNA‐seq	Mouse adult HSCs	[162]
		scRNA‐seq	Adult mouse brain PCs	[41]
		scRNA‐seq	Infant mouse brain PCs	[44]
	ECM2	scRNA‐seq	Early postnatal mouse brain PCs	[50]
		scRNA‐seq	Adult mouse brain PCs	[41]
		scRNA‐seq	Human brain PCs	[42]
		scRNA‐seq	PCs in vascular organoids from non‐diabetic donors	[30]
	EDIL3	scRNA‐seq	Adult human brain PCs	[43]
	EFEMP2	scRNA‐seq	PCs in vascular organoids from non‐diabetic donors	[30]
	EGFLAM	scRNA‐seq	Adult mouse brain PCs	[41]
		scRNA‐seq	Human brain PCs	[42]
	Elastin	Immunoblot/proteomics	Human placental PCs	[48]
		RT‐PCR	Adult rat HSCs	[263]
		scRNA‐seq	Adult mouse pancreatic PCs	[39]
	EMID1	scRNA‐seq	PCs in vascular organoids from diabetic donors	[30]
	EMILIN1	scRNA‐seq	Human brain PCs	[42]
		scRNA‐seq	Human cardiac PCs	[52]
		scRNA‐seq	PCs in vascular organoids from non‐diabetic donors	[30]
	FBN1	Proteomics	Human placental PCs	[48]
		scRNA‐seq	Human DCM cardiac PCs	[52]
		scRNA‐seq	PCs in vascular organoids from non‐diabetic donors	[30]
	FBN2	Proteomics	Human placental PCs	[48]
		scRNA‐seq	PCs in vascular organoids from diabetic donors	[30]
	FBN3	Proteomics	Human placental PCs	[48]
	FBLN1	Proteomics	Human placental PCs	[48]
		scRNA‐seq	Adult mouse brain PCs	[41]
		scRNA‐seq	Human cardiac PCs	[52]
		scRNA‐seq	PCs in vascular organoids from diabetic donors	[30]
	FBLN2	Proteomics	Human placental PCs	[48]
		scRNA‐seq	Human brain PCs	[42]
		scRNA‐seq	Infant mouse brain PCs	[44]
	FBLN5	ELISA/RT‐PCR	Adult mouse HSCs	[265]
		scRNA‐seq	Human brain PCs	[42]
		scRNA‐seq	PCs in vascular organoids from non‐diabetic donors	[30]
	Fibrinogen α	Proteomics	Murine PC secretome	[28]
	Fibrinogen β	Proteomics	Murine PC secretome	[28]
	Fibrinogen γ	Proteomics	Murine PC secretome	[28]
	FN1	FACS	Human foetal brain PCs	[83]
		IHC	Human placental PCs	[49]
		Immunoblot	Human brain PCs	[38]
		Immunoblot/proteomics	Human placental PCs	[48]
		Proteomics	Murine PC secretome	[28]
		scRNA‐seq	Human brain PCs	[42]
		scRNA‐seq	Infant mouse brain PCs	[44]
		scRNA‐seq	Adult mouse cardiac PCs	[40]
		scRNA‐seq	PCs in vascular organoids from non‐diabetic donors	[30]
	FRAS1	scRNA‐seq	Human DCM cardiac PCs	[52]
	GAS6	scRNA‐seq	Adult mouse brain PCs	[41]
		scRNA‐seq	PCs in vascular organoids from non‐diabetic donors	[30]
	HAPLN1	scRNA‐seq	Human DCM cardiac PCs	[52]
		scRNA‐seq	PCs in vascular organoids from diabetic donors	[30]
	HAPLN2	scRNA‐seq	Human cardiac PCs	[52]
	HAPLN4	scRNA‐seq	Human brain PCs	[42]
	HMCN1	scRNA‐seq	Human brain PCs	[42]
		scRNA‐seq	Human DCM cardiac PCs	[52]
	IGFBP2	Proteomics	Murine PC secretome	[28]
		scRNA‐seq	PCs in vascular organoids from diabetic donors	[30]
	IGFBP3	scRNA‐seq	Human brain PCs	[42]
		scRNA‐seq	PCs in vascular organoids from diabetic donors	[30]
	IGFBP4	Proteomics	Murine PC secretome	[28]
		scRNA‐seq	Human brain PCs	[42]
		scRNA‐seq	Human cardiac PCs	[52]
	IGFPB5	scRNA‐seq	Human brain PCs	[42]
		scRNA‐seq	Human cardiac PCs	[52]
	IGFBP6	scRNA‐seq	Human cardiac PCs	[52]
		scRNA‐seq	PCs in vascular organoids from diabetic donors	[30]
	IGFBP7	scRNA‐seq	Human brain PCs	[42]
		scRNA‐seq	Embryonic mouse brain PCs	[45]
		scRNA‐seq	PCs in vascular organoids from non‐diabetic donors	[30]
	LAMA1	scRNA‐seq	PCs in vascular organoids from non‐diabetic donors	[30]
	LAMA2	scRNA‐seq	Adult mouse pancreatic PCs	[39]
		scRNA‐seq	Early postnatal mouse brain PCs	[50]
		scRNA‐seq	Adult mouse brain PCs	[41]
		scRNA‐seq	Mouse post‐natal retinal PCs	[51]
		scRNA‐seq	Human brain PCs	[42, 43]
	LAMA3	scRNA‐seq	Human DCM cardiac PCs	[52]
	LAMA4	scRNA‐seq	Adult mouse pancreatic PCs	[39]
		scRNA‐seq	Adult human pancreatic PCs	[76]
		scRNA‐seq	Adult mouse brain PCs	[41]
		scRNA‐seq	Human brain PCs	[42]
		scRNA‐seq	Embryonic mouse brain PCs	[45]
		scRNA‐seq	PCs in vascular organoids from diabetic donors	[30]
	LAMA5	Proteomics	Human placental PCs	[48]
		scRNA‐seq	Human brain PCs	[42]
		scRNA‐seq	Infant mouse brain PCs	[44]
		scRNA‐seq	Human DCM cardiac PCs	[52]
		scRNA‐seq	PCs in vascular organoids from non‐diabetic donors	[30]
	LAMB1	Proteomics	Human placental PCs	[48]
		RT‐PCR	Human retinal PCs	[47]
		scRNA‐seq	Adult mouse pancreatic PCs	[39]
		scRNA‐seq	Adult mouse brain PCs	[41]
		scRNA‐seq	Human brain PCs	[42]
		scRNA‐seq	Embryonic mouse brain PCs	[45]
		scRNA‐seq	Human cardiac PCs	[52]
		scRNA‐seq	PCs in vascular organoids from non‐diabetic donors	[30]
	LAMB2	scRNA‐seq	Adult mouse pancreatic PCs	[39]
		scRNA‐seq	Adult mouse brain PCs	[41]
		scRNA‐seq	Infant mouse brain PCs	[44]
		scRNA‐seq	Human DCM cardiac PCs	[52]
		scRNA‐seq	PCs in vascular organoids from non‐diabetic donors	[30]
	LAMB3	scRNA‐seq	Human brain PCs	[42]
	LAMB4	scRNA‐seq	Human DCM cardiac PCs	[52]
	LAMC1	Proteomics	Human placental PCs	[48]
		scRNA‐seq	Adult mouse pancreatic PCs	[39]
		scRNA‐seq	Adult mouse brain PCs	[41]
		scRNA‐seq	Embryonic mouse brain PCs	[45]
		scRNA‐seq	PCs in vascular organoids from non‐diabetic donors	[30]
	LAMC3	scRNA‐seq	Early postnatal mouse brain PCs	[50]
		scRNA‐seq	Adult mouse brain PCs	[41]
		scRNA‐seq	Adult human brain PCs	[43]
	LGI2	scRNA‐seq	PCs in vascular organoids from diabetic donors	[30]
	LTBP1	scRNA‐seq	Adult mouse brain PCs	[41]
		scRNA‐seq	Human brain PCs	[42]
		scRNA‐seq	Human DCM cardiac PCs	[52]
	LTBP2	scRNA‐seq	Human brain PCs	[42]
		scRNA‐seq	Human DCM cardiac PCs	[52]
	LTBP3	scRNA‐seq	Human brain PCs	[42]
		scRNA‐seq	PCs in vascular organoids from non‐diabetic donors	[30]
	LTBP4	scRNA‐seq	Adult mouse brain PCs	[41]
		scRNA‐seq	Human brain PCs	[42]
		scRNA‐seq	PCs in vascular organoids from non‐diabetic donors	[30]
	MFAP1	scRNA‐seq	Human cardiac PCs	[52]
		scRNA‐seq	PCs in vascular organoids from non‐diabetic donors	[30]
	MFAP2	scRNA‐seq	Infant mouse brain PCs	[44]
		scRNA‐seq	Adult mouse cardiac PCs	[46]
		scRNA‐seq	PCs in vascular organoids from diabetic donors	[30]
		scRNA‐seq	Adult mouse HSCs	[264]
	MFGE8	scRNA‐seq	Adult human pancreatic PCs	[76]
		scRNA‐seq	Embryonic mouse brain PCs	[45]
		scRNA‐seq	Human cardiac PCs	[52]
		scRNA‐seq	PCs in vascular organoids from non‐diabetic donors	[30]
	MGP	scRNA‐seq	Mouse post‐natal retinal PCs	[51]
		scRNA‐seq	Adult mouse brain PCs	[41]
		scRNA‐seq	Human brain PCs	[42]
		scRNA‐seq	Embryonic mouse brain PCs	[45]
		scRNA‐seq	Human cardiac PCs	[52]
		scRNA‐seq	PCs in vascular organoids from non‐diabetic donors	[30]
	MMRN2	scRNA‐seq	PCs in vascular organoids from non‐diabetic donors	[30]
	MXRA5	scRNA‐seq	Human brain PCs	[42]
		scRNA‐seq	Human DCM cardiac PCs	[52]
		scRNA‐seq	PCs in vascular organoids from non‐diabetic donors	[30]
	Nidogen 1	scRNA‐seq	Adult mouse pancreatic PCs	[39]
		scRNA‐seq	Adult mouse brain PCs	[41]
		scRNA‐seq	Embryonic mouse brain PCs	[45]
		scRNA‐seq	Adult human brain PCs	[43]
		scRNA‐seq	PCs in vascular organoids from non‐diabetic donors	[30]
	Nidogen 2	scRNA‐seq	Adult mouse pancreatic PCs	[39]
		scRNA‐seq	Adult mouse brain PCs	[41]
		scRNA‐seq	Human cardiac PCs	[52]
		scRNA‐seq	PCs in vascular organoids from non‐diabetic donors	[30]
	NPNT	scRNA‐seq	PCs in vascular organoids from non‐diabetic donors	[30]
	NTN1	scRNA‐seq	Early postnatal mouse brain PCs	[50]
		scRNA‐seq	Adult mouse brain PCs	[41]
		scRNA‐seq	Human brain PCs	[42]
	NTN4	scRNA‐seq	Adult human pancreatic PCs	[76]
		scRNA‐seq	Human DCM cardiac PCs	[52]
	NTN5	scRNA‐seq	Human brain PCs	[42]
	NTN G2	scRNA‐seq	Human brain PCs	[42]
	OIT3	scRNA‐seq	Human DCM cardiac PCs	[52]
	OVGP1	scRNA‐seq	Human DCM cardiac PCs	[52]
	papilin	scRNA‐seq	Human brain PCs	[42]
	PCOLCE	scRNA‐seq	Early postnatal mouse brain PCs	[50]
		scRNA‐seq	Human brain PCs	[42]
		scRNA‐seq	Adult mouse cardiac PCs	[46]
		scRNA‐seq	Human cardiac PCs	[52]
		scRNA‐seq	PCs in vascular organoids from non‐diabetic donors	[30]
	periostin	FACS	Adult mouse cardiac PCs	[40]
		Proteomics	Human placental PCs	[48]
		scRNA‐seq	Human brain PCs	[42]
		scRNA‐seq	Adult mouse cardiac PCs	[46]
		scRNA‐seq	PCs in vascular organoids from non‐diabetic donors	[30]
	POMZP3	scRNA‐seq	PCs in vascular organoids from non‐diabetic donors	[30]
	PXDN	scRNA‐seq	Adult mouse brain PCs	[41]
		scRNA‐seq	PCs in vascular organoids from non‐diabetic donors	[30]
	PXDNL	scRNA‐seq	PCs in vascular organoids from diabetic donors	[30]
	Reelin	Proteomics	Murine PC secretome	[28]
		scRNA‐seq	Mouse adult HSCs	[162]
	SLIT1	scRNA‐seq	PCs in vascular organoids from non‐diabetic donors	[30]
	SLIT2	scRNA‐seq	PCs in vascular organoids from non‐diabetic donors	[30]
	SLIT3	scRNA‐seq	Adult human pancreatic PCs	[76]
		scRNA‐seq	PCs in vascular organoids from non‐diabetic donors	[30]
	SMOC1	Proteomics	Adult mouse heart	[66]
	SMOC2	scRNA‐seq	Adult human brain PCs	[43]
	SNED1	scRNA‐seq	PCs in vascular organoids from non‐diabetic donors	[30]
	SPOCK1	scRNA‐seq	Human brain PCs	[42]
	SPOCK2	scRNA‐seq	Mouse post‐natal retinal PCs	[51]
		scRNA‐seq	Adult mouse brain PCs	[41]
		scRNA‐seq	Human brain PCs	[42]
	SPON1	scRNA‐seq	PCs in vascular organoids from diabetic donors	[30]
	SPON2	scRNA‐seq	PCs in vascular organoids from non‐diabetic donors	[30]
	SPARC	Proteomics	Human placental PCs	[48]
		Proteomics	Human PCs	[99]
		scRNA‐seq	Adult mouse brain PCs	[41]
		scRNA‐seq	Adult human brain PCs	[42, 43]
		scRNA‐seq	PCs in vascular organoids from non‐diabetic donors	[30]
	SPARCL1	scRNA‐seq	Adult mouse brain PCs	[41]
		scRNA‐seq	Embryonic mouse brain PCs	[45]
		scRNA‐seq	Human cardiac PCs	[52]
		scRNA‐seq	PCs in vascular organoids from diabetic donors	[30]
	SPP1	RT‐PCR	Human brain PCs	[159]
		scRNA‐seq	Adult mouse brain PCs	[41]
	SVEP1	scRNA‐seq	Human brain PCs	[42]
		scRNA‐seq	Human DCM cardiac PCs	[52]
	Tectorin α	scRNA‐seq	Human brain PCs	[42]
	THBS1	Proteomics	Human placental PCs	[48]
		RT‐PCR	Adult mouse cardiac PCs	[46]
		scRNA‐seq	Infant mouse brain PCs	[44]
		scRNA‐seq	PCs in vascular organoids from non‐diabetic donors	[30]
	THBS2	Proteomics	Human brain PCs	[27]
		RT‐PCR	Adult mouse cardiac PCs	[46]
		scRNA‐seq	Human brain PCs	[42]
	THBS3	scRNA‐seq	Human brain PCs	[42]
	THBS4	scRNA‐seq	Adult human pancreatic PCs	[76]
		scRNA‐seq	Human cardiac PCs	[52]
		scRNA‐seq	PCs in vascular organoids from non‐diabetic donors	[30]
	TINAGL1	scRNA‐seq	Adult human pancreatic PCs	[76]
		scRNA‐seq	Adult mouse brain PCs	[41]
	TNC	FACS	Adult mouse cardiac PCs	[40]
		Proteomics	Human brain PCs	[27, 29]
		Proteomics	Human placental PCs	[48]
		scRNA‐seq	Adult mouse cardiac PCs	[40, 46]
	TNFAIP6	scRNA‐seq	PCs in vascular organoids from non‐diabetic donors	[30]
	TNN	scRNA‐seq	PCs in vascular organoids from non‐diabetic donors	[30]
	TNR	Proteomics	Human brain PCs	[29]
	TNX	scRNA‐seq	Adult mouse pancreatic PCs	[39]
	TNXB	scRNA‐seq	Adult mouse brain PCs	[41]
		scRNA‐seq	Human brain PCs	[42]
	TSKU	scRNA‐seq	PCs in vascular organoids from non‐diabetic donors	[30]
	VTN	IHC, ISH	Infant mouse retinal and brain PCs	[84]
		Proteomics	Human placental PCs	[48]
		Proteomics	Human retinal PCs	[85]
		scRNA‐seq	Early postnatal mouse brain PCs	[50]
		scRNA‐seq	Adult mouse brain PCs	[41]
		scRNA‐seq	Infant mouse brain PCs	[44]
		scRNA‐seq	Embryonic mouse brain PCs	[45]
		Proteomics	Murine PC secretome	[28]
	vWF	Proteomics	Human brain PCs	[29]
		Proteomics	Murine PC secretome	[28]
		scRNA‐seq	Adult human pancreatic PCs	[76]
	vWA5A	scRNA‐seq	PCs in vascular organoids from diabetic donors	[30]
	WISP1	scRNA‐seq	Human brain PCs	[42]
		scRNA‐seq	Human cardiac PCs	[52]
	WISP2	scRNA‐seq	Human brain PCs	[42]
	ZP3	scRNA‐seq	PCs in vascular organoids from non‐diabetic donors	[30]
	ZPLD1	scRNA‐seq	Human DCM cardiac PCs	[52]
*Matrisome associated*				
ECM affiliated				
	ANXA1	scRNA‐seq	Human DCM cardiac PCs	[52]
		scRNA‐seq	PCs in vascular organoids from non‐diabetic donors	[30]
	ANXA2	Proteomics	Human placental PCs	[48]
	ANXA3	scRNA‐seq	Adult mouse brain PCs	[41]
	ANXA4	scRNA‐seq	Human brain PCs	[42]
		scRNA‐seq	Human DCM cardiac PCs	[52]
	ANXA5	scRNA‐seq	Embryonic mouse brain PCs	[45]
		scRNA‐seq	Human cardiac PCs	[52]
	ANXA6	scRNA‐seq	Adult human pancreatic PCs	[76]
		scRNA‐seq	PCs in vascular organoids from non‐diabetic donors	[30]
	ANXA9	scRNA‐seq	Human brain PCs	[42]
	ANXA10	scRNA‐seq	Human DCM cardiac PCs	[52]
	ANXA11	scRNA‐seq	Adult mouse brain PCs	[41]
		scRNA‐seq	PCs in vascular organoids from non‐diabetic donors	[30]
	ANXA8L1	scRNA‐seq	Human cardiac PCs	[52]
	C1QA	scRNA‐seq	Human cardiac PCs	[52]
	C1QB	scRNA‐seq	Human cardiac PCs	[52]
		scRNA‐seq	PCs in vascular organoids from non‐diabetic donors	[30]
	C1QC	scRNA‐seq	Mouse post‐natal retinal PCs	[51]
		scRNA‐seq	Human cardiac PCs	[52]
		scRNA‐seq	PCs in vascular organoids from non‐diabetic donors	[30]
	C1QL1	scRNA‐seq	PCs in vascular organoids from non‐diabetic donors	[30]
	C1QTNF1	scRNA‐seq	Adult human pancreatic PCs	[76]
		scRNA‐seq	Human brain PCs	[42]
		scRNA‐seq	Adult human brain PCs	[43]
	C1QTNF2	scRNA‐seq	Adult mouse brain PCs	[41]
	C1QTNF4	scRNA‐seq	Human brain PCs	[42]
		scRNA‐seq	PCs in vascular organoids from diabetic donors	[30]
	C1QTNF6	scRNA‐seq	Human brain PCs	[42]
	C1QTNF7	scRNA‐seq	Adult mouse brain PCs	[41]
	CLEC2D	scRNA‐seq	Human DCM cardiac PCs	[52]
	CLEC3B	scRNA‐seq	Human brain PCs	[42]
	CLEC11A	scRNA‐seq	Human brain PCs	[42]
		scRNA‐seq	PCs in vascular organoids from diabetic donors	[30]
	CLEC18A	scRNA‐seq	Human brain PCs	[42]
	CLEC18B	scRNA‐seq	Human brain PCs	[42]
	COLEC10	scRNA‐seq	PCs in vascular organoids from diabetic donors	[30]
	COLEC11	scRNA‐seq	Mouse adult HSCs	[162]
		scRNA‐seq	PCs in vascular organoids from non‐diabetic donors	[30]
	COLEC12	scRNA‐seq	Adult mouse brain PCs	[41]
		scRNA‐seq	Human brain PCs	[42]
		scRNA‐seq	Adult human brain PCs	[43]
	ELFN1	scRNA‐seq	PCs in vascular organoids from non‐diabetic donors	[30]
	ELFN2	scRNA‐seq	Human brain PCs	[42]
	EMCN	scRNA‐seq	Adult human pancreatic PCs	[76]
		scRNA‐seq	Adult mouse brain PCs	[41]
		scRNA‐seq	PCs in vascular organoids from diabetic donors	[30]
	FCN3	scRNA‐seq	Adult human pancreatic PCs	[76]
	Hemopexin	Proteomics	Murine PC secretome	[28]
		scRNA‐seq	Human brain PCs	[42]
	LGALS1	scRNA‐seq	Human cardiac PCs	[52]
	LGALS2	scRNA‐seq	Human brain PCs	[42]
	LGALS3	scRNA‐seq	Human brain PCs	[42]
		scRNA‐seq	Human cardiac PCs	[52]
		scRNA‐seq	PCs in vascular organoids from non‐diabetic donors	[30]
	LGALS9	scRNA‐seq	Adult mouse brain PCs	[41]
	LMAN1	Proteomics	Human placental PCs	[48]
		scRNA‐seq	Adult mouse brain PCs	[41]
		scRNA‐seq	Human cardiac PCs	[52]
	Mucin 1	scRNA‐seq	Human brain PCs	[42]
	Mucin 3A	scRNA‐seq	Human DCM cardiac PCs	[52]
	Mucin 6	scRNA‐seq	Human DCM cardiac PCs	[52]
	Mucin 20	scRNA‐seq	Human brain PCs	[42]
		scRNA‐seq	PCs in vascular organoids from diabetic donors	[30]
	PLXDC1	scRNA‐seq	Early postnatal mouse brain PCs	[50]
		scRNA‐seq	Adult human brain PCs	[42, 43]
		scRNA‐seq	Adult mouse brain PCs	[41]
		scRNA‐seq	PCs in vascular organoids from non‐diabetic donors	[30]
	PLXDC2	scRNA‐seq	Early postnatal mouse brain PCs	[50]
		scRNA‐seq	Adult human brain PCs	[42, 43]
		scRNA‐seq	Adult mouse brain PCs	[41]
		scRNA‐seq	Human DCM cardiac PCs	[52]
	PLXNA1	scRNA‐seq	Human DCM cardiac PCs	[52]
		scRNA‐seq	PCs in vascular organoids from non‐diabetic donors	[30]
	PLXNA3	scRNA‐seq	Human DCM cardiac PCs	[52]
		scRNA‐seq	PCs in vascular organoids from non‐diabetic donors	[30]
	PLXNA4	scRNA‐seq	Human brain PCs	[42]
	PLXNB1	RT‐PCR	Adult retinal PCs	[104]
		scRNA‐seq	Human brain PCs	[42]
		scRNA‐seq	Human retinal PCs	[103]
	PLXNB2	scRNA‐seq	Human brain PCs	[42]
		scRNA‐seq	Human DCM cardiac PCs	[52]
		scRNA‐seq	PCs in vascular organoids from non‐diabetic donors	[30]
	PLXNC1	scRNA‐seq	Human brain PCs	[42]
	PLXND1	scRNA‐seq	Human cardiac PCs	[52]
	SEMA3B	scRNA‐seq	Human brain PCs	[42]
	SEMA3D	scRNA‐seq	Human brain PCs	[42]
	SEMA3E	scRNA‐seq	Human brain PCs	[42]
	SEMA3F	scRNA‐seq	Human DCM cardiac PCs	[52]
	SEMA4A	scRNA‐seq	Human brain PCs	[42]
	SEMA4B	scRNA‐seq	Human brain PCs	[42]
		scRNA‐seq	PCs in vascular organoids from non‐diabetic donors	[30]
	SEMA4C	scRNA‐seq	PCs in vascular organoids from non‐diabetic donors	[30]
	SEMA4G	scRNA‐seq	Human brain PCs	[42]
	SEMA5A	scRNA‐seq	Early postnatal mouse brain PCs	[50]
		scRNA‐seq	Adult human brain PCs	[43]
		scRNA‐seq	PCs in vascular organoids from diabetic donors	[30]
	SEMA5B	scRNA‐seq	Human brain PCs	[42]
		scRNA‐seq	Human DCM cardiac PCs	[52]
		scRNA‐seq	PCs in vascular organoids from non‐diabetic donors	[30]
	SEMA6A	scRNA‐seq	Human brain PCs	[42]
	SEMA6C	scRNA‐seq	Adult mouse brain PCs	[41]
		scRNA‐seq	Human brain PCs	[42]
	SEMA6D	scRNA‐seq	Human DCM cardiac PCs	[52]
		scRNA‐seq	Adult mouse brain PCs	[41]
		scRNA‐seq	PCs in vascular organoids from non‐diabetic donors	[30]
ECM regulators				
ECM‐modifying enzymes	EGLN1	scRNA‐seq	PCs in vascular organoids from non‐diabetic donors	[30]
	EGLN3	scRNA‐seq	Human cardiac PCs	[52]
	FAM20A	scRNA‐seq	PCs in vascular organoids from diabetic donors	[30]
	FAM20B	scRNA‐seq	Human DCM cardiac PCs	[52]
	FAM20C	scRNA‐seq	PCs in vascular organoids from non‐diabetic donors	[30]
	Heparinase 2	scRNA‐seq	PCs in vascular organoids from non‐diabetic donors	[30]
	HYAL2	scRNA‐seq	PCs in vascular organoids from diabetic donors	[30]
	LOX	RT‐PCR	Adult rat HSCs	[263]
		scRNA‐seq	Adult mouse cardiac PCs	[46]
		scRNA‐seq	PCs in vascular organoids from non‐diabetic donors	[30]
	LOXL1	Proteomics	Murine PC secretome	[28]
		RT‐PCR	Adult rat HSCs	[263]
	LOXL2	RT‐PCR	Adult rat HSCs	[263]
		scRNA‐seq	Adult mouse cardiac PCs	[46]
		scRNA‐seq	Human cardiac PCs	[52]
		scRNA‐seq	PCs in vascular organoids from non‐diabetic donors	[30]
	LOXL3	RT‐PCR	Adult rat HSCs	[263]
		scRNA‐seq	Human cardiac PCs	[52]
		scRNA‐seq	PCs in vascular organoids from non‐diabetic donors	[30]
	LOXL4	RT‐PCR	Adult rat HSCs	[263]
		scRNA‐seq	Human DCM cardiac PCs	[52]
	OGFOD1	scRNA‐seq	PCs in vascular organoids from non‐diabetic donors	[30]
	PLOD1	scRNA‐seq	Adult mouse brain PCs	[41]
		scRNA‐seq	Human cardiac PCs	[52]
		scRNA‐seq	PCs in vascular organoids from non‐diabetic donors	[30]
	PLOD2	scRNA‐seq	PCs in vascular organoids from non‐diabetic donors	[30]
	P4HA1	scRNA‐seq	Adult mouse brain PCs	[41]
		scRNA‐seq	Adult human brain PCs	[43]
		scRNA‐seq	PCs in vascular organoids from non‐diabetic donors	[30]
	P4HA2	Proteomics	Human brain PCs	[27]
	P4HA3	scRNA‐seq	Human brain PCs	[42]
	SULF1	scRNA‐seq	Human DCM cardiac PCs	[52]
		scRNA‐seq	PCs in vascular organoids from non‐diabetic donors	[30]
	SULF2	scRNA‐seq	PCs in vascular organoids from diabetic donors	[30]
	TGM1	scRNA‐seq	Human DCM cardiac PCs	[52]
	TGM2	Proteomics	Murine PC secretome	[28]
		Proteomics	Human placental PCs	[48]
		scRNA‐seq	Adult mouse brain PCs	[41]
		scRNA‐seq	Human cardiac PCs	[52]
	TLL2	scRNA‐seq	Human DCM cardiac PCs	[52]
Proteases	ADAM7	scRNA‐seq	Human DCM cardiac PCs	[52]
	ADAM8	scRNA‐seq	Human brain PCs	[42]
		scRNA‐seq	Human DCM cardiac PCs	[52]
	ADAM9	scRNA‐seq	Adult mouse cardiac PCs	[40]
		scRNA‐seq	PCs in vascular organoids from non‐diabetic donors	[30]
	ADAM10	scRNA‐seq	Adult mouse brain PCs	[41]
		scRNA‐seq	Adult mouse cardiac PCs	[40]
	ADAM11	scRNA‐seq	PCs in vascular organoids from non‐diabetic donors	[30]
	ADAM12	scRNA‐seq	PCs in vascular organoids from non‐diabetic donors	[30]
	ADAM15	scRNA‐seq	Human cardiac PCs	[52]
		scRNA‐seq	Adult mouse cardiac PCs	[40]
	ADAM17	scRNA‐seq	Adult mouse brain PCs	[41]
		scRNA‐seq	Human brain PCs	[42]
		scRNA‐seq	Adult mouse cardiac PCs	[40]
	ADAM19	scRNA‐seq	PCs in vascular organoids from non‐diabetic donors	[30]
	ADAM30	scRNA‐seq	PCs in vascular organoids from diabetic donors	[30]
	ADAM33	scRNA‐seq	Adult human brain PCs	[42, 43]
		scRNA‐seq	Human DCM cardiac PCs	[52]
	ADAMDEC1	scRNA‐seq	PCs in vascular organoids from non‐diabetic donors	[30]
	ADAMTS1	scRNA‐seq	Adult mouse cerebral artery	[98]
		scRNA‐seq	Adult mouse kidney PCs	[97]
		scRNA‐seq	Adult human pancreatic PCs	[76]
		scRNA‐seq	Adult mouse brain PCs	[41]
		scRNA‐seq	Adult mouse cardiac PCs	[40]
	ADAMTS2	scRNA‐seq	Adult mouse kidney PCs	[97]
		scRNA‐seq	Adult mouse cardiac PCs	[40]
		scRNA‐seq	PCs in vascular organoids from non‐diabetic donors	[30]
	ADAMTS3	scRNA‐seq	Adult mouse cardiac PCs	[40]
	ADAMTS4	scRNA‐seq	Adult mouse cerebral artery	[98]
		scRNA‐seq	Adult mouse kidney PCs	[97]
		scRNA‐seq	Adult human pancreatic PCs	[76]
		scRNA‐seq	Human cardiac PCs	[52]
		scRNA‐seq	PCs in vascular organoids from non‐diabetic donors	[30]
	ADAMTS5	scRNA‐seq	Adult mouse kidney PCs	[97]
		scRNA‐seq	PCs in vascular organoids from non‐diabetic donors	[30]
	ADAMTS6	scRNA‐seq	PCs in vascular organoids from non‐diabetic donors	[30]
	ADAMTS7	scRNA‐seq	Human brain PCs	[42]
	ADAMTS8	scRNA‐seq	Human brain PCs	[42]
	ADAMTS9	scRNA‐seq	Adult human pancreatic PCs	[76]
		scRNA‐seq	Human cardiac PCs	[52]
		scRNA‐seq	Adult mouse cardiac PCs	[40]
		scRNA‐seq	Adult human brain PCs	[43]
		scRNA‐seq	PCs in vascular organoids from non‐diabetic donors	[30]
	ADAMTS10	scRNA‐seq	Adult mouse brain PCs	[41]
		scRNA‐seq	Human brain PCs	[42]
	ADAMTS12	scRNA‐seq	Adult mouse cardiac PCs	[40]
	ADAMTS13	scRNA‐seq	Human brain PCs	[42]
	ADAMTS14	scRNA‐seq	Human brain PCs	[42]
	ADAMTS15	scRNA‐seq	Human brain PCs	[42]
	ADAMTS16	scRNA‐seq	Human brain PCs	[42]
	ADAMTS19	scRNA‐seq	PCs in vascular organoids from non‐diabetic donors	[30]
	ADAMTS20	scRNA‐seq	Human DCM cardiac PCs	[52]
	ADAMTSL1	scRNA‐seq	Human DCM cardiac PCs	[52]
	ADAMTSL4	scRNA‐seq	PCs in vascular organoids from non‐diabetic donors	[30]
	ADAMTSL5	scRNA‐seq	PCs in vascular organoids from non‐diabetic donors	[30]
	ASTL	scRNA‐seq	Human brain PCs	[42]
	CTSA	scRNA‐seq	Human brain PCs	[42]
	CTSB	scRNA‐seq	Adult mouse brain PCs	[41]
		scRNA‐seq	Human cardiac PCs	[52]
	CTSC	scRNA‐seq	Human cardiac PCs	[52]
		scRNA‐seq	PCs in vascular organoids from diabetic donors	[30]
	CTSD	Proteomics	Murine PC secretome	[28]
		scRNA‐seq	Adult human pancreatic PCs	[76]
		scRNA‐seq	Human brain PCs	[42]
		scRNA‐seq	Human cardiac PCs	[52]
	CTSF	scRNA‐seq	Adult mouse brain PCs	[41]
		scRNA‐seq	Human brain PCs	[42]
	CTSK	scRNA‐seq	Human brain PCs	[42]
	CTSL	scRNA‐seq	Adult mouse brain PCs	[41]
		scRNA‐seq	Human cardiac PCs	[52]
	CTSO	scRNA‐seq	Adult mouse brain PCs	[41]
		scRNA‐seq	Human brain PCs	[42]
	CTSS	scRNA‐seq	Mouse post‐natal retinal PCs	[51]
		scRNA‐seq	Adult mouse cardiac PCs	[40]
	CTSW	scRNA‐seq	Human DCM cardiac PCs	[52]
	CTSZ	scRNA‐seq	Adult mouse brain PCs	[41]
		scRNA‐seq	PCs in vascular organoids from non‐diabetic donors	[30]
	ELANE	scRNA‐seq	Human brain PCs	[42]
	HTRA1	Proteomics	Human brain PCs	[29]
		scRNA‐seq	Human brain PCs	[42]
		scRNA‐seq	PCs in vascular organoids from non‐diabetic donors	[30]
	MASP1	scRNA‐seq	PCs in vascular organoids from diabetic donors	[30]
	MASP2	Proteomics	Murine PC secretome	[28]
		scRNA‐seq	Human brain PCs	[42]
	MMP1	RT‐PCR	Human foetal brain PCs	[83]
		Proteomics	Human brain PCs	[29]
	MMP2	Immunoblot	Human brain PCs	[38]
		Proteomics	Human brain PCs	[29]
		Proteomics	Human placental PCs	[48]
		RT‐PCR	Human foetal brain PCs	[83]
		RT‐PCR	Human skeletal PCs	[116]
		RT‐PCR	Adult mouse cardiac PCs	[46]
		scRNA‐seq	Adult mouse kidney PCs	[97]
		scRNA‐seq	Human brain PCs	[42]
		scRNA‐seq	Adult mouse cardiac PCs	[40]
		zymography	Human retinal PCs	[117]
		zymography	Bovine retinal PCs	[118]
	MMP3	RT‐PCR	Embryonic mouse brain PCs	[119]
	MMP7	scRNA‐seq	Adult mouse kidney PCs	[97]
	MMP9	Immunoblot	Human brain PCs	[38]
		Immunoblot/ zymography	Rat brain PCs	[120]
		RT‐PCR	Embryonic mouse brain PCs	[119]
		RT‐PCR	Human skeletal PCs	[116]
		ScRNA‐seq	Adult mouse kidney PCs	[97]
		ScRNA‐seq	Adult mouse cardiac PCs	[40]
		ScRNA‐seq	PCs in vascular organoids from non‐diabetic donors	[30]
		zymography	Bovine retinal PCs	[118]
	MMP11	scRNA‐seq	Adult mouse kidney PCs	[97]
		scRNA‐seq	Human brain PCs	[42]
		scRNA‐seq	Adult mouse cardiac PCs	[40]
		scRNA‐seq	PCs in vascular organoids from non‐diabetic donors	[30]
	MMP13	RT‐PCR	Embryonic mouse brain PCs	[119]
	MMP14/MT1‐MMP	RT‐PCR	Embryonic mouse brain PCs	[119]
		RT‐PCR	Adult mouse cardiac PCs	[46]
		scRNA‐seq	Adult mouse kidney PCs	[97]
		scRNA‐seq	Adult mouse brain PCs	[41]
		scRNA‐seq	Human brain PCs	[42]
		scRNA‐seq	Adult mouse cardiac PCs	[40]
		scRNA‐seq	PCs in vascular organoids from non‐diabetic donors	[30]
	MMP15/MT2‐MMP	scRNA‐seq	Adult mouse brain PCs	[41]
		scRNA‐seq	Adult mouse cardiac PCs	[40]
		scRNA‐seq	PCs in vascular organoids from non‐diabetic donors	[30]
	MMP16/MT3‐MMP	scRNA‐seq	Human DCM cardiac PCs	[52]
		scRNA‐seq	Adult mouse cardiac PCs	[40]
		scRNA‐seq	PCs in vascular organoids from non‐diabetic donors	[30]
	MMP17/MT4‐MMP	scRNA‐seq	Adult mouse cardiac PCs	[40]
	MMP19	scRNA‐seq	Human brain PCs	[42]
		scRNA‐seq	PCs in vascular organoids from non‐diabetic donors	[30]
	MMP21	scRNA‐seq	Human DCM cardiac PCs	[52]
	MMP23	scRNA‐seq	Adult mouse kidney PCs	[97]
		scRNA‐seq	PCs in vascular organoids from non‐diabetic donors	[30]
	MMP25	scRNA‐seq	Human brain PCs	[42]
		scRNA‐seq	Human DCM cardiac PCs	[52]
	MMP26	scRNA‐seq	Human DCM cardiac PCs	[52]
	PAMR1	scRNA‐seq	Human brain PCs	[42]
		scRNA‐seq	PCs in vascular organoids from non‐diabetic donors	[30]
	PAPPA1	scRNA‐seq	PCs in vascular organoids from non‐diabetic donors	[30]
	PAPPA2	scRNA‐seq	Human DCM cardiac PCs	[52]
	PARM1	scRNA‐seq	PCs in vascular organoids from non‐diabetic donors	[30]
	plasminogen	Proteomics	Murine PC secretome	[28]
	thrombin	Proteomics	Human placental PCs	[48]
	TMPRSS15	scRNA‐seq	Human DCM cardiac PCs	[52]
	tPA	Proteomics	Human placental PCs	[48]
		ScRNA‐seq	Adult human pancreatic PCs	[76]
		scRNA‐seq	Adult mouse brain PCs	[41]
		scRNA‐seq	Embryonic mouse brain PCs	[45]
		scRNA‐seq	PCs in vascular organoids from diabetic donors	[30]
	uPA	Proteomics	Human brain PCs	[29]
		scRNA‐seq	Adult human pancreatic PCs	[76]
Protease inhibitors	A2M	scRNA‐seq	Adult human brain PCs	[43]
		scRNA‐seq	PCs in vascular organoids from non‐diabetic donors	[30]
	ITH1	Proteomics	Murine PC secretome	[28]
		scRNA‐seq	PCs in vascular organoids from non‐diabetic donors	[30]
	ITH2	Proteomics	Murine PC secretome	[28]
	ITH3	Proteomics	Murine PC secretome	[28]
		scRNA‐seq	Human brain PCs	[42]
	ITH4	scRNA‐seq	Human brain PCs	[42]
	ITIH5	scRNA‐seq	Adult mouse brain PCs	[41]
		scRNA‐seq	Embryonic mouse brain PCs	[45]
		scRNA‐seq	Adult human brain PCs	[43]
		scRNA‐seq	PCs in vascular organoids from non‐diabetic donors	[30]
	KAZALD1	scRNA‐seq	Human brain PCs	[42]
		scRNA‐seq	PCs in vascular organoids from diabetic donors	[30]
	PZP	Proteomics	Murine PC secretome	[28]
	SERPINA1	Proteomics	Murine PC secretome	[28]
	SERPINA3	Proteomics	Murine PC secretome	[28]
		scRNA‐seq	Human brain PCs	[42]
	SERPINA10	Proteomics	Murine PC secretome	[28]
	SERPINB1	scRNA‐seq	Human cardiac PCs	[52]
	SERPINB6	scRNA‐seq	Adult human pancreatic PCs	[76]
	SERPINC1	Proteomics	Murine PC secretome	[28]
		Proteomics	Human placental PCs	[48]
	SERPIND1	Proteomics	Murine PC secretome	[28]
	SERPINE1/PAI‐1	RT‐PCR	Adult mouse cardiac PCs	[46]
		scRNA‐seq	PCs in vascular organoids from non‐diabetic donors	[30]
	SERPINE2	scRNA‐seq	Adult mouse brain PCs	[41]
		scRNA‐seq	Embryonic mouse brain PCs	[45]
		scRNA‐seq	Human DCM cardiac PCs	[52]
		scRNA‐seq	Adult mouse cardiac PCs	[40]
	SERPINF1	Proteomics	Murine PC secretome	[28]
		scRNA‐seq	Human brain PCs	[42]
		scRNA‐seq	Human cardiac PCs	[52]
		scRNA‐seq	PCs in vascular organoids from non‐diabetic donors	[30]
	SERPINF2	Proteomics	Murine PC secretome	[28]
	SERPING1	Proteomics	Murine PC secretome	[28]
		scRNA‐seq	Adult mouse brain PCs	[41]
		scRNA‐seq	Human brain PCs	[42]
		scRNA‐seq	Human cardiac PCs	[52]
		scRNA‐seq	PCs in vascular organoids from non‐diabetic donors	[30]
	SERPINH1	scRNA‐seq	Embryonic mouse brain PCs	[45]
		scRNA‐seq	PCs in vascular organoids from non‐diabetic donors	[30]
	SERPINI1	scRNA‐seq	Adult mouse brain PCs	[41]
		scRNA‐seq	Human brain PCs	[42]
		scRNA‐seq	Human DCM cardiac PCs	[52]
		scRNA‐seq	PCs in vascular organoids from non‐diabetic donors	[30]
	TFPI2	Proteomics	Human placental PCs	[48]
	TIMP‐1	ELISA	Human post‐natal muscle PCs	[147]
		Immunoblot	Human brain PCs	[38]
		Proteomics	Human placental PCs	[48]
		RT‐PCR	Mouse/rat HSCs	[267]
		scRNA‐seq	Adult mouse cardiac PCs	[46]
		scRNA‐seq	Human cardiac PCs	[52]
		scRNA‐seq	PCs in vascular organoids from diabetic donors	[30]
	TIMP‐2	ELISA	Human retinal PCs	[139]
		RT‐PCR	Adult mouse cardiac PCs	[46]
		scRNA‐seq	Human brain PCs	[42]
	TIMP‐3	Immunoblot	Bovine retinal PCs	[118, 148]
		Proteomics	Murine PC secretome	[28]
		Proteomics	Human placental PCs	[48]
		scRNA‐seq	Adult mouse kidney PCs	[97]
		scRNA‐seq	Adult mouse cardiac PCs	[40]
		scRNA‐seq	Adult mouse brain PCs	[41]
		scRNA‐seq	Embryonic mouse brain PCs	[45]
		scRNA‐seq	Adult human brain PCs	[43]
		scRNA‐seq	Human cardiac PCs	[52]
		scRNA‐seq	PCs in vascular organoids from non‐diabetic donors	[30]
	TIMP‐4	scRNA‐seq	Human cardiac PCs	[52]
		scRNA‐seq	PCs in vascular organoids from non‐diabetic donors	[30]
Other ECM regulators	AGT	Immunoblot/RT‐PCR	Human brain PCs	[159]
		Proteomics	Murine PC secretome	[28]
		scRNA‐seq	Human brain PCs	[42]
		scRNA‐seq	Human cardiac PCs	[52]
		scRNA‐seq	Adult human pancreatic PCs	[76]
	HABP2	Proteomics	Murine PC secretome	[28]
	HRG	Proteomics	Murine PC secretome	[28]
	Kininogen 1	Proteomics	Murine PC secretome	[28]
Secreted factors	ANGPT1	ELISA	Human post‐natal muscle PCs	[147]
		scRNA‐seq	Adult mouse kidney PCs	[97]
		scRNA‐seq	Early postnatal mouse brain PCs	[50]
		scRNA‐seq	Human brain PCs	[42]
		scRNA‐seq	Adult human pancreatic PCs	[76]
		scRNA‐seq	PCs in vascular organoids from non‐diabetic donors	[30]
	ANGPT2	RT‐PCR	Human foetal brain PCs	[83]
		scRNA‐seq	Adult mouse kidney PCs	[97]
		scRNA‐seq	Adult human pancreatic PCs	[76]
		scRNA‐seq	Human brain PCs	[42]
		scRNA‐seq	Adult mouse cardiac PCs	[40]
	ANGPTL1	scRNA‐seq	PCs in vascular organoids from non‐diabetic donors	[30]
	ANGPTL2	scRNA‐seq	Early postnatal mouse brain PCs	[51]
		scRNA‐seq	Adult mouse brain PCs	[41]
		scRNA‐seq	Human brain PCs	[42]
	ANGPTL3	Proteomics	Murine PC secretome	[28]
		scRNA‐seq	PCs in vascular organoids from non‐diabetic donors	[30]
	ANGPTL4	Proteomics	Human brain PCs	[29]
		scRNA‐seq	Human brain PCs	[42]
		scRNA‐seq	PCs in vascular organoids from non‐diabetic donors	[30]
	ANGPTL5	scRNA‐seq	Human brain PCs	[42]
	ANGPTL6	scRNA‐seq	Human brain PCs	[42]
	AREG	scRNA‐seq	Human cardiac PCs	[52]
	ARTN	Proteomics	Human brain PCs	[29]
		scRNA‐seq	PCs in vascular organoids from non‐diabetic donors	[30]
	BDNF	Immunoblot/RT‐PCR	Human brain PCs	[159]
		scRNA‐seq	Human DCM cardiac PCs	[52]
		scRNA‐seq	PCs in vascular organoids from non‐diabetic donors	[30]
	BMP1	scRNA‐seq	Adult mouse brain PCs	[41]
		scRNA‐seq	PCs in vascular organoids from non‐diabetic donors	[30]
	BMP2	scRNA‐seq	PCs in vascular organoids from non‐diabetic donors	[30]
	BMP4	scRNA‐seq	Adult mouse brain PCs	[41]
	BMP5	scRNA‐seq	Early postnatal mouse brain PCs	[50]
		scRNA‐seq	Adult mouse brain PCs	[41]
	BMP6	scRNA‐seq	Human DCM cardiac PCs	[52]
	BMP8	scRNA‐seq	Human brain PCs	[42]
	CBLN3	scRNA‐seq	PCs in vascular organoids from non‐diabetic donors	[30]
	CCL2	ELISA	Human post‐natal muscle PCs	[147]
		Proteomics	Human brain PCs	[29]
		scRNA‐seq	Adult mouse cerebral artery	[98]
		scRNA‐seq	Human cardiac PCs	[52]
		scRNA‐seq	PCs in vascular organoids from diabetic donors	[30]
		scRNA‐seq	Human pancreatic PCs	[256]
	CCL3	ELISA	Mouse brain PCs	[138]
		scRNA‐seq	PCs in vascular organoids from non‐diabetic donors	[30]
	CCL4	ELISA	Mouse brain PCs	[138]
		Proteomics	Human brain PCs	[29]
		scRNA‐seq	PCs in vascular organoids from non‐diabetic donors	[30]
	CCL5	ELISA	Mouse brain PCs	[138]
		scRNA‐seq	Human DCM cardiac PCs	[52]
	CCL11	scRNA‐seq	Mouse brain PCs	[161]
		scRNA‐seq	Human pancreatic PCs	[256]
	CCL20	ELISA	Mouse brain PCs	[138]
	CCL25	Proteomics	Human brain PCs	[29]
	CCL27	scRNA‐seq	PCs in vascular organoids from non‐diabetic donors	[30]
	chordin	scRNA‐seq	Human brain PCs	[42]
		scRNA‐seq	PCs in vascular organoids from diabetic donors	[30]
	CRLF1	scRNA‐seq	Human brain PCs	[42]
	CRLF3	scRNA‐seq	PCs in vascular organoids from non‐diabetic donors	[30]
	CRHBP	scRNA‐seq	Human brain PCs	[42]
		scRNA‐seq	Human DCM cardiac PCs	[52]
	CSF1	Proteomics	Human brain PCs	[29]
		scRNA‐seq	Human brain PCs	[42]
		scRNA‐seq	Human cardiac PCs	[52]
		scRNA‐seq	PCs in vascular organoids from non‐diabetic donors	[30]
	CST3	scRNA‐seq	Human brain PCs	[42]
		scRNA‐seq	Human cardiac PCs	[52]
		scRNA‐seq	PCs in vascular organoids from diabetic donors	[30]
	CST5	Proteomics	Human brain PCs	[29]
	CSTB	scRNA‐seq	Human cardiac PCs	[52]
		scRNA‐seq	PCs in vascular organoids from diabetic donors	[30]
	CX3CL1	scRNA‐seq	Human brain PCs	[42]
	CXCL1	ELISA	Human post‐natal muscle PCs	[147]
		Proteomics	Human brain PCs	[29]
		scRNA‐seq	Human pancreatic PCs	[256]
	CXCL5	Proteomics	Human brain PCs	[29]
	CXCL6	Proteomics	Human brain PCs	[29]
	CXCL12	scRNA‐seq	Adult mouse brain PCs	[41]
		scRNA‐seq	Human cardiac PCs	[52]
		scRNA‐seq	PCs in vascular organoids from diabetic donors	[30]
		scRNA‐seq	Human pancreatic PCs	[256]
	CXCL13	scRNA‐seq	Adult human pancreatic PCs	[76]
		scRNA‐seq	Human pancreatic PCs	[256]
	CXCL16	scRNA‐seq	Human brain PCs	[42]
	CYR61	scRNA‐seq	Adult mouse brain PCs	[41]
		scRNA‐seq	Human DCM cardiac PCs	[52]
		scRNA‐seq	PCs in vascular organoids from non‐diabetic donors	[30]
	DMP1	scRNA‐seq	Early postnatal mouse brain PCs	[50]
	EDA	scRNA‐seq	Human cardiac PCs	[52]
	EGF	RT‐PCR	Human skeletal PCs	[116]
	EGFL6	scRNA‐seq	PCs in vascular organoids from non‐diabetic donors	[30]
	EGFL7	scRNA‐seq	Adult human pancreatic PCs	[76]
		scRNA‐seq	Adult human brain PCs	[43]
	EGFL8	scRNA‐seq	Human brain PCs	[42]
	FGF1	RT‐PCR	Human foetal brain PCs	[83]
		scRNA‐seq	Human DCM cardiac PCs	[52]
	FGF2	Immunoblot/RT‐PCR	Human brain PCs	[159]
		Proteomics	Human placental PCs	[48]
		scRNA‐seq	Human DCM cardiac PCs	[52]
	FGF7	scRNA‐seq	Human cardiac PCs	[52]
	FGF9	scRNA‐seq	Human DCM cardiac PCs	[52]
	FGF10	scRNA‐seq	Human cardiac PCs	[52]
	FGF11	scRNA‐seq	PCs in vascular organoids from non‐diabetic donors	[30]
	FGF13	scRNA‐seq	PCs in vascular organoids from diabetic donors	[30]
	FGF14	scRNA‐seq	Human DCM cardiac PCs	[52]
	FGF19	Proteomics	Human brain PCs	[29]
	FGF21	Proteomics	Human brain PCs	[29]
	FGF23	Proteomics	Human brain PCs	[29]
		scRNA‐seq	PCs in vascular organoids from diabetic donors	[30]
	FGFBP3	scRNA‐seq	PCs in vascular organoids from non‐diabetic donors	[30]
	FREM2	scRNA‐seq	Human DCM cardiac PCs	[52]
		scRNA‐seq	PCs in vascular organoids from diabetic donors	[30]
	FRZB	scRNA‐seq	Human brain PCs	[42]
		scRNA‐seq	PCs in vascular organoids from diabetic donors	[30]
	FSTL1	scRNA‐seq	Adult mouse cerebral artery	[98]
		scRNA‐seq	Embryonic mouse brain PCs	[45]
		scRNA‐seq	PCs in vascular organoids from non‐diabetic donors	[30]
	FSTL3	scRNA‐seq	Human cardiac PCs	[52]
		scRNA‐seq	PCs in vascular organoids from non‐diabetic donors	[30]
	GDF1	scRNA‐seq	Human brain PCs	[42]
	GDF5	scRNA‐seq	Human brain PCs	[42]
	GDF6	scRNA‐seq	PCs in vascular organoids from non‐diabetic donors	[30]
	GDF7	scRNA‐seq	PCs in vascular organoids from non‐diabetic donors	[30]
	GDF15	scRNA‐seq	Human brain PCs	[42]
		scRNA‐seq	PCs in vascular organoids from diabetic donors	[30]
	GDNF	Immunoblot/RT‐PCR	Human brain PCs	[159]
		Proteomics	Human brain PCs	[29]
	HCFC1	scRNA‐seq	Human cardiac PCs	[52]
	HCFC2	scRNA‐seq	PCs in vascular organoids from non‐diabetic donors	[30]
	HGF	ELISA	Human post‐natal muscle PCs	[147]
		ELISA	Human retinal PCs	[139]
		Proteomics	Human brain PCs	[29]
		RT‐PCR	Human skeletal PCs	[116]
		scRNA‐seq	Mouse adult HSCs	[162]
		scRNA‐seq	Human brain PCs	[42]
	IFNγ	ELISA	Mouse brain PCs	[138]
	IGF1	ELISA	Human post‐natal muscle PCs	[147]
		RT‐PCR	Human foetal brain PCs	[83]
	IGF2	Proteomics	Human placental PCs	[48]
		scRNA‐seq	Adult mouse brain PCs	[41]
		scRNA‐seq	Human brain PCs	[42]
		scRNA‐seq	PCs in vascular organoids from diabetic donors	[30]
	IL‐1	ELISA	Mouse brain PCs	[138]
		Proteomics	Human brain PCs	[29]
		scRNA‐seq	PCs in vascular organoids from non‐diabetic donors	[30]
	IL‐3	ELISA	Mouse brain PCs	[138]
	IL‐6	RT‐PCR	Human skeletal PCs	[116]
		Proteomics	Human brain PCs	[29]
		scRNA‐seq	Human brain PCs	[42]
		scRNA‐seq	Human pancreatic PCs	[256]
	IL‐7	scRNA‐seq	PCs in vascular organoids from non‐diabetic donors	[30]
	IL‐8	ELISA	Human retinal PCs	[139]
		Proteomics	Human brain PCs	[29]
		RT‐PCR	Human foetal brain PCs	[83]
	IL‐9	ELISA	Mouse brain PCs	[138]
	IL‐10	ELISA	Mouse brain PCs	[138]
		ELISA	Mouse/human retinal PCs	[160]
	IL‐11	scRNA‐seq	Adult mouse cerebral artery	[98]
	IL‐12	scRNA‐seq	PCs in vascular organoids from non‐diabetic donors	[30]
	IL‐13	ELISA	Mouse brain PCs	[138]
	IL‐15	scRNA‐seq	Human brain PCs	[42]
	IL‐17	scRNA‐seq	PCs in vascular organoids from non‐diabetic/diabetic donors	[30]
	IL‐24	scRNA‐seq	Adult human pancreatic PCs	[76]
	IL‐33	scRNA‐seq	Human brain PCs	[42]
		scRNA‐seq	Mouse pancreatic PCs	[257]
	IL‐34	scRNA‐seq	Adult mouse brain PCs	[41]
		scRNA‐seq	Human brain PCs	[42]
	INHBA	scRNA‐seq	Human brain PCs	[42]
	INHBB	scRNA‐seq	Adult human pancreatic PCs	[76]
	LEFTY2	scRNA‐seq	PCs in vascular organoids from non‐diabetic donors	[30]
		RT‐PCR	Human skeletal PCs	[116]
		scRNA‐seq	Human brain PCs	[42]
	MEGF8	scRNA‐seq	PCs in vascular organoids from non‐diabetic donors	[30]
	MEGF9	scRNA‐seq	Human DCM cardiac PCs	[52]
		scRNA‐seq	PCs in vascular organoids from non‐diabetic donors	[30]
	MEGF10	scRNA‐seq	Human brain PCs	[42]
	midkine	scRNA‐seq	Human brain PCs	[42]
		scRNA‐seq	PCs in vascular organoids from diabetic donors	[30]
	MIF	ELISA	Human post‐natal muscle PCs	[147]
	myostatin	Proteomics	Murine PC secretome	[28]
		Proteomics	Murine PC secretome	[28]
		scRNA‐seq	Human brain PCs	[42]
	Myostatin	scRNA‐seq	Human brain PCs	[42]
	NGF	RT‐PCR	Human brain PCs	[159]
		scRNA‐seq	Human cardiac PCs	[52]
		scRNA‐seq	PCs in vascular organoids from non‐diabetic donors	[30]
	NRG1	scRNA‐seq	Human DCM cardiac PCs	[52]
	NRG2	scRNA‐seq	Human brain PCs	[42]
	NTF3	scRNA‐seq	PCs in vascular organoids from non‐diabetic donors	[30]
	NTF4	scRNA‐seq	Human brain PCs	[42]
	PCSK6	scRNA‐seq	Human brain PCs	[42]
	PDGF	RT‐PCR	Human foetal brain PCs	[83]
		RT‐PCR	Human skeletal PCs	[116]
		scRNA‐seq	Adult human pancreatic PCs	[76]
		scRNA‐seq	Adult mouse brain PCs	[41]
		scRNA‐seq	PCs in vascular organoids from non‐diabetic donors	[30]
	PGF	scRNA‐seq	Adult human pancreatic PCs	[76]
		scRNA‐seq	Human cardiac PCs	[52]
	PIK3IP1	scRNA‐seq	Adult mouse brain PCs	[41]
		scRNA‐seq	Human brain PCs	[42]
		scRNA‐seq	PCs in vascular organoids from non‐diabetic donors	[30]
	PTN	scRNA‐seq	Adult mouse brain PCs	[41]
		scRNA‐seq	Human brain PCs	[42, 43]
		ScRNA‐seq	PCs in vascular organoids from diabetic donors	[30]
	SCUBE1	scRNA‐seq	Human DCM cardiac PCs	[52]
	SCUBE3	scRNA‐seq	PCs in vascular organoids from diabetic donors	[30]
	SFRP1	scRNA‐seq	Human brain PCs	[42]
		scRNA‐seq	PCs in vascular organoids from diabetic donors	[30]
	SFRP2	scRNA‐seq	Adult mouse brain PCs	[41]
		scRNA‐seq	PCs in vascular organoids from diabetic donors	[30]
	S100A1	scRNA‐seq	Adult mouse brain PCs	[41]
	S100A4	scRNA‐seq	Human cardiac PCs	[52]
		scRNA‐seq	PCs in vascular organoids from diabetic donors	[30]
	S100A6	scRNA‐seq	Human cardiac PCs	[52]
		scRNA‐seq	PCs in vascular organoids from diabetic donors	[30]
	S100A8	scRNA‐seq	Human cardiac PCs	[52]
	S100A9	scRNA‐seq	Human cardiac PCs	[52]
	S100A10	scRNA‐seq	Human cardiac PCs	[52]
		scRNA‐seq	PCs in vascular organoids from non‐diabetic donors	[30]
	S100A11	scRNA‐seq	Adult mouse brain PCs	[41]
		scRNA‐seq	Embryonic mouse brain PCs	[45]
		scRNA‐seq	Human cardiac PCs	[52]
		scRNA‐seq	PCs in vascular organoids from diabetic donors	[30]
	S100A13	scRNA‐seq	Human brain PCs	[42]
		scRNA‐seq	PCs in vascular organoids from diabetic donors	[30]
	S100Z	scRNA‐seq	Human DCM cardiac PCs	[52]
	TGF‐α	scRNA‐seq	Human DCM cardiac PCs	[52]
	TGF‐β1	Immunoblot	Human brain PCs	[38]
		Proteomics	Human brain PCs	[27]
		Immunoblot/RT‐PCR	Human brain PCs	[159]
		RT‐PCR	Human skeletal PCs	[116]
		RT‐PCR	Mouse/rat HSCs	[267]
		scRNA‐seq	Adult mouse brain PCs	[41]
		scRNA‐seq	Human brain PCs	[42]
		scRNA‐seq	PCs in vascular organoids from non‐diabetic donors	[30]
	TGF‐β2	Proteomics	Human placental PCs	[48]
		scRNA‐seq	Adult mouse brain PCs	[41]
		scRNA‐seq	PCs in vascular organoids from diabetic donors	[30]
	TGF‐β3	scRNA‐seq	Human brain PCs	[42]
		scRNA‐seq	Human cardiac PCs	[52]
	TNF‐α	ELISA	Mouse brain PCs	[138]
		Proteomics	Human brain PCs	[29]
	TNF‐β	ELISA	Human post‐natal muscle PCs	[147]
		Proteomics	Human brain PCs	[29]
	TNFSF8	scRNA‐seq	Human DCM cardiac PCs	[52]
	TNFSF11	scRNA‐seq	Human brain PCs	[42]
	TNFSF12	scRNA‐seq	Human brain PCs	[42]
	TNFSF15	scRNA‐seq	Human brain PCs	[42]
		scRNA‐seq	PCs in vascular organoids from non‐diabetic donors	[30]
	VEGF‐A	ELISA	Human post‐natal muscle PCs	[147]
		Immunoblot/RT‐PCR	Human brain PCs	[38, 159]
		Proteomics	Human brain PCs	[29]
		RT‐PCR	Human foetal brain PCs	[83]
		RT‐PCR	Human skeletal PCs	[116]
		scRNA‐seq	Human brain PCs	[42]
		scRNA‐seq	Adult mouse cardiac PCs	[40]
		scRNA‐seq	PCs in vascular organoids from non‐diabetic donors	[30]
	VEGF‐B	scRNA‐seq	Human brain PCs	[42]
		scRNA‐seq	Adult mouse cardiac PCs	[40]
		scRNA‐seq	PCs in vascular organoids from non‐diabetic donors	[30]
	VEGF‐C	scRNA‐seq	Human DCM cardiac PCs	[52]
		scRNA‐seq	Adult mouse cardiac PCs	[40]
	VEGFD	scRNA‐seq	Human brain PCs	[42]
	WFIKKN1	scRNA‐seq	Human DCM cardiac PCs	[52]
	WNT5A	scRNA‐seq	PCs in vascular organoids from non‐diabetic donors	[30]
	WNT6	scRNA‐seq	Adult human pancreatic PCs	[76]
		scRNA‐seq	Human cardiac PCs	[52]
	WNT11	scRNA‐seq	Human brain PCs	[42]
		scRNA‐seq	PCs in vascular organoids from diabetic donors	[30]
	WNT16	scRNA‐seq	Human brain PCs	[42]
	ZCP91	scRNA‐seq	PCs in vascular organoids from non‐diabetic donors	[30]

A2M, alpha 2 macroglobulin; ADAM, ADAM metallopeptidase domain; ADAMDEC1, ADAM‐like, decysin 1; ADAMTS, ADAM metallopeptidase with thrombospondin type 1 motif; ADAMTSL, ADAMTS‐like; AEBP1, AE binding protein 1; AGT, angiotensinogen; ANGPT1, Angiopoietin‐1; ANGPTL, angiopoietin‐like; ANXA, annexin A; ANXA8L1, annexin A8‐like 1; AREG, amphiregulin; ARTN, artemin; ASPN, asporin; ASTL, astacin‐like metallo‐endopeptidase (M12 family); BDNF, brain‐derived neurotrophic factor; BMP, bone morphogenetic protein; BMPER, BMP‐binding endothelial regulator; C1QC, complement component 1, q subcomponent; C1QL1, complement component 1, q subcomponent‐like; C1QTNF, C1q and tumor necrosis factor related protein; CBLN3, cerebellin 3 precursor; CILP, cartilage intermediate layer protein; CCL, chemokine (C‐C motif) ligand; CLEC, C‐type lectin domain family; COLEC, collectin sub‐family member; COMP, cartilage oligomeric matrix protein; CRELD, cysteine‐rich with EGF‐like domains; CRHBP, corticotropin releasing hormone binding protein; CRIM1, cysteine rich transmembrane BMP regulator 1 (chordin‐like); CRISPLD, cysteine‐rich secretory protein LCCL domain containing; CRLF, cytokine receptor‐like factor; CSF1, colony stimulating factor 1; CSPG4, chondroitin sulfate proteoglycan 4; CST, cystatin; CTF1, cardiotrophin 1; CTGF, connective tissue growth factor; CTHRC1, collagen triple helix repeat containing 1; CTS, cathepsin; CX3CL1, chemokine (C‐X3‐C motif) ligand 1; CXCL, chemokine (C‐X‐C motif) ligand; CYR61, cysteine‐rich, angiogenic inducer, 61; DCM, dilated cardiomyopathy; DMBT1, deleted in malignant brain tumors 1; DMP1, dentin matrix acidic phosphoprotein 1; ECM, extracellular matrix protein; EDA, ectodysplasin A; EDIL3, EGF‐like repeats and discoidin I‐like domains 3; EFEMP2, EGF‐containing fibulin‐like extracellular matrix protein 2; EGF, epidermal growth factor; EGFL, EGF‐like‐domain, multiple; EGFLAM, EGF‐like, fibronectin type III and laminin G domains; EGLN, egl nine homolog; ELANE, neutrophil elastase; ELFN, extracellular leucine‐rich repeat and fibronectin type III domain containing, EMCN, endomucin; EMID1, EMI domain containing 1; EMILIN1, elastin microfibril interfacer 1; ESM1, endothelial cell‐specific molecule 1; FACS, fluorescence activated cell sorting; FAM20, family with sequence similarity 20; FBN, fibrillin; FBLN, fibulin; FCN, ficolin; FGFBP3, fibroblast growth factor binding protein 3; FN1, fibronectin 1; FRAS1, Fraser syndrome 1; FREM2, FRAS1 related extracellular matrix protein 2; FRZB, frizzled‐related protein; FSTL, Follistatin like; GAS6, growth arrest‐specific 6; GDF, growth differentiation factor; GDNF, glial cell derived neurotrophic factor; GPC, glypican; HABP, hyaluronan binding protein; HAPLN, hyaluronan and proteoglycan link protein; HCFC, host cell factor C; HGF, hepatocyte growth factor; HMCN, hemicentin; HRG, histidine‐rich glycoprotein; HSCs, hepatic stellate cells; HSPG2, heparan sulfate proteoglycan 2; HTRA1, HtrA serine peptidase 1; HYAL2, hyaluronoglucosaminidase 2; IFN, interferon; IGF, insulin‐like growth factor; IGFBP, insulin growth factor binding protein; IHC, immunohistochemistry; IL, Interleukin; IMPG, interphotoreceptor matrix proteoglycan; INHB, inhibin beta; ISH, in situ hybridization; ITIH, inter‐alpha (globulin) inhibitor H; KAZALD1, Kazal‐type serine peptidase inhibitor domain 1; LAMA, laminin α chain; LAMB, laminin β chain; LAMC, laminin γ chain; LEFTY 2, left‐right determination factor 2; LGALS, lectin, galactoside‐binding, soluble; LGI2, leucine‐rich repeat LGI family member 2; LIF, leukemia inhibitory factor; LMAN1, lectin, mannose‐binding 1; LOXL, lysyl oxidase‐like; LTBP, latent transforming growth factor beta binding protein; MASP, mannan‐binding lectin serine peptidase 1 (C4/C2 activating component of Ra‐reactive factor); MEGF, multiple EGF‐like‐domains; MFAP, microfibril associated protein; MFGE8, milk fat globule‐EGF factor 8 protein; MGP, matrix Gla protein; MIF, Macrophage migration inhibitory factor; MMP, matrix metalloproteinase; MMRN2, multimerin 2; MXRA5, matrix‐remodelling associated 5; NG2, neuron‐glial antigen 2; NGF, nerve growth factor; NPNT, nephronectin; NRG, neuregulin; NTF, neurotrophin; NTN, netrin; OGFOD1, 2‐oxoglutarate and iron‐dependent oxygenase domain containing 1; OIT3, oncoprotein induced transcript 3; OVGP1, oviductal glycoprotein 1, 120 kDa; P4HA, prolyl 4‐hydroxylase, alpha polypeptide; PAI‐1, plasminogen activator inhibitor 1; PAMR1, peptidase domain containing associated with muscle regeneration 1; PAPPA, pappalysin; PARM1, prostate androgen‐regulated mucin‐like protein 1; PC, pericyte; PCOLCE, procollagen C‐endopeptidase enhancer; PCSK6, proprotein convertase subtilisin/kexin type 6; PDGF, platelet‐derived growth factor; PGF, placental growth factor; PIK3IP1, phosphoinositide‐3‐kinase interacting protein 1; PLOD, procollagen‐lysine, 2‐oxoglutarate 5‐dioxygenase 1; PRG4, p53‐responsive gene 4; PLXDC, plexin domain containing; PLXN, plexin; PODNL1, podocan‐like 1; POMZP3, POM (POM121 homolog, rat) and ZP3 fusion; PRELP, proline/arginine‐rich end leucine‐rich repeat protein; PTN, pleiotrophin; PXDN, peroxidasin homolog; PXDNL, peroxidasin homolog‐like; PZP, pregnancy‐zone protein; S100, S100 calcium binding protein; scRNA‐seq, single‐cell RNA sequencing; SCUBE, signal peptide, CUB domain, EGF‐like; SDC, syndecan; SEMA, semaphorin; SERPIN, serpin peptidase inhibitor; SFRP, secreted frizzled‐related protein; SLIT, slit homolog; SMOC, SPARC‐related modular calcium‐binding protein; SNED1, sushi, nidogen and EGF‐like domains 1; SPARC, secreted protein, acidic, cysteine‐rich (osteonectin); SPARCL1, SPARC‐like 1 (hevin); SPOCK2, sparc/osteonectin, cwcv and kazal‐like domains proteoglycan (testican); SPON, spondin; SPP1, secreted phosphoprotein 1/osteopontin; SULF, sulfatase; SVEP1, sushi, von Willebrand factor type A, EGF and pentraxin domain containing 1; TFPI, tissue factor pathway inhibitor; TGF, transforming growth factor; TGM2, transglutaminase 2 (C polypeptide, protein‐glutamine‐gamma‐glutamyltransferase); THBS, thrombospondin; TIMP, tissue inhibitor of metalloproteinase; TINAGL1, tubulointerstitial nephritis antigen‐like 1; TLL2, tolloid‐like 2; TSKU, tsukushi small leucine rich proteoglycan homolog; TNC, tenascin C; TNR, tenascin R; TNX, tenascin X; TNF, tumor necrosis factor; TNFAIP6, tumor necrosis factor, alpha‐induced protein 6; TNFSF, tumor necrosis factor (ligand) superfamily; tPA, tissue plasminogen activator; uPA, urokinase plasminogen activator; VEGF, vascular endothelial growth factor; VTN, vitronectin; vWA5A, von Willebrand Factor A domain containing 5; vWF, von Willebrand Factor; WFIKKN1, WAP, follistatin/kazal, immunoglobulin, kunitz and netrin domain containing 1; WISP, WNT1 inducible signaling pathway protein; WNT, wingless‐type MMTV integration site family; ZFP91, zinc finger protein 1 homolog; ZP3, zona pellucida glycoprotein 3; ZPLD1, zona pellucida‐like domain containing 1.

Proteomics, bulk‐ and single‐cell transcriptomics (sc‐RNAseq) studies have contributed to define the PC matrisome and its pathophysiological changes in composition both in monocultures and spheroid models, although the integration of different datasets remains challenging. Emerging technologies based on the anatomical mapping of transcriptomic data (spatial transcriptomics) hold the exciting potential of distinguishing PCs by their anatomical location, independent of the marker expression; however, the resolution is still often too low to reliable differentiate PCs from ECs [26]. Since PCs, ECs, and other cell types such as ACs are intimately connected, elucidation of the PC matrisome by proteomics is quite challenging when co‐cultures or organoids are used. On the other hand, the matrisome of PC monocultures may not reflect *in vivo* conditions due to PC/EC crosstalk, which is known to affect the PC secretome [27]. To address this issue, some proteomic approaches have utilised specific labels (for example biotin) to distinguish the PC contribution to the proteome of co‐cultures and organoids [28, 29]. Sc‐RNAseq, however, does not suffer from the limitations of proteomics and has enabled PC profiling in vascular organoids [30]. Technical challenges aside, there is undoubtedly a large overlap between the matrisome of PCs, ECs, and other perivascular cells. Despite these limitations, critically defining the PC matrisome is a crucial step that has not been highlighted in the literature, and here we aim to fill this gap.

We base our classification of the matrisome categories to those listed by Naba *et al*. [31] as updated in the Matrisome Project website (https://sites.google.com/uic.edu/matrisome/) and refer to the Basement membraneBASE (https://bmbase.manchester.ac.uk/) for specific BM components.

### Core Matrisome components

These include the structural proteins collagens, proteoglycans and other ECM glycoproteins.

#### Collagens

Collagens are trimeric proteins highly abundant in proline and hydroxyproline. The abundance of these two residues is required for triple helical assembly. This typical molecular architecture is responsible for tensile strength and indeed collagens are major determinants of biomechanical properties in numerous tissues.

In humans, the collagen family comprises 28 members [32]. Collagen IV, a main collagen in BM, is composed of 6 α(IV) chains, each one comprising a cysteine‐rich 7S N‐terminal domain, a triple helical domain and a globular C‐terminal non‐collagenous (NC1) domain. Due to the absence of a glycine at every third residue within the Gly‐X‐Y repeat required for triple helix stabilisation (where X is generally proline and Y 4‐hydroxyproline), collagen IV forms triple helices that are more flexible than those of fibrillar collagens [15] (Fig. 2). Collagen IV forms hexameric networks composed of a tetramer assembled through the amino terminal 7S domain and a dimer assembled through the carboxy‐terminal NC1 domain [32]. Mutations in collagen IV α(I) and α(II) chains cause several inherited human disorders affecting the kidney, eye and brain, including porencephaly and Hereditary angiopathy with nephropathy, aneurysm and cramps, which are partially phenocopied in mice with missense mutations either in *Col4α1* or *Col4α2* (summarised in [33]).

Collagen type IV is cleaved by MMP2 and MMP9 (also known as 72‐kDa and 92‐kDa gelatinases respectively) [32]. Proteolysis of collagen type IV generates a number of fragments with biological activities (matrikines) [34]. These include tumstatin, the NC1 domain of the collagen α3 chain, generated by MMP9 [35], and tetrastatin, the NC1 domain of collagen type IV α4 chain [36], acting as inhibitors of tumour growth, angiogenesis and EC proliferation [37]. In addition to collagen type IV [29, 30, 38, 39, 40, 41, 42, 43, 44, 45, 46, 47, 48, 49], PCs secrete the IM fibrillar collagens type I [3, 27, 29, 30, 39, 40, 43, 44, 45, 48, 49, 50, 51, 52], III [27, 30, 39, 40, 42, 44, 45, 46, 48, 50], V [27, 30, 39, 40, 42, 43, 44, 46, 52] and VI [30, 39, 40, 41, 42, 44, 46, 50] (Table 1). Collagen type I is cleaved by secreted proteases MMP1 (collagenase I/interstitial collagenase), MMP8 (collagenase 2/neutrophil collagenase), MMP13 (collagenase 3) and the membrane‐anchored MMP14 (MT1‐MMP) [53].

#### Proteoglycans

Proteoglycans are composed of a protein core decorated with one or more glycosaminoglycan (GAG) chains (chondroitin/dermatan sulfate, keratan sulfate or heparan sulfate). Based on their cellular location, proteoglycans can be classified into intracellular, cell surface (transmembrane), pericellular/BM and extracellular [54].

Neuron‐glial antigen 2 (NG2), also known as chondroitin sulfate proteoglycan‐4 (CSPG4), is one of the most established PC markers [55]. It is a 450 kDa type I transmembrane chondroitin sulfate proteoglycan composed of an extracellular domain, a type I transmembrane region (25 residues) and a cytoplasmic tail (75 residues) containing multiple potential threonine phosphorylation sites [56]. The extracellular domain consists of 3 subdomains (D1‐D3) involved in the binding of other ECM components including collagens type II, IV and VI, laminins, tenascins and fibronectin [56]. The interaction between the chondroitin sulfate GAG on NG2/CSPG4 and the transmembrane protease MMP16/MT3‐MMP leads to MMP2 activation and may therefore be part of the mechanisms leading to localised gelatinolytic activity by PCs [57]. NG2/CSPG4 may play a role in PC proliferation, motility, angiogenesis and crosstalk with ECs [58]. NG2/CSPG4 acts as a cell surface sequestrator of growth factors like fibroblast growth factor (FGF)‐2 and PDGF‐α/β. PDGF‐α and PDGF‐β form homo‐ or hetero‐dimers which activate PDGFRα/β on the surface of PCs, resulting in receptor autophosphorylation and activation of downstream signalling pathways including phosphoinositide 3‐kinase and RAS/mitogen‐activated protein kinase. The direct interaction between NG2/CSPG4 and FGF‐2/PDGF‐α, unusually mediated by the proteoglycan protein core [59], is responsible for the enhanced proliferative response of PCs to paracrine and autocrine stimulation by these growth factors [60]. NG2/CSPG4 also establishes interactions with integrins on the EC surface [61, 62] and intracellularly with the actin cytoskeleton [63, 64], thus helping to establish and maintain the interactions between PCs and ECs. For example, the binding of NG2/CSPG4 to β1 integrin triggers integrin activation leading to cell proliferation and motility [61]. NG2/CSPG4 levels are highly upregulated during vascular remodelling or angiogenesis (reviewed in [58]). In addition to PCs, NG2/CSPG4 is expressed by other cardiovascular cell types such as cardiomyocytes and arterial smooth muscle cells during development [65].

Syndecan‐2 is a heparan sulfate transmembrane proteoglycan expressed by PCs [27, 30, 50, 66] known to promote angiogenesis in microvascular ECs through its ectodomain [67].

In addition to cell‐bound proteoglycans, several pericellular/extracellular proteoglycans are secreted by PCs (Table 1). Perlecan, a major BM component, is a heparan sulfate proteoglycan with 3 N‐terminal GAG chains forming complex supramolecular assemblies with a variety of ligands including collagen type IV, laminins, fibronectin and fibulins through their extracellular domains [68] (Fig. 2). Perlecan plays an important structural role in the BM by acting as a bridging molecule between the networks formed by collagen type IV and laminin [69]. Perlecan‐null mice die *in utero* and perinatally due to myocardial defects, but their BM appears normal, probably because of compensation by other heparan sulfate proteoglycans [70]. The C‐terminal proteolytic fragment of perlecan, endorepellin, generated by multiple proteases [71], is an anti‐angiogenic matrikine which binds simultaneously to integrin α2β1 and vascular endothelial growth factor (VEGF) receptor 2 (VEGFR2) on ECs [72]. Binding of endorepellin to integrin α2β1 induces the tyrosine phosphatase SHP‐1 that inactivates VEGFR [72, 73], therefore, this matrikine acts as a dual receptor antagonist of VEGF. Together with other heparan sulfate proteoglycans, collagen type XVIII and agrin, perlecan also acts as a reservoir of secreted factors and cytokines [74]. Collagen type XVIII retains features of both collagens and proteoglycans due to the presence of heparan sulfate GAG chains [54]. Collagen type XIII is abundantly expressed in the BM [75] and is secreted by PCs, particularly in the pancreas and CNS [30, 39, 41, 42, 43, 52, 76] (Table 1). In apolipoprotein E‐deficient mice, genetic deletion of the gene coding for collagen type XVIII α1 chain (*Col18a1*) results in increased intimal neovascularisation, indicating an anti‐angiogenic effect of this proteoglycan [77]. This is further supported by the persistence of hyaloid vasculature in the vitreous body of *Col18a1* null mice after development, a manifestation of Knobloch syndrome in humans, a rare genetic disorder characterised by severe eye abnormalities (reviewed in [33]). Cleavage of the region between the C‐terminal and the triple helical domains in collagen type XVIII releases a fragment with anti‐angiogenic activity, endostatin [78].

Fibromodulin and osteoglycin (Table 1) have been proposed as distinctive markers of PCs [66]. Fibromodulin is a keratan sulfate small leucine‐rich proteoglycan (SLRP) involved in collagen fibril formation [79] and a substrate of PC‐expressed A Disintegrin‐Like and Metalloproteinase with Thrombospondin motifs 4 (ADAMTS4) [80]. Osteoglycin, also known as mimecan, is another keratan sulfate SLRP, involved, like fibromodulin, in collagen fibril formation [81]. Osteoglycin is proteolytically processed into its mature, functionally competent form by bone morphogenetic protein 1 (BMP1)/Tolloid‐related metalloproteinases BMP‐1, mTLD, mTLL‐1 and mTLL‐2 [82]. Mouse pancreatic PCs express decorin, biglycan, lumican, osteoglycin and podocan, with decorin being the more abundantly expressed [39] (Table 1). The unsulfated GAG hyaluronan is a component of the IM (Fig. 2), and pancreatic PCs express hyaluronan synthase 1, 2 and 3 [39].

#### ECM glycoproteins

Among the PC‐expressed proteins in this category there are the IM components fibronectin [28, 30, 38, 40, 42, 44, 48, 49, 83, 84], elastin [39, 48], tenascin C [27, 29, 40, 46, 48] and vitronectin [28, 41, 44, 45, 48, 50, 84, 85], and the BM components laminins [30, 39, 41, 42, 43, 44, 45, 47, 48, 50, 51, 52, 76] and nidogens [30, 39, 41, 43, 45, 52, 83]. Fibronectin is frequently used as a substrate in *in vitro* PC cultures and induces higher rates of proliferation and migration than heparan sulfate proteoglycan (HSPG) or laminin‐1 [86]. Vitronectin is an adhesion protein binding multiple ligands such as collagens, plasminogen, plasminogen activator inhibitor 1 (PAI‐1) and urokinase receptor (uPAR). Vitronectin adhesion properties are mediated by its Arg‐Gly‐Asp (RGD) integrin‐binding sequence [87].

Laminins are high molecular weight (400–800 kDa) heterotrimeric glycoproteins which initiate BM assembly by interacting with collagen type IV through nidogens and perlecan [88] (Fig. 2). Laminins consist of an α chain, a β chain and a γ chain. Each chain has an N‐terminal region consisting of several epidermal growth factor (EGF)‐like motifs and globular domains (Fig. 2). From the combination of 5 α, 4 β and 3 γ chains, 16 different laminin isoforms arise [88]. Major laminin isoforms in the vascular BM are 111, 211, 411 and 511 [89]. Loss of PC laminin 411 in mice is associated with hydrocephalus and disruption of the BBB in 10% of the mutants [90]. Laminin α5 is a major component of the BM and, through binding to integrin β1, mediates EC adhesion. The interaction between laminin α5 and integrin β1 promotes the association of p120 catenin with VE‐cadherin, further stabilising EC adhesion [91]. A number of proteases share the ability to cleave laminins, including plasmin, MMPs (in particular MMP2 and MMP14/MT1‐MMP) and BMP1 [12].

Nidogens are 150 kDa glycoproteins acting as BM stabilisers through interactions with collagen type IV and laminins [69]. They consist of 3 globular domains, two situated at the N‐terminus (G1 and G2) and one at the C terminus, separated by a central domain containing EGF repeats (Fig. 2). Both nidogens, nidogen 1 (entactin 1) and nidogen 2 (entactin 2), are expressed by brain and pancreatic PCs [30, 39, 41, 43, 45, 52] (Table 1). In mice, global knockout of both genes results in perinatal lethality due to severe defects in BM assembly [92]. In addition to laminins and collagen type IV, nidogen 1 also binds to fibulin‐2 and perlecan [93, 94]. Nidogens are cleaved by ADAMTS1 [95, 96], which is also expressed by PCs [40, 41, 76, 97, 98] (Table 1).

SPARC‐related modular calcium binding 1 (SMOC1) is a member of the secreted protein, acidic, and rich in cysteine (SPARC)‐related modular calcium‐binding protein family with distinctive higher expression in PCs compared to ECs and smooth muscle cells [66, 99]. SMOC1 has been shown to promote EC proliferation and angiogenesis [100]. Another class of ECM glycoproteins expressed by PCs are the netrins [41, 42, 50, 52, 76] (Table 1), structurally related to laminins and involved in several developmental processes, especially in the CNS [101]. Netrin‐4 decreases the stiffness of the laminin network, making it more resistant to breaching by cancer cells, as demonstrated by increased metastasis formation in netrin null mice [102].

### Matrisome‐associated proteins

This category includes ECM‐affiliated proteins, ECM regulators and secreted factors.

#### ECM‐affiliated proteins

Among the PC‐expressed proteins in this category are annexins [30, 41, 42, 45, 52, 76], plexins [30, 42, 50, 52, 103, 104] and semaphorins [30, 42, 50, 52] (Table 1). Annexins are anti‐inflammatory cytosolic proteins lacking a classical signal peptide for secretion but that are present in the ECM as a result of yet uncharacterised processes, including neutrophil degranulation and exosomal/extravesicular secretion [105]. Annexin A2, expressed in human placental PCs [48], binds plasminogen, thus leading to its conversion to plasmin by tissue plasminogen activator (tPA) and thereby orchestrating fibrin dissolution [106]. Semaphorins include soluble and transmembrane members, all characterised by an N‐terminal extracellular sema domain followed by a plexin‐semaphorin‐integrin domain [107]. Plexins are type 1 single transmembrane‐spanning proteins acting as cell surface receptors for semaphorins and the two classes of proteins regulate developmental processes mainly but not limited to the CNS [108]. Among the PC‐expressed semaphorins [30, 42, 50, 52] (Table 1), class 3 semaphorins exhibit anti‐angiogenic activity by competing with VEGF for binding to neuropilin receptors [107], while semaphorin 5A promotes angiogenesis by activating signalling of the hepatocyte growth factor (HGF)/scatter factor receptor Met through plexin B3 binding [109]. Semaphorin 6A inhibits EC apoptosis through VEGF/VEGFR2 signalling [110].

#### ECM regulators

These comprise ECM‐modifying enzymes, proteases and protease inhibitors.

#### ECM‐modifying enzymes

Brain PCs express the three members of Prolyl‐4‐hydroxylase subunit alpha (P4HA) family [27, 30, 41, 42, 43] (Table 1), a class of enzymes which stabilise collagen fibres through the generation of 4‐hydroxyproline [111]. Other PC enzymes involved in the post‐translational modification of collagens are the lysyl oxidases (LOXs) [30, 46] and the LOX‐Like (LOXL) [30, 46, 52], copper‐containing amine oxidases responsible for the specific crosslinking of collagens and elastin [112], and transglutaminase 2 (TGM2) [28, 48, 52], which catalyses the formation of an intermolecular isopeptide bond between glutamine and lysine residues [113]. Upregulated activity of LOXs, LOXLs and TGM2 is directly linked to fibrosis [114, 115].

#### Proteases

PC‐expressed proteases include the gelatinases MMP2 [29, 38, 40, 42, 46, 48, 83, 97, 116, 117, 118] and MMP9 [30, 38, 40, 97, 116, 118, 119, 120], the serine proteases High temperature requirement A1 (HTRA1) [29, 30, 42], plasmin [28], urokinase‐type plasminogen activator (uPA) [29, 76] and several cathepsins [28, 30, 41, 42, 51, 52] (Table 1). uPA is a serine protease that converts inactive plasminogen to plasmin, which is responsible for fibrinolysis [121]. Plasmin in turn proteolytically activates several MMPs, including MMP9, and therefore, uPA has the potential to trigger proteolytic cascades on the cell surface [122].

**Fig. 4:**
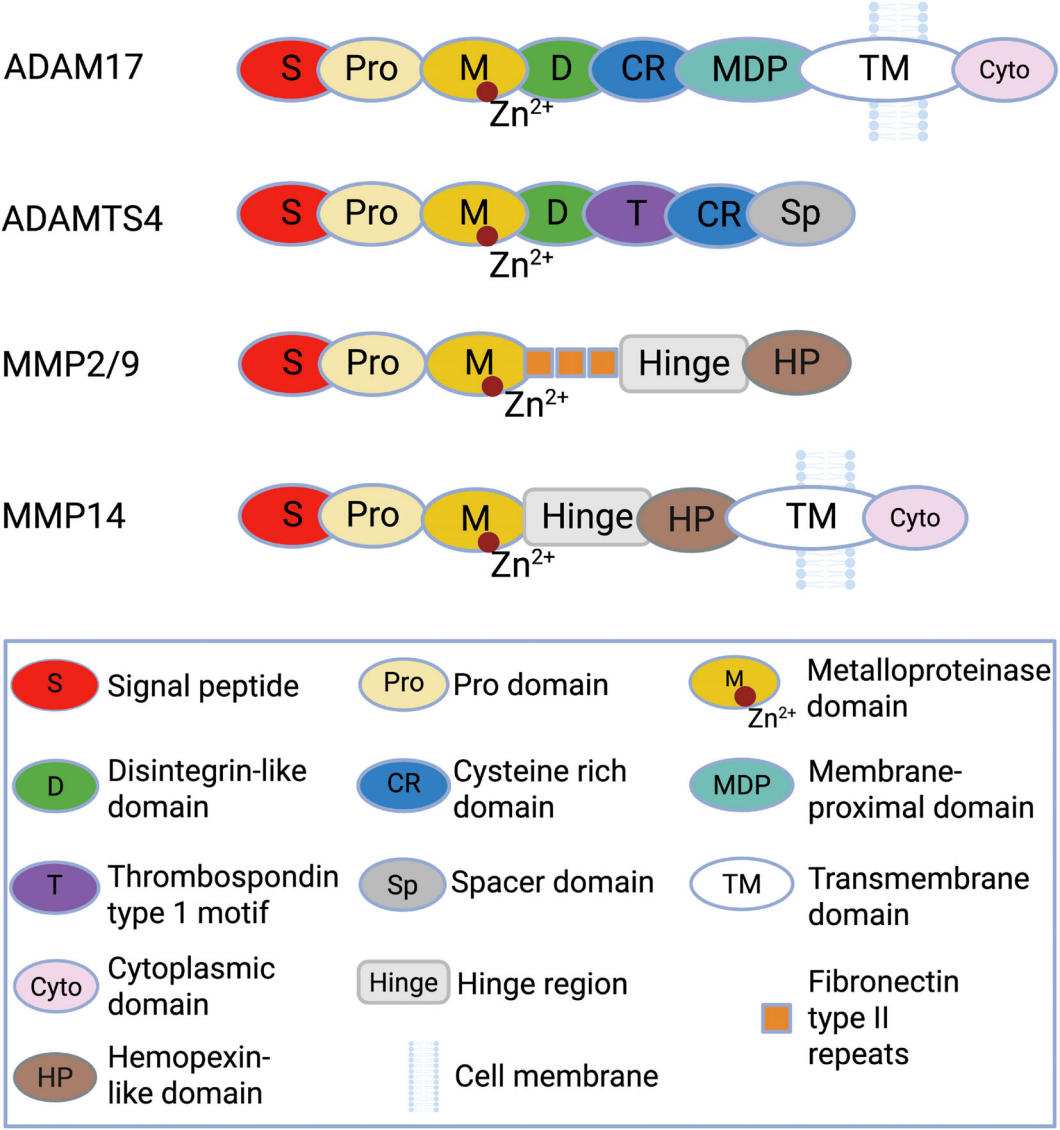
Domain organisation of representative members of the ADAM, ADAMTS and MMP metzincin families. All metzincins are secreted proteases expressed with a prodomain, necessary for enzyme latency, and the metalloproteinase domain where the catalytic zinc (coordinated by three histidine residues) is located. Matrix metalloproteinases (MMPs) and A Disintegrin‐Like and Metalloproteinase with Thrombospondin motifs (ADAMTSs) are secreted proteases, while A Disintegrin‐like metalloproteinases (ADAMs) are transmembrane proteases. Figure created with BioRender.

MMPs, ADAMTSs and A Disintegrin‐like metalloproteinases (ADAMs) are calcium‐dependent zinc‐endopeptidases belonging to the metzincin superfamily, characterised by three histidine residues coordinating a catalytic Zn^2+^ and a conserved methionine residue [123] (Fig. 4).

In human, there are 27 MMPs, divided into 6 subfamilies including collagenases (MMP1, MMP8 and MMP13), gelatinases (MMP2 and MMP9), stromelysins (MMP3, MMP10 and MMP11), matrilysins (MMP7 and MMP26) and membrane type MMPs (MMP14/MT1‐MMP, MMP15/MT2‐MMP, MMP16/MT3‐MMP, MMP17/MT4‐MMP, MMP24/MT5‐MMP and MMP25/MT6‐MMP). MMPs are expressed in a latent/zymogen form where removal of the prodomain is required for full proteolytic activity. Activation can occur either intra‐ or extracellularly and can be mediated by other proteases (furin, plasmin, other MMPs) in complex proteolytic cascades or autocatalytically through the action of reactive oxygen species, pH or hypoxia [124, 125]. All MMPs consists of a prodomain, a catalytic domain containing the metzincin signature, and, with the exception of matrilysins, an hemopexin domain. MMP2 and MMP9 are named gelatinases based on their ability to degrade gelatin, that is, denatured collagens. Specificity for gelatin is dictated by 3 fibronectin type II repeats inserted into their metalloproteinase catalytic domain [126] (Fig. 4). In addition, gelatinases degrade various other BM components, thus facilitating the migration of PCs and ECs and the formation of sprouting neovessels. PCs also express MMP14/MT1‐MMP [30, 40, 41, 42, 46, 97, 119] (Table 1), which mediates MMP2 proteolytic activation in complex with tissue inhibitor of metalloproteinase 2 (TIMP‐2) [127].

The ADAMTS metzincin subfamily includes 19 members widely involved in ECM remodelling [128]. The minimal ADAMTS domain organisation comprises a signal peptide, a prodomain, a metalloproteinase catalytic domain with conserved metzincin signature, a disintegrin‐like domain, and a number of ancillary domains including thrombospondin motif, cysteine‐rich and spacer (Fig. 4). Based on substrate specificity and phylogenetic relationships, ADAMTSs are classified into proteoglycanases (ADAMTS1, 4, 5, 8, 9, 15, 20), pro‐collagenases (cleaving the N‐terminal propeptides of type I, II and III procollagens: ADAMTS2, 3 and 14), von Willebrand factor protease (ADAMTS13), and fibrillin/fibronectin proteases (ADAMTS6, 7, 10, 12, 16, 17, 18 and 19) [129]. Several ADAMTSs are expressed by PCs [30, 41, 42, 43, 52, 76, 97, 98] (Table 1). ADAMTS1 is involved in neovascularisation through its ability to bind VEGF, thus inhibiting VEGFR activation [130, 131]. VEGF expression increases in PC monocultures under hypoxic conditions, but the effect on ADAMTS1 expression is not known [29]. ADAMTS1 also cleaves thrombospondin 1, an adhesive PC glycoprotein [30, 44, 46, 48] that mediates cell–cell and cell–ECM interactions, thus releasing anti‐angiogenic peptides [132]. ADAMTS1 anti‐angiogenic activity is therefore both dependent and independent on proteolytic activity. As mentioned above, other ADAMTS1 substrates are the nidogens [95, 96]. Human brain PCs also express ADAMTS7 [42], a promising therapeutic target in atherosclerosis and coronary artery disease [128]. ADAMTS7 cleaves latent TGF‐β binding protein (LTBP) 4 *in vitro* [133, 134, 135]. LTBP4 is a negative regulator of the TGF‐β signalling pathway and is co‐expressed with ADAMTS7 by PCs in the CNS [30, 41, 42].

The ADAMs (21 genes in human) share with the ADAMTSs a similar domain organisation including a prodomain, a metzincin metalloproteinase domain and a disintegrin‐like domain, but this is followed C‐terminally by a cysteine‐rich domain, an EGF‐like domain (except for ADAM10 and ADAM17), a type I transmembrane domain and a cytoplasmic tail (Fig. 4). ADAMs cleave the extracellular regions of multiple transmembrane proteins in a process called ectodomain shedding [136]. ADAM17 is an important target in cancer and other inflammatory conditions [137] and is expressed by PCs in both human and mice [40, 41, 42] (Table 1). Among its more than 80 substrates, some are also expressed by PCs, including tumour necrosis factor (TNF)‐α [29, 138], interleukin (IL)‐8 [29, 83, 139] and colony stimulating factor 1 [29, 30, 42, 52] (Table 1). ADAM17 catalyses the shedding of the mitogen and chemotactic heparin‐binding epidermal growth factor receptor (HB‐EGFR) from the EC membrane [140] and soluble HB‐EGF cooperates with PDGF‐β to promote PC recruitment [141].

The serine protease HTRA1 is expressed by brain PCs [29, 30, 42] (Table 1). One of HTRA1's substrates is LTBP1, another negative regulator of TGF‐β signalling [142]. Genetic deletion of *Htra1* in mice results in reduced TGF‐β signalling and mutations in *HTRA1* are associated with cerebral autosomal recessive arteriopathy with subcortical infarcts and leukoencephalopathy in humans [143].

Cathepsins mainly act intracellularly, in particular in the acidic environment of the lysosome, but also as extracellular proteases. In contrast with metzincins, which are all zinc glutamyl proteases, the cathepsins include members with proteolytic mechanisms based on serine, aspartic acid and cysteine residues [144]. PC‐expressed cathepsins include cathepsin A (serine protease) [42], B (cysteine protease) [41, 52], D (aspartic protease) [28, 42, 52, 76], K [42], L [41, 52] and S (cysteine proteases) [40, 51] (Table 1). Cathepsins B, L and S cleave BM components, while cathepsin K has a preference for fibrillar collagens in the IM [145].

#### Protease inhibitors

TIMPs are a family of 4 small proteins (TIMP‐1, ‐2, ‐3, and ‐4, molecular weight 20–22 kDa) with inhibitory activity against MMPs, ADAMs and ADAMTSs [146]. Although all TIMPs have been shown to be expressed by PCs of various origin, the majority of studies have reported expression of TIMP‐1 [30, 38, 46, 48, 52, 147], TIMP‐2 [42, 46, 139] and TIMP‐3 [28, 30, 40, 41, 43, 45, 48, 52, 97, 118, 148] (Table 1). The function of TIMPs is that of counteracting the degradation of IM and BM components orchestrated by the above mentioned metzincin subfamilies. The mechanism of inhibition involves chelation of the metzincin active site zinc by the N‐terminal TIMP cysteine residue. TIMP‐2 and TIMP‐3 have the broadest inhibitory activity, with TIMP‐2 inhibiting preferably MMPs, and TIMP‐3 inhibiting ADAMs and ADAMTSs [146]. TIMP‐3 is a high affinity inhibitor of the proteoglycanases ADAMTS1 [149], ADAMTS4 [150], ADAMTS5 [151], ADAMTS9 [152] and ADAM17 [153]. TIMP‐3 is peculiar in being normally bound to GAGs associated with ECM and cell surfaces [154]. In addition to its inhibitory activity, TIMP‐3 binds to VEGFR2, thus inhibiting VEGF‐mediated signalling and angiogenesis [155, 156]. TIMP‐4 is a potent inhibitor of ADAMTS7 [134].

Serpins are a vast family of serine protease inhibitors. In contrast to TIMPs, which are reversible metzincin inhibitors, serpins irreversibly bind to their target by exposing a reactive cleavable loop that upon cleavage traps the target protease in an inactive state [157]. Numerous serpins are expressed by PCs in a variety of contexts [28, 30, 41, 42, 45, 46, 48, 52, 76] (Table 1).

#### Secreted factors

Upon sprouting of the new vessel, ECs recruit PC by secreting growth factors and cytokines such as PDGF [30, 41, 76, 83, 116], endothelin‐1 [158], transforming growth factor (TGF)‐β [27, 30, 38, 41, 42, 116, 159] and heparin‐binding EGF‐like growth factor [158].

Other factors secreted by PCs include FGFs [29, 30, 48, 52, 83, 159], insulin‐like growth factor 1 [83, 147], VEGF [29, 30, 38, 40, 42, 83, 116, 147, 159], follistatin like 1 (FSTL1) [30, 45, 98], IL‐6 [29, 42, 98, 116], IL‐10 [138, 160], IL‐11 [98], chemokine (C‐C motif) ligand 2 (CCL2) [29, 30, 52, 98, 147] and CCL11 [161], chemokine (C‐X‐C motif) ligand 5 (CXCL5) and CXCL6 [29], artemin [29, 30], TNF‐α and TNF‐β [29, 138, 147], HGF [29, 42, 116, 139, 147, 162], glial cell derived neurotrophic factor [27, 29, 159], and angiopoietin‐1 (ANGPT1) [30, 42, 50, 76, 97, 147]. These cytokines directly interact with BM components such as fibronectin, laminins, collagen type IV and heparan sulfate proteoglycans such as perlecan [23]. The balance between vessel growth and regression is maintained by PC secretion of pro‐ and anti‐angiogenic factors such as VEGF and ANGPT1 [163]. EC‐secreted PDGF‐β induces PC migration towards the endothelium by binding to the cell surface tyrosine kinase receptor PDGFRβ on PCs [164, 165], while PC‐secreted VEGF‐α and ANGPT1 promote survival and maturation of ECs [166] (Fig. 1). PDGF is normally bound to heparan sulfate proteoglycans expressed by ECs rather than PCs, as PC‐specific abrogation of heparan sulfate synthesis by selective genetic deletion of the glycosyltransferase *Ext1* does not affect PC recruitment and signalling in the CNS [167]. ANGPTs are key players in vascular remodelling [168]. The ANGPT1/Tie‐2 system plays a key role in vascular stabilisation. PCs secrete ANGPT1, which binds to Tie‐2 receptors on ECs, thus activating Tie‐2 signalling to promote vascular maturation (ECs do not secrete ANGPT1) (Fig. 1) [147]. The effects of ANGPT1 are antagonised by ANGPT2, which is predominantly expressed by ECs [169]. TGF‐β induces fibrosis by increasing the expression of collagen type I, connective tissue growth factor and fibronectin [19]. PC‐secreted CXCL1 [29, 147] facilitates penetration of leukocytes into the blood vessels [170].

We refer to Gaceb and Paul [20] for a comprehensive review of PC‐secreted factors.

## Pathophysiological remodelling of the extracellular matrix by pericytes

### Proteolytic remodelling of the ECM by PCs during vascular development, angiogenesis and cancer

**Fig. 5:**
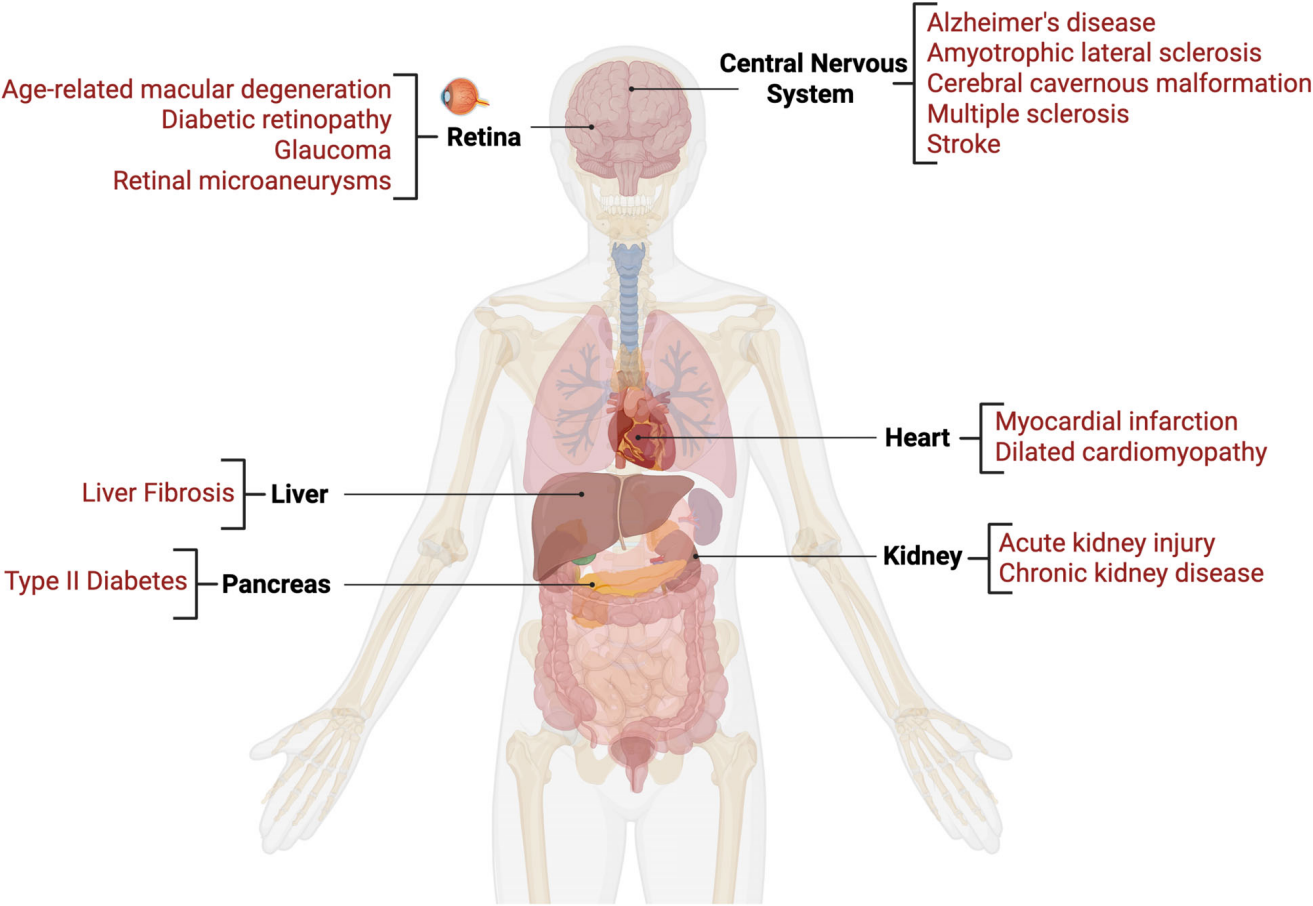
Involvement of pericytes in the dysregulated proteolytic remodelling of the extracellular matrix. The schematic shows a summary of pathologies characterised by dysregulated proteolytic remodelling of the extracellular matrix by pericytes in different tissues and organs. Figure created with BioRender.

In this section, we will describe general processes underlying ECM remodelling by PCs in the vasculature, while in the following sections we will focus on organ‐specific aspects (Fig. 5).

PCs play a crucial role in angiogenesis and vessel stability, demonstrated by the embryonic lethality of *Pdgfb* knockout mice, due to failure in PC recruitment and consequent lethal ruptured capillary microaneurysms [171], and the vascular anomalies (including cerebral endothelial hyperplasia, cardiac dilation and hypertrabeculation) observed in *Pdgfrb* knockout mice [164].

In order to produce angiogenic sprouting, ECs need to degrade the underlying BM before invading the surrounding perivascular stroma [172]. Concurrently, PCs become activated, detach from the BM and possibly participate in directional EC migration [173]. During this process, proteolytic remodelling of the ECM is orchestrated by the tightly regulated secretion of MMPs and TIMPs by both PCs and ECs. Once the angiogenic sprouts are formed, PC recruitment to the newly formed vessels ensures their stabilisation [174].

In an *in vitro* model of angiogenic sprouting, EC invasion into 3D collagen matrices and lumen morphogenesis was completely inhibited by TIMP‐2 and TIMP‐3 but not by TIMP‐1 [148]. Both TIMP‐3 and the broad spectrum MMP inhibitor GM6001/Ilomastat [175] inhibited the formation of embryonic vascular structures [148]. The source of TIMP‐3 is the PCs in the visceral endoderm and mesenchyme surrounding ECs, its function being that of blocking EC tube regression by inhibiting EC‐derived MMP1, MMP10, MT1‐MMP/MMP14 and ADAM15 [148]. Sprouting ECs also express TIMP‐1, TIMP‐2 and PAI‐1, which seem to play a minor role in regulating MMP‐dependent BM degradation and subsequent tube regression [148]. The TIMP‐3/MMP axis regulates BM remodelling in numerous vascular beds. Using a doxycycline‐inducible system to upregulate TIMP‐3 in human brain PCs, Yrigoin *et al*. found a marked narrowing of EC tube width, increased tube length and branching in a 3D collagen matrix PC/EC co‐culture model [11]. TIMP‐3 upregulation resulted in stabilisation and increased deposition of capillary BM components (collagen type IV, fibronectin and nidogen 2) [148], while TIMP‐3 knockdown in PCs decreased BM deposition [10]. PC‐secreted TIMP‐3 inhibits EC‐expressed MT1‐MMP/MMP14 and activation of pro‐MMP2 [118]. Conversely, blocking PC recruitment with pharmacological agents resulted in an increased tube width, decreased tube length and decreased BM deposition in 3D collagen matrices [148].

The microvascular BM represents a barrier to leucocyte transmigration (diapedesis) and metastatic invasion of cancer cells, therefore BM composition and degradation, in particular by MMPs, are key factors in these processes [12, 176] (Fig. 1). The source of these MMPs are PCs, ECs, ACs, macrophages and activated T cells [125, 177, 178]. MMPs are involved in tumour PC recruitment, migration, homing, proliferation and survival [179]. MMPs, in particular MMP9, contribute to PC recruitment by cleaving several ECM molecules, including fibronectin [180], and releasing angiogenic factors such as VEGF [181]. By stabilising the nascent microvessels around the tumour, the newly recruited PCs promote tumour survival and have been therefore proposed as a target for anti‐cancer therapy [179]. Infiltration of neutrophils occurs at regions where the BM is thin and not covered by PCs [182], indicating that the process is regulated by PC remodelling of the BM. Neutrophil recruitment is initiated by pro‐inflammatory activation of ECs, leading to EC upregulation of L‐selectins and intercellular adhesion molecule 1 (ICAM‐1) [183, 184]. IL1β induces PC deposition of fibronectin instead of collagen type I, with the former inducing EC‐expression of ICAM‐1 and positively correlating with neutrophil motility [49]. The interaction between EC‐presented integrin α5β1 and avβ3 and fibronectin induces EC survival and migration through activation of Src kinase and phosphorylation of VE‐cadherin that destabilise cell–cell junctions [185, 186].

The presence of NG2/CSPG4 is essential for maintaining PC‐ECs interactions and the assembly of the BM, as its specific deletion in murine PCs leads to decreased PC ensheathment of EC, diminished formation of endothelial junctions, and reduced BM assembly, overall decreasing vascularisation of intracranial melanomas [125, 177, 187]. In *Ng2* null mice, retinal and corneal angiogenesis is severely reduced compared to wild type littermates [188]. Tumour microvasculature in *Ng2* null mice has reduced PC coverage of EC, reduced BM assembly and increased hypoxia [177, 189, 190].

### PC remodelling of the ECM in the central nervous system

In the brain, PCs are located centrally between ECs, ACs and neurons where they exert important biological functions including promotion of angiogenesis, preservation of the BBB and regulation of capillary blood flow [23, 191]. Between 70% and 80% of CNS microvessels are covered by PCs, the highest PC density in the human body [192]. Collectively, ECs, PCs, ACs and neurons, that is, the vasculature and neural tissue, are defined as the neurovascular unit and PCs and ECs embedded in their BM form the BBB. PCs regulate the permeability of the BBB to water and solutes, mainly by regulating the expression of EC tight junction proteins including occludin, claudin 5 and zonula occludens‐1, gap junctions and peg‐and‐socket contacts [9, 50, 193]. Several solutes clear the BB specifically through active endothelial transcytosis which is negatively regulated by PC‐secreted vitronectin, as vitronectin binds to α5 integrin receptors on ECs to promote BBB integrity [84]. Mice globally lacking vitronectin or harbouring a vitronectin variant unable to bind α5 integrin exhibit BBB leakage [84]. Mice with genetic deletion of *Pdgfb* or its receptor *Pdgfrb* do not develop brain PCs [50, 119]. Their phenotype is characterised by compromised BBB integrity, increased vascular permeability, and cerebral microaneurysms due to defects in BM deposition [164, 165, 171, 194, 195, 196]. *Pdgfrb* null mice show a decrease in collagen type I and III as well as MMP9 in their BM [50]. Murine embryos lacking the TGFβ receptor activin receptor‐like kinase 5 (Alk5) in brain PCs (*Pdgfrb‐Cre, Alk5* ^*(flox/flox)*^) develop gross germinal matrix‐intraventricular haemorrhage by E12.5, resulting in perinatal lethality. In these mutants, germinal matrix microvessels, located ventral to the lateral ventricles, display abnormal dilation, reduced PC coverage, EC hyperproliferation, and increased PC levels of MMP2 and MMP9, resulting in increased gelatinolytic activity and reduced BM levels of collagen type I and IV. Furthermore, ALK5‐depleted PCs downregulate TIMP‐3. Conversely, TIMP‐3 administration improves endothelial morphogenesis and attenuates germinal matrix‐intraventricular haemorrhage [119].

Dysregulated PC activity is involved in several CNS diseases including Alzheimer's disease (AD), amyotrophic lateral sclerosis, multiple sclerosis and stroke [23] (Fig. 5). Brain microvascular dysfunction in AD includes impaired cerebral blood flow, ECM breakdown causing vascular fragility and degeneration, and altered BBB permeability. AD is characterised by brain accumulation of amyloid β, which forms plaques and fibrils, ultimately leading to neurodegeneration [197]. The AD ECM is distinguished by the presence of a thickened BM as well as an altered molecular composition caused by an imbalance between protein synthesis and proteolysis [198]. BM components such as laminins, nidogens and collagen type IV interfere with the formation of amyloid β fibrils (which induce PC apoptosis) [199], therefore their synthesis by PCs and other vascular cells may represent a response to amyloid β accumulation (reviewed in [198]). On the other hand, perlecan plays a detrimental role in AD by accelerating amyloid β fibril formation and stabilisation [200]. Accumulation of amyloid β impairs PC function by stimulating shedding of NG2/CPSG4 and PDGFRβ by MMP9/MMP2, which is also enhanced by hypoxia [201, 202]. PCs actively contrast amyloid β accumulation by clearance through low‐density lipoprotein receptor‐related protein 1 endocytosis [120, 203] and by secreting MMP2/MMP9 which degrade amyloid β [204, 205].

A recent sc‐RNAseq analysis of the brain vasculature from AD patients has identified two PC subclusters, the T‐PCs (enriched for small molecule transmembrane transport proteins) and M‐pericytes (enriched in matrisome proteins). The latter are particularly subjected to loss in AD, thus providing a possible mechanism behind the BBB breakdown observed in these patients [43]. Among the gene variants associated with an increased risk of AD in Genome‐wide association studies, the proteases ADAMTS1 and ADAMTS4 and the glycoprotein agrin are strongly expressed in PCs [43].

Fibrosis after injury of the CNS severely impairs neuronal regeneration and PCs are a main source of fibrotic ECM in this context, being the progenitors of stromal fibroblasts in fibrotic scar tissue forming after spinal cord injury [206, 207]. Upon injury, PCs proliferate, degrade the BM and migrate to mediate wound closure, a process that degenerates in fibrotic scar formation, with secretion of fibronectin and collagen I [207, 208]. Involvement of PCs in fibrotic scar formation has been experimentally demonstrated in CNS lesions caused by autoimmune encephalomyelitis [207]. scRNA‐seq of human brain‐derived PCs cultured alone or in the presence of ECs indicates that lack of paracrine communication or direct interaction with ECs moves PCs towards a profibrotic trajectory, with increased expression of collagens, fibronectin and laminins [42]. TGF‐β signalling does not drive ECM production in PCs, as PCs co‐cultured with ECs have higher TGF‐β activation than in monocultures, with elevated expression of the TGF‐β target PAI‐1 [42]. TGF‐β1 increases expression of both MMP2 and MMP9 [209, 210].

After neuronal demyelination, PCs express laminin α2 which induces oligodendrocyte differentiation, a process important in CNS repair and regeneration, in particular in multiple sclerosis [211, 212].

Stroke is a neurological damage caused by insufficient blood supply to the CNS and is classified into ischemic stroke, caused by vascular occlusion, and haemorrhagic stroke, caused by vascular rupture and subsequent bleeding [213]. PCs are among the cell types most quickly responding to hypoxic stress [214] and transcriptomic data pinpoint that their response involves a dramatic ECM remodelling [98]. In a permanent middle cerebral arterial occlusion mouse model, IL‐6, IL‐11, FSTL1 and ADAMTS4 are among the 10 top most upregulated genes in PCs 24 hours after stroke, while ADAMTS1 expression was upregulated already 1 h after stroke [98]. The coordinated expression of these matrisome genes suggests a switch to an anti‐inflammatory and repair response immediately after acute ischemic stroke. A scRNA‐seq analysis of brain PCs in a murine model of middle cerebral arterial occlusion also revealed specific upregulation of CCL11, a chemokine involved in eosinophile recruitment [161].

Inflammatory cytokines such as IL‐6 and interferon‐α upregulate expression of proteases in vascular beds, leading to BM degradation [198, 215]. Under ischemic conditions, brain PCs secrete cathepsin L, which degrades perlecan, resulting in the release of an anti‐apoptotic C‐terminal fragment of endorepellin [216]. PC‐secreted MMP2 and MMP9 [210, 217] are crucial mediators of BBB breakdown in both acute ischemic stroke and small‐vessel disease dementia [218, 219, 220]. In the early stage of ischemic stroke, MMPs contribute to acute neurovascular damage [221]. During capillary ischemia, PCs mobilise MMP9 at their soma. Secreted activated MMP9 can then cause BBB leakage [220] by localised degradation of tight junction proteins occludin, claudin‐5 and zonula occludens‐1 [218, 221] and BM components including collagen type IV [222], as well as mobilisation of VEGF [217]. This process was demonstrated in mice using *in vivo* two‐photon microscopy [220] and may be exacerbated by generation of superoxide by PC‐expressed adenine dinucleotide phosphate oxidase [120, 223, 224]. At the initial stages of ischemia, PCs start secreting MMP9 which rapidly degrades the BM, resulting in capillary leakage [220] and PC migration, which leads to PC loss [120]. Secretion of MMP9 by rat brain PCs is TNF‐α dependent and PCs are the major source of this gelatinase, compared to ECs or ACs, at least in monocultures [120], implicating PCs as major players in the remodelling of CNS BM under inflammatory conditions. In the post‐acute phase, upregulation of MMP2 and MMP9 occurs as part of a repair response aimed at ECM remodelling, thus underscoring a secondary beneficial role for gelatinases during stroke recovery [125].

PC loss is also a feature of cerebral cavernous malformation (CCM) [225] and mice harbouring endothelial deletion of KRIT1/CCM1 have exacerbated CCM lesions when administered an inhibitor of PDGFRβ. Such lesions were characterised by increased BM thickness with deposition of PC‐secreted fibronectin [44].

### PC remodelling of the ECM in the heart

PCs are the second most abundant cell type in the heart after ECs [226] and have recently been described as pleiotropic regulators of physiological heart function and central players in post‐injury response and regeneration; we refer to a recent comprehensive review of this topic [227]. Cardiac PCs contracts in response to adrenergic stimulation, changing luminal diameter by reorganising their cytoskeletal proteins and transmitting mechanical forces to the ECM [8, 228].

PC loss is involved in the increased microvascular permeability observed in myocardial infarction [40], as well as in several stages of cardiac repair [229] (Fig. 5). Vascular permeability is mediated by the effects of pro‐nerve growth factor, VEGF and ANGPT2 on PCs [230, 231, 232]. Conversely, PCs contribute to post‐infarction inflammation by secreting several cytokines [229] (Table 1).

After myocardial infarction, forming a stable scar is essential to prevent infarct expansion and rupture. Cardiac PCs contribute to scar formation by upregulating and secreting ECM proteins, including collagen I, largely through PDGF‐β/PDGFRβ signalling [233]. They also release high levels of SPARC under hypoxic or nutrient‐deprived conditions, which supports scar stabilisation and reduces rupture risk [99].

Some reports indicate that PCs may also differentiate into ECM‐secreting myofibroblasts, characterised by excessive secretion of pro‐angiogenic growth factors, fibrillar collagens, thrombospondins, proteases (MMP2 and MT1‐MMP/MMP14) and protease inhibitors (PAI‐1, TIMP‐2) [46], suggesting an unbalanced proteases/inhibitors ratio. Seven days after myocardial infarction, approximately 4% of myofibroblasts are of PC origin. These PC‐derived infarct fibroblasts exhibit higher expression of fibrillar collagens, periostin and thrombospondin 1, further contributing to cardiac fibrosis [46]. However, studies using nestin–NG2^+^ reporter mice suggest that not all PC subpopulations produce collagens, indicating a functional heterogeneity that could be targeted therapeutically [234]. PCs can also limit scar expansion by promoting neovascularisation in the border zone [235], a reparative process initiated by PC remodelling of the BM, mainly through MMP secretion [229].

ScRNA‐seq analysis identified notable transcriptional shifts in matrisome composition in PCs from patients with dilated cardiomyopathy compared to healthy donors, including higher levels of connective tissue growth factor and decreased levels of TIMP‐1 [52].

### PC remodelling of the ECM in the retina

The blood‐retinal barrier (BRB) is similar to the BBB whereby the microvascular integrity of retina is maintained by the interaction of PCs and ECs in a 1:1 ratio [236]. Many pathologies affecting the retina such as diabetic retinopathy (DR), glaucoma and age‐related macular degeneration see an involvement of PC dysfunction or altered PC/EC interactions [237] (Fig. 5).

In diabetes, chronic hyperglycaemia (elevated blood glucose levels) and metabolic dysregulation drive PCs towards a profibrotic ECM expression profile characterised by sustained upregulation of fibronectin, collagen type IV and tenascin C, particularly in the subendothelial matrix/BM. These proteins accumulate early in DR, even preceding overt vascular lesions, and are closely related to the breakdown of the BRB by disrupting PC‐EC interactions and α5β1‐Src‐VE‐cadherin signalling [238, 239]. Ultimately, this results in PC apoptosis, decreased PC coverage (PC:EC ratio 1:4), microvascular regression with reduced vessel density and length, and capillary abnormalities leading to microaneurysms [237, 240].

Retinal microaneurysms are capillary dilations generally associated with DR, hypertension, atherosclerosis, venous thrombosis, glaucoma, glioma and other retinal conditions [241, 242]. These microaneurysms are characterised by increased expression of specific BM components (collagen type IV, laminin, fibronectin, nidogens, perlecan), by both PCs and ECs [243]. Large microaneurysms show increased expression of MMP9, PAI‐1, LOXL2 and LOXL4 indicating dysregulated levels and crosslinking of collagens, in particular type III and IV. These matrisome changes associate with increased stiffness of the microvascular vessels and reduced PC coverage [243].

Secretion of fibronectin by bovine retinal PCs is higher than that of ECs and intensifies with increasing concentrations of glucose [244]. Upregulated expression of matrisome components is primarily mediated through protein kinase C‐dependent activation of α5β1 integrins, which promotes ECM accumulation independently of changes in gene expression. This is potentiated by non‐enzymatic glycation of ECM proteins, which enhances resistance to proteolysis and alters receptor binding affinity. Glycation of fibronectin and collagen type IV by reactive oxygen species creates advanced glycation end‐products (AGEs), which inhibit proteolysis by MMPs, thus reducing ECM turnover and promoting a feedback loop of further ECM accumulation [239]. AGEs also increase fibronectin expression in human brain PCs through TGF‐β signalling, while increasing MMP2 expression through VEGF‐mediated autocrine signalling in ECs. EC‐secreted MMP2 then targets tight junction proteins, thus causing a disruption of the BBB [38].

Crosslinking enzymes such as LOXLs and peroxidasin are also induced under high glucose conditions contributing to ECM stiffening and protease resistance. This enhances permanence of the fibrotic matrix and impairs mechanical compliance of the capillary wall, leading to increased vascular resistance and impaired PC‐EC crosstalk. In contrast, in regenerative and reparative contexts, there is a downregulation of fibrotic ECM gene expression and a reactivation of protease pathways. Restoration of ECM homeostasis is supported by reduced AGEs and normalisation of MMP2/MMP9 proteolytic activity, enabling breakdown of the fibrotic ECM and restoration of a functional BM [38].

Human retinal PCs increase secretion of VEGF under diabetic conditions (33 mM glucose, 2% O_2_), but this effect is suppressed when PCs are co‐cultured with ECs. Diabetic conditions also cause a reduction in secretion of HGF, TIMP‐2 and IL‐8, suggesting an angiogenic response associated with increased inflammation and an imbalance of the MMP/TIMP ratio leading to BM breakdown [139]. Increased ECM degradation is known to induce PC detachment and subsequent apoptosis [245]. Diabetic conditions (28 mM glucose) also increase MMP2 gelatinase activity in cultured human retinal PCs, while expression of TIMP‐1, TIMP‐2 and TIMP‐3 is unchanged [117]. Cytokines TNF‐α and IL‐1β, which play a key role in chronical retinal inflammation [246], induce expression of BM components collagen type IV, laminin β1, agrin and perlecan in human retinal PCs which enhance adhesion of ECs and peripheral blood mononuclear cells, thus further supporting the notion that PCs control leukocyte intravasation [47].

EC‐expressed Semaphorin 4D binds to Plexin B1 on PCs, thus inducing PC migration and EC VE‐cadherin dysfunction through the mDIA1‐Src pathway [104]. Knockdown of the gene coding for Plexin B1, *PLXNB1*, in PCs reverses the increase in permeability observed in PC/EC co‐cultures in the presence of recombinant semaphorin 4D. Adenovirus‐mediated knockdown of *PLXNB1 in* PCs decreased vascular leakage in a mouse model of streptozotocin‐induced retinopathy [104]. In patients with age‐related macular degeneration and in mouse models, smoking/exposure to smoke increases the levels of cell surface Semaphorin 4D, primarily on CD8^+^ T cells. Semaphorin 4D then interacts with Plexin B1 on retinal PCs to induce PC contraction, migration and ECM deposition [103].

### PC remodelling of the ECM in kidney

In kidney, PDGF‐β secreted by ECs in response to microvascular injury induces PC detachment, migration and differentiation into myofibroblasts, the cell type responsible for secreting a fibrotic ECM rich in collagen type I and III [247]. Kidney PCs stabilise capillary tube network formation in three‐dimensional gels, partly by inhibiting activation of EC‐derived MMP9 [97]. Mice subjected to unilateral ureteral obstruction, a model of kidney fibrosis, undergo proliferation and migration of kidney PCs, also called podocytes, which then differentiate into αSMA‐expressing myofibroblasts [3]. This process, which results in a reduced number of peritubular capillaries, decreased blood flow and glomerular filtration rate, is promoted by PC‐secreted VEGF‐A [3, 248, 249]. Destabilisation and loss of peritubular capillaries lead to kidney ischemia, a factor in the development of chronic kidney disease (CKD) and acute kidney injury [250, 251]. Kidney capillary permeability is regulated by fenestration of the endothelium; however, the glomerular BM exerts the primary filtration role by selectively preventing access of negatively charged macromolecules into the urinary space [252]. Recent scRNA‐seq and diffusion map analysis indicate that in CKD fibrotic myofibroblasts originate from both tubular PCs and fibroblasts, with PCs expressing periostin, osteoglycin and collagen type XIV during TGF‐β‐mediated differentiation [253].

In a mouse model of acute kidney injury, kidney PCs rapidly activate expression of ADAMTS1 and downregulate that of its inhibitor TIMP‐3. PC‐derived TIMP‐3 and ADAMTS1 work antagonistically in this model, with TIMP‐3 stabilising and ADAMTS1 destabilising the capillary tubular networks in the presence of kallikrein. *Timp3* knockout mice show a spontaneous microvascular phenotype in the kidney resulting from overactivated PCs and are more susceptible to acute ischemia reperfusion injury with exacerbated fibrosis [97].

### PC remodelling of the ECM in pancreas

PCs are present within and around pancreatic islets where they control insulin secretion by regulating blood flow in response to vasoactive stimuli transmitted by the neighbouring ECs, sympathetic nerves and the β‐cells responsible for insulin secretion [254, 255]. Even though they are also present in the exocrine pancreatic microvasculature, PCs have a significant higher coverage in the islets of Langerhans where they adjust capillary diameter by contraction and relaxation. Like glomerular capillaries, also the islet capillaries are fenestrated. When activated by high glucose levels, β‐cells induce PC relaxation by secreting adenosine, thus dilating islet capillaries and increasing local blood flow, while sympathetic neurotransmitters adrenaline and noradrenaline have the opposite effects [255].

As discussed in more detail in the Section ‘The pericyte matrisome: an Atlas’, bulk and scRNA‐seq data on murine and human pancreatic tissues show that PCs express both IM and BM components including fibrillar collagens, SLRPs, laminins and nidogens [39] and proteases such as ADAMTS1, ADAMTS4, ADAMTS9, tPA, uPA and cathepsin D [76] (Table 1). In addition, PCs secrete cytokines such as CXCL1, IL‐6 [256] and IL‐33 [257], growth factors such as bone morphogenetic protein 4 [258] and connective tissue growth factor [259], and BM components such as laminin 421 and collagen type IV [39] which contribute to β‐cell maturation. This PC matrisome composition should be maintained to preserve proper β‐cell function. In mice, PC loss causes β‐cell dedifferentiation and impaired insulin secretion in response to glucose [254, 260, 261]. PC loss is also observed in humans, where PC coverage around capillaries drops from 40% in healthy human islets to around 30% in type 2 diabetic donors [255]. Loss of pancreatic PCs, caused by their detachment from ECs, migration and apoptosis, is a hallmark of diabetic islet vasculopathy (reviewed in [260]).

### PC remodelling of the ECM in the liver

ECM accumulation is a landmark of liver fibrosis, a chronic liver injury caused by several factors including excessive alcohol consumption, viral infections and hepatitis [262]. Liver PCs, also known as hepatic stellate cells (HSCs), normally dormant in the liver, become activated in response to damage and inflammatory stimuli, differentiating into proliferative, fibrogenic myofibroblasts secreting fibrillar collagens, LOX, TIMPs and proteins associated with microfibrils such as microfibrillar‐associated protein 2 (MFAP‐2) and fibulin‐5 [263, 264, 265, 266]. Together with portal fibroblasts, activated HSCs are the major sources of fibrillar collagens and LOXs and are therefore responsible for the increased ECM stiffness observed in liver fibrosis [263]. Expression of LOX increases in rat HSCs 7 days after initiation of liver fibrosis induced by carbon tetrachloride (CCL_4_) treatment [263]. Administration of the cell permeable small molecule LOXL2 inhibitor PAT‐1251 to murine and rat HSCs significantly decreases cell proliferation as well as the expression of profibrogenic markers TGF‐β, collagen type I and TIMP‐1. Remarkably, PAT‐1251 decreases collagen deposition, hepatic fibrosis and injury in a mouse model of progressive biliary fibrosis [267].

Expression of MFAP‐2, a component of ECM microfibrils, is also increased in activated HSCs in human fibrotic livers and mouse models of liver fibrosis [264]. Studies in *Mfpa2* null mice subjected to CCL_4_ treatment established that MFAP‐2 facilitates regression of liver fibrosis and decreases intrahepatic inflammation by inhibiting macrophage infiltration, an effect mediated by induction of macrophage migration inhibitory factor expression [264]. Expression of fibulin‐5, a protein involved in the assembly of elastic fibres, also increases in activated HSCs. Plasma fibulin‐5 levels increase with fibrosis progression in patients with chronic hepatitis C and have been proposed as a diagnostic biomarker for advanced liver fibrosis in these patients [265]. In addition to its MMP inhibitory function, TIMP‐1 promotes HSC proliferation, further exacerbating liver fibrosis [268].

## Therapeutic approaches to target PC remodelling of the ECM

As discussed in the Section ‘Proteolytic remodelling of the ECM by PCs during vascular development, angiogenesis and cancer’, PCs are gatekeepers of capillary permeability, function and integrity. The interaction between ECs and PCs is a key element in the maintenance of a healthy vasculature, thus any perturbation of this delicate equilibrium may have pathological consequences (Figs 1 and 5). As both ECs and PCs are enmeshed in the BM, this specialised ECM is the medium through which these two cell types exert their functions and action their reciprocal interactive signals. The main challenges for targeting PCs in the microvasculature include the biological heterogeneity of PCs and their intricate interactions with ECs, the lack of appropriate delivery strategies specific for PCs and the lack of theragnostic biomarkers to be used as a readout of treatment effectiveness. Gaining a deeper understanding of PCs and their interaction with ECs will provide insights on disease mechanism and open up therapeutic options. More research is necessary to investigate the role of ECM in the PC/EC crosstalk.

Overall, there is a growing interest in developing strategies that may restore the PC/EC ratio in diseased organs (we refer to [227] for a review on PC supplementation therapies in the context of cardiovascular repair). So far, therapeutic targets have been mainly cell‐based with a focus on ECs [16], less on PC‐targeting therapies. These difficulties are exacerbated by the need for PC‐targeting drugs to cross the BBB or the BRB.

Blocking PDGFRβ, for example by antibodies, has been proposed as a strategy to treat tumoral angiogenesis [269]. On the other hand, recombinant PDGF‐β was tested in a phase I clinical trial (NCT00866502) for Parkinson's disease [270] as it may promote vascular stabilisation [271]. Drugs not specifically targeting PCs have been reported to restore PC function; for example, the administration of thalidomide in patients with hereditary haemorrhagic telangiectasia [272].

Faricimab (Vabysmo) is a bispecific monoclonal antibody targeting both VEGF‐A and ANGPT2 and approved for treatment of neovascular age‐related macular degeneration and diabetic macular oedema where it works by improving PC‐EC interactions and reducing PC loss, thus restoring microvascular integrity [273]. Anti‐semaphorin 4D antibodies have proven effective in reducing PC loss in DR and choroid neovascularisation models, alone or in combination with anti‐VEGF therapy, thus inhibiting retinal neovascularisation and vascular leakage [103, 104], but the intravitreal route of administration may limit their application in the clinic. The anti‐semaphorin 4D monoclonal antibody VX15/2503 (Pepinemab), which inhibits the interaction of Semaphorin 4D with Plexin B1 [274], has recently been tested in several phase I clinical trials for treatment of solid tumours (available at https://clinicaltrials.gov/), where it was well tolerated.

Restoring ECM integrity may be an indirect approach to preserve PC viability as BM disruption and thickening are both known to cause PC loss [120, 238, 245]. Another advantage of targeting ECM remodelling is that this approach does not need to be PC‐specific, as both ECs and PCs contribute to secretion of both BM and IM components [10]. MMPs, in particular the gelatinases MMP2 and MMP9, would be the preferred target, but research in MMP inhibitors has been plagued by repetitive failures, especially in early clinical trials [275]. This is in part due to high structural homology and the conserved catalytic zinc‐binding site present in MMPs, ADAMTSs and ADAMs [124]. Because the first metalloprotease inhibitors were based on zinc‐binding groups such as hydroxamates or carboxylates, they were poorly selective and exhibited off‐target effects. Currently, the only FDA‐approved MMP inhibitor is the tetracycline analogue doxycycline (Periostat®) for the treatment of periodontal disease [276]. However, increased protein structural information and computer aided drug design opened up ways to develop non‐hydroxamate/non‐zinc binding inhibitors [277]. Monoclonal antibodies have been investigated as MMP inhibitors because of their intrinsic higher selectivity compared to small molecules, but so far have failed to reach the bedside [278].

Some drugs indirectly act by downregulating gelatinase expression and may have therefore the additional advantage of preserving BM integrity. In stroke‐prone spontaneously hypertensive rats, the antiplatelet drug cilostazol prevented PC loss, induced PC proliferation and angiogenesis and inhibited expression of MMP9 [279]. Similar effects were observed with the free radical scavenger, edaravone, in a rat model of ischemia [280]. While MMPs have a detrimental effect in the acute phase of stroke by damaging the BBB through excessive BM degradation, in the post‐stroke phase their activity may promote tissue remodelling and repair [125], therefore, any treatment targeting gelatinase activity in stroke should be timely delivered.

In summary, strategies aimed at modulating PC remodelling of the ECM have the potential to restore vascular homeostasis but more research is needed to finely tune their selectivity, delivery and efficacy.

## Conclusion

PCs permeate all tissues and organs in the body and play a critical role in regulating vascular function through interaction with ECs and remodelling of the ECM. Their pleiotropic function in physiology, pathology and regeneration is intimately interconnected with their capacity to deposit and modify the extracellular milieu that forms the microvascular niche. Nevertheless, few studies have directed their attention to assessing and targeting this feature to re‐establish homeostasis or prevent vascular degeneration. With the new scientific insights, novel advanced technologies and improved understanding of the matrisome, we envision new avenues for understanding and treating diseases characterised by PC dysfunction manifesting through matrisome changes.

## Conflict of interest

The authors declare no conflict of interest.

## Author contributions

TB: conceptualisation; data curation, writing‐original draft, visualisation. EM: conceptualisation; data curation, writing‐original draft. KQ: data curation, writing‐original draft, visualisation. PC: conceptualisation, methodology, writing‐original draft, writing‐review & editing, supervision. SS: conceptualisation, writing‐original draft, writing‐review & editing, visualisation, supervision, project administration, funding acquisition.

## References

[1] van Dijk CG , Nieuweboer FE , Pei JY , Xu YJ , Burgisser P , van Mulligen E , el Azzouzi H , Duncker DJ , Verhaar MC & Cheng C (2015) The complex mural cell: pericyte function in health and disease. Int J Cardiol 190, 75–89. doi: 10.1016/j.ijcard.2015.03.258 25918055

[2] Armulik A , Genové G & Betsholtz C (2011) Pericytes: developmental, physiological, and pathological perspectives, problems, and promises. Dev Cell 21, 193–215. doi: 10.1016/j.devcel.2011.07.001 21839917

[3] Lin SL , Kisseleva T , Brenner DA & Duffield JS (2008) Pericytes and perivascular fibroblasts are the primary source of collagen‐producing cells in obstructive fibrosis of the kidney. Am J Pathol 173, 1617–1627. doi: 10.2353/ajpath.2008.080433 19008372 PMC2626374

[4] Harrell CR , Simovic Markovic B , Fellabaum C , Arsenijevic A , Djonov V & Volarevic V (2018) Molecular mechanisms underlying therapeutic potential of pericytes. J Biomed Sci 25, 21. doi: 10.1186/s12929-018-0423-7 29519245 PMC5844098

[5] Fujita T & Narumiya S (2016) Roles of hepatic stellate cells in liver inflammation: a new perspective. Inflamm Regen 36, 1. doi: 10.1186/s41232-016-0005-6 29259674 PMC5721720

[6] Kostallari E & Shah VH (2019) Pericytes in the liver. Adv Exp Med Biol 1122, 153–167. doi: 10.1007/978-3-030-11093-2_9 30937868 PMC7137998

[7] da Silva ML , Marson RF , Solari MIG & Nardi NB (2020) Are liver Pericytes just precursors of Myofibroblasts in hepatic diseases? Insights from the crosstalk between perivascular and inflammatory cells in liver injury and repair. Cells 9, 188. doi: 10.3390/cells9010188 31940814 PMC7017158

[8] Dessalles CA , Babataheri A & Barakat AI (2021) Pericyte mechanics and mechanobiology. J Cell Sci 134, jcs240226. doi: 10.1242/jcs.240226 33753399

[9] Armulik A , Genové G , Mäe M , Nisancioglu MH , Wallgard E , Niaudet C , He L , Norlin J , Lindblom P , Strittmatter K *et al*. (2010) Pericytes regulate the blood‐brain barrier. Nature 468, 557–561. doi: 10.1038/nature09522 20944627

[10] Stratman AN , Malotte KM , Mahan RD , Davis MJ & Davis GE (2009) Pericyte recruitment during vasculogenic tube assembly stimulates endothelial basement membrane matrix formation. Blood 114, 5091–5101. doi: 10.1182/blood-2009-05-222364 19822899 PMC2788982

[11] Yrigoin K , Bernard KN , Castaño MA , Cleaver O , Sumanas S & Davis GE (2024) Enhancing human capillary tube network assembly and maturation through upregulated expression of pericyte‐derived TIMP‐3. Front Cell Dev Biol 12, 1465806. doi: 10.3389/fcell.2024.1465806 39544367 PMC11560913

[12] Yurchenco PD (2011) Basement membranes: cell scaffoldings and signaling platforms. Cold Spring Harb Perspect Biol 3, a004911. doi: 10.1101/cshperspect.a004911 21421915 PMC3039528

[13] Naba A (2024) Mechanisms of assembly and remodelling of the extracellular matrix. Nat Rev Mol Cell Biol 25, 865–885. doi: 10.1038/s41580-024-00767-3 39223427 PMC11931590

[14] Garde A & Sherwood DR (2021) Fueling cell invasion through extracellular matrix. Trends Cell Biol 31, 445–456. doi: 10.1016/j.tcb.2021.01.006 33549396 PMC8122022

[15] Sekiguchi R & Yamada KM (2018) Basement membranes in development and disease. Curr Top Dev Biol 130, 143–191. doi: 10.1016/bs.ctdb.2018.02.005 29853176 PMC6701859

[16] Li G , Gao J , Ding P & Gao Y (2025) The role of endothelial cell‐pericyte interactions in vascularization and diseases. J Adv Res 67, 269–288. doi: 10.1016/j.jare.2024.01.016 38246244 PMC11725166

[17] Majesky MW , Dong XR , Hoglund V , Mahoney WM Jr & Daum G (2011) The adventitia: a dynamic interface containing resident progenitor cells. Arterioscler Thromb Vasc Biol 31, 1530–1539. doi: 10.1161/ATVBAHA.110.221549 21677296 PMC3382115

[18] Etchevers HC , Vincent C , Le Douarin NM & Couly GF (2001) The cephalic neural crest provides pericytes and smooth muscle cells to all blood vessels of the face and forebrain. Development 128, 1059–1068. doi: 10.1242/dev.128.7.1059 11245571

[19] Yamazaki T & Mukouyama YS (2018) Tissue specific origin, development, and pathological perspectives of Pericytes. Front Cardiovasc Med 27, 78. doi: 10.3389/fcvm.2018.00078 PMC6030356 29998128

[20] Humphreys BD , Lin SL , Kobayashi A , Hudson TE , Nowlin BT , Bonventre JV , Valerius MT , McMahon AP & Duffield JS (2010) Fate tracing reveals the pericyte and not epithelial origin of myofibroblasts in kidney fibrosis. Am J Pathol 176, 85–97. doi: 10.2353/ajpath.2010.090517 20008127 PMC2797872

[21] Hung C , Linn G , Chow YH , Kobayashi A , Mittelsteadt K , Altemeier WA , Gharib SA , Schnapp LM & Duffield JS (2013) Role of lung pericytes and resident fibroblasts in the pathogenesis of pulmonary fibrosis. Am J Respir Crit Care Med 188, 820–830. doi: 10.1164/rccm.201212-2297OC 23924232 PMC3826269

[22] Gaceb A & Paul G (2018) Pericyte Secretome. Adv Exp Med Biol 1109, 139–163. doi: 10.1007/978-3-030-02601-1_11 30523595

[23] Fu J , Liang H , Yuan P , Wei Z & Zhong P (2023) Brain pericyte biology: from physiopathological mechanisms to potential therapeutic applications in ischemic stroke. Front Cell Neurosci 14, 1267785. doi: 10.3389/fncel.2023.1267785 PMC10536258 37780206

[24] Khan NS , West CC , Rossi F & Crisan M (2021) Assessment of Pericyte phenotype by flow cytometry. Methods Mol Biol 2235, 27–35. doi: 10.1007/978-1-0716-1056-5_3 33576968

[25] West CC , Khan NS & Crisan M (2021) Characterization of human Pericyte phenotype by immunohistochemistry. Methods Mol Biol 2235, 37–45. doi: 10.1007/978-1-0716-1056-5_4 33576969

[26] Betsholtz C (2022) Toward a granular molecular‐anatomic map of the blood vasculature – single‐cell RNA sequencing makes the leap. Ups J Med Sci 21, 127. doi: 10.48101/ujms.v127.9051 PMC9602202 36337278

[27] Menaceur C , Hachani J , Dib S , Duban‐Deweer S , Karamanos Y , Shimizu F , Kanda T , Gosselet F , Fenart L & Saint‐Pol J (2023) Highlighting in vitro the role of brain‐like endothelial cells on the maturation and metabolism of brain Pericytes by SWATH proteomics. Cells 12, 1010. 37048083 10.3390/cells12071010 PMC10093307

[28] Wei W , Riley NM , Yang AC , Kim JT , Terrell SM , Li VL , Garcia‐Contreras M , Bertozzi CR & Long JZ (2021) Cell type‐selective secretome profiling in vivo. Nat Chem Biol 17, 326–334. doi: 10.1038/s41589-020-00698-y 33199915 PMC7904581

[29] Enström A , Carlsson R , Buizza C , Lewi M & Paul G (2024) Pericyte‐specific Secretome profiling in hypoxia using TurboID in a multicellular in vitro spheroid model. Mol Cell Proteomics 23, 100782. doi: 10.1016/j.mcpro.2024.100782 38705386 PMC11176767

[30] Naderi‐Meshkin H , Wahyu Setyaningsih WA , Yacoub A , Carney G , Cornelius VA , Nelson CA , Kelaini S , Donaghy C , Dunne PD , Amirkhah R *et al*. (2024) Unveiling impaired vascular function and cellular heterogeneity in diabetic donor‐derived vascular organoids. Stem Cells 42, 791–808. doi: 10.1093/stmcls/sxae043 39049437 PMC11384901

[31] Naba A , Clauser KR , Hoersch S , Liu H , Carr SA & Hynes RO (2012) The matrisome: in silico definition and in vivo characterization by proteomics of normal and tumor extracellular matrices. Mol Cell Proteomics 11, M111.014647. doi: 10.1074/mcp.M111.014647 PMC3322572 22159717

[32] Ricard‐Blum S (2011) The collagen family. Cold Spring Harb Perspect Biol 3, a004978. doi: 10.1101/cshperspect.a004978 21421911 PMC3003457

[33] Van Agtmael T & Bruckner‐Tuderman L (2010) Basement membranes and human disease. Cell Tissue Res 339, 167–188. doi: 10.1007/s00441-009-0866-y 19756754

[34] Jariwala N , Ozols M , Bell M , Bradley E , Gilmore A , Debelle L & Sherratt MJ (2022) Matrikines as mediators of tissue remodelling. Adv Drug Deliv Rev 185, 114240. doi: 10.1016/j.addr.2022.114240 35378216

[35] Hamano Y , Zeisberg M , Sugimoto H , Lively JC , Maeshima Y , Yang C , Hynes RO , Werb Z , Sudhakar A & Kalluri R (2003) Physiological levels of tumstatin, a fragment of collagen IV alpha3 chain, are generated by MMP‐9 proteolysis and suppress angiogenesis via alphaV beta3 integrin. Cancer Cell 3, 589–601. doi: 10.1016/s1535-6108(03)00133-8 12842087 PMC2775452

[36] Brassart‐Pasco S , Sénéchal K , Thevenard J , Ramont L , Devy J , Di Stefano L , Dupont‐Deshorgue A , Brézillon S , Feru J , Jazeron JF *et al*. (2012) Tetrastatin, the NC1 domain of the α4(IV) collagen chain: a novel potent anti‐tumor matrikine. PLoS One 7, e29587. doi: 10.1371/journal.pone.0029587 22539938 PMC3335157

[37] Mundel TM & Kalluri R (2007) Type IV collagen‐derived angiogenesis inhibitors. Microvasc Res 74, 85–89. doi: 10.1016/j.mvr.2007.05.005 17602710 PMC3998721

[38] Shimizu F , Sano Y , Tominaga O , Maeda T , Abe MA & Kanda T (2013) Advanced glycation end‐products disrupt the blood‐brain barrier by stimulating the release of transforming growth factor‐β by pericytes and vascular endothelial growth factor and matrix metalloproteinase‐2 by endothelial cells in vitro. Neurobiol Aging 34, 1902–1912. doi: 10.1016/j.neurobiolaging.2013.01.012 23428182

[39] Sakhneny L , Epshtein A & Landsman L (2021) Pericytes contribute to the islet basement membranes to promote beta‐cell gene expression. Sci Rep 11, 2378. doi: 10.1038/s41598-021-81774-8 33504882 PMC7840750

[40] Quijada P , Park S , Zhao P , Kolluri KS , Wong D , Shih KD , Fang K , Pezhouman A , Wang L , Daraei A *et al*. (2023) Cardiac pericytes mediate the remodeling response to myocardial infarction. J Clin Invest 133, e162188. doi: 10.1172/JCI162188 37183820 PMC10178847

[41] Vanlandewijck M , He L , Mäe MA , Andrae J , Ando K , Del Gaudio F , Nahar K , Lebouvier T , Laviña B , Gouveia L *et al*. (2018) A molecular atlas of cell types and zonation in the brain vasculature. Nature 554, 475–480. doi: 10.1038/nature25739 29443965

[42] Brandt MM , van Dijk CGM , Maringanti R , Chrifi I , Kramann R , Verhaar MC , Duncker DJ , Mokry M & Cheng C (2019) Transcriptome analysis reveals microvascular endothelial cell‐dependent pericyte differentiation. Sci Rep 9, 15586. doi: 10.1038/s41598-019-51838-x 31666598 PMC6821775

[43] Yang AC , Vest RT , Kern F , Lee DP , Agam M , Maat CA , Losada PM , Chen MB , Schaum N , Khoury N *et al*. (2022) A human brain vascular atlas reveals diverse mediators of Alzheimer's risk. Nature 603, 885–892. doi: 10.1038/s41586-021-04369-3 35165441 PMC9635042

[44] Dai Z , Li J , Li Y , Wang R , Yan H , Xiong Z , Wu S , Yang X , Lu D , Zhang D *et al*. (2022) Role of pericytes in the development of cerebral cavernous malformations. iScience 25, 105642. doi: 10.1016/j.isci.2022.105642 36465134 PMC9713377

[45] La Manno G , Siletti K , Furlan A , Gyllborg D , Vinsland E , Mossi Albiach A , Mattsson Langseth C , Khven I , Lederer AR , Dratva LM *et al*. (2021) Molecular architecture of the developing mouse brain. Nature 596, 92–96. doi: 10.1038/s41586-021-03775-x 34321664

[46] Alex L , Tuleta I , Hernandez SC , Hanna A , Venugopal H , Astorkia M , Humeres C , Kubota A , Su K , Zheng D *et al*. (2023) Cardiac Pericytes acquire a Fibrogenic phenotype and contribute to vascular maturation after myocardial infarction. Circulation 148, 882–898. doi: 10.1161/CIRCULATIONAHA.123.064155 37350296 PMC10527624

[47] Giblin MJ , Ontko CD & Penn JS (2022) Effect of cytokine‐induced alterations in extracellular matrix composition on diabetic retinopathy‐relevant endothelial cell behaviors. Sci Rep 12, 12955. doi: 10.1038/s41598-022-12683-7 35902594 PMC9334268

[48] Brown LA , Sava P & Garcia C (2015) Gonzalez AL proteomic analysis of the Pericyte derived extracellular matrix. Cel Mol Bioeng 8, 349–363. doi: 10.1007/s12195-015-0408-5

[49] Sava P , Cook IO , Mahal RS & Gonzalez AL (2015) Human microvascular pericyte basement membrane remodeling regulates neutrophil recruitment. Microcirculation 22, 54–67. doi: 10.1111/micc.12173 25214363

[50] Daneman R , Zhou L , Kebede AA & Barres BA (2010) Pericytes are required for blood‐brain barrier integrity during embryogenesis. Nature 468, 562–566. doi: 10.1038/nature09513 20944625 PMC3241506

[51] Xia M , Jiao L , Wang XH , Tong M , Yao MD , Li XM , Yao J , Li D , Zhao PQ & Yan B (2023) Single‐cell RNA sequencing reveals a unique pericyte type associated with capillary dysfunction. Theranostics 13, 2515–2530. doi: 10.7150/thno.83532 37215579 PMC10196835

[52] Koenig AL , Shchukina I , Amrute J , Andhey PS , Zaitsev K , Lai L , Bajpai G , Bredemeyer A , Smith G , Jones C *et al*. (2022) Single‐cell transcriptomics reveals cell‐type‐specific diversification in human heart failure. Nat Cardiovasc Res 1, 263–280. doi: 10.1038/s44161-022-00028-6 35959412 PMC9364913

[53] Murphy G & Nagase H (2008) Progress in matrix metalloproteinase research. Mol Aspects Med 29, 290–308. doi: 10.1016/j.mam.2008.05.002 18619669 PMC2810947

[54] Iozzo RV & Schaefer L (2015) Proteoglycan form and function: a comprehensive nomenclature of proteoglycans. Matrix Biol 42, 11–55. doi: 10.1016/j.matbio.2015.02.003 25701227 PMC4859157

[55] Ozerdem U , Monosov E & Stallcup WB (2002) NG2 proteoglycan expression by pericytes in pathological microvasculature. Microvasc Res 63, 129–134. doi: 10.1006/mvre.2001.2376 11749079

[56] Price MA , Colvin Wanshura LE , Yang J , Carlson J , Xiang B , Li G , Ferrone S , Dudek AZ , Turley EA & McCarthy JB (2011) CSPG4, a potential therapeutic target, facilitates malignant progression of melanoma. Pigment Cell Melanoma Res 24, 1148–1157. doi: 10.1111/j.1755-148X.2011.00929.x 22004131 PMC3426219

[57] Iida J , Wilhelmson KL , Ng J , Lee P , Morrison C , Tam E , Overall CM & McCarthy JB (2007) Cell surface chondroitin sulfate glycosaminoglycan in melanoma: role in the activation of pro‐MMP‐2 (pro‐gelatinase a). Biochem J 403, 553–563. doi: 10.1042/BJ20061176 17217338 PMC1876388

[58] Stallcup WB (2018) The NG2 proteoglycan in Pericyte biology. Adv Exp Med Biol 1109, 5–19. doi: 10.1007/978-3-030-02601-1_2 30523586

[59] Goretzki L , Burg MA , Grako KA & Stallcup WB (1999) High‐affinity binding of basic fibroblast growth factor and platelet‐derived growth factor‐AA to the core protein of the NG2 proteoglycan. J Biol Chem 274, 16831–16837. doi: 10.1074/jbc.274.24.16831 10358027

[60] Cattaruzza S , Ozerdem U , Denzel M , Ranscht B , Bulian P , Cavallaro U , Zanocco D , Colombatti A , Stallcup WB & Perris R (2013) Multivalent proteoglycan modulation of FGF mitogenic responses in perivascular cells. Angiogenesis 16, 309–327. doi: 10.1007/s10456-012-9316-7 23124902 PMC3656602

[61] Fukushi J , Makagiansar IT & Stallcup WB (2004) NG2 proteoglycan promotes endothelial cell motility and angiogenesis via engagement of galectin‐3 and alpha3beta1 integrin. Mol Biol Cell 15, 3580–3590. doi: 10.1091/mbc.e04-03-0236 15181153 PMC491820

[62] Chekenya M , Krakstad C , Svendsen A , Netland IA , Staalesen V , Tysnes BB , Selheim F , Wang J , Sakariassen PØ , Sandal T *et al*. (2008) The progenitor cell marker NG2/MPG promotes chemoresistance by activation of integrin‐dependent PI3K/Akt signaling. Oncogene 27, 5182–5194. doi: 10.1038/onc.2008.157 18469852 PMC2832310

[63] Lin XH , Dahlin‐Huppe K & Stallcup WB (1996) Interaction of the NG2 proteoglycan with the actin cytoskeleton. J Cell Biochem 63, 463–477. 8978462 10.1002/(sici)1097-4644(19961215)63:4<463::aid-jcb8>3.0.co;2-r

[64] Fang X , Burg MA , Barritt D , Dahlin‐Huppe K , Nishiyama A & Stallcup WB (1999) Cytoskeletal reorganization induced by engagement of the NG2 proteoglycan leads to cell spreading and migration. Mol Biol Cell 10, 3373–3387. doi: 10.1091/mbc.10.10.3373 10512873 PMC25605

[65] Ozerdem U , Grako KA , Dahlin‐Huppe K , Monosov E & Stallcup WB (2001) NG2 proteoglycan is expressed exclusively by mural cells during vascular morphogenesis. Dev Dyn 222, 218–227. doi: 10.1002/dvdy.1200 11668599

[66] Cao Z , Minnier J , Liu L , Scott KLL , Reddy AP , Wilmarth PA , David LL , Barnes AP , Grafe MR , Kaul S *et al*. (2022) Proteomic profiling of concurrently isolated primary microvascular endothelial cells, pericytes, and vascular smooth muscle cells from adult mouse heart. Sci Rep 12, 8835. doi: 10.1038/s41598-022-12749-6 35614104 PMC9132906

[67] Fears CY , Gladson CL & Woods A (2006) Syndecan‐2 is expressed in the microvasculature of gliomas and regulates angiogenic processes in microvascular endothelial cells. J Biol Chem 281, 14533–14536. doi: 10.1074/jbc.C600075200 16574663

[68] Farrugia BL & Melrose J (2023) The glycosaminoglycan side chains and modular Core proteins of Heparan sulphate proteoglycans and the varied ways they provide tissue protection by regulating physiological processes and cellular behaviour. Int J Mol Sci 24, 14101. doi: 10.3390/ijms241814101 37762403 PMC10531531

[69] Timpl R & Brown JC (1996) Supramolecular assembly of basement membranes. Bioessays 18, 123–132. doi: 10.1002/bies.950180208 8851045

[70] Costell M , Gustafsson E , Aszódi A , Mörgelin M , Bloch W , Hunziker E , Addicks K , Timpl R & Fässler R (1999) Perlecan maintains the integrity of cartilage and some basement membranes. J Cell Biol 147, 1109–1122. doi: 10.1083/jcb.147.5.1109 10579729 PMC2169352

[71] Solomonov I , Kollet O & Sagi I (2025) Extracellular matrix and proteolysis: mechanisms driving irreversible changes and shaping cell behavior. FEBS J 293, 3788–3813. doi: 10.1111/febs.70292 41178498 PMC13326534

[72] Goyal A , Pal N , Concannon M , Paul M , Doran M , Poluzzi C , Sekiguchi K , Whitelock JM , Neill T & Iozzo RV (2011) Endorepellin, the angiostatic module of perlecan, interacts with both the α2β1 integrin and vascular endothelial growth factor receptor 2 (VEGFR2): a dual receptor antagonism. J Biol Chem 286, 25947–25962. doi: 10.1074/jbc.M111.243626 21596751 PMC3138248

[73] Nyström A , Shaik ZP , Gullberg D , Krieg T , Eckes B , Zent R , Pozzi A & Iozzo RV (2009) Role of tyrosine phosphatase SHP‐1 in the mechanism of endorepellin angiostatic activity. Blood 114, 4897–4906. doi: 10.1182/blood-2009-02-207134 19789387 PMC2786295

[74] Kemberi M , Minns AF & Santamaria S (2024) Soluble proteoglycans and proteoglycan fragments as biomarkers of pathological extracellular matrix Remodeling. Proteoglycan Res 2, e70011. doi: 10.1002/pgr2.70011 39600538 PMC11587194

[75] Muragaki Y , Timmons S , Griffith CM , Oh SP , Fadel B , Quertermous T & Olsen BR (1995) Mouse Col18a1 is expressed in a tissue‐specific manner as three alternative variants and is localized in basement membrane zones. Proc Natl Acad Sci USA 92, 8763–8767. doi: 10.1073/pnas.92.19.8763 7568013 PMC41047

[76] Craig‐Schapiro R , Li G , Chen K , Gomez‐Salinero JM , Nachman R , Kopacz A , Schreiner R , Chen X , Zhou Q , Rafii S *et al*. (2025) Single‐cell atlas of human pancreatic islet and acinar endothelial cells in health and diabetes. Nat Commun 16, 1338. doi: 10.1038/s41467-024-55415-3 39915484 PMC11802906

[77] Moulton KS , Olsen BR , Sonn S , Fukai N , Zurakowski D & Zeng X (2004) Loss of collagen XVIII enhances neovascularization and vascular permeability in atherosclerosis. Circulation 110, 1330–1336. doi: 10.1161/01.CIR.0000140720.79015.3C 15313955

[78] Iozzo RV (2005) Basement membrane proteoglycans: from cellar to ceiling. Nat Rev Mol Cell Biol 6, 646–656. doi: 10.1038/nrm1702 16064139

[79] Ezura Y , Chakravarti S , Oldberg A , Chervoneva I & Birk DE (2000) Differential expression of lumican and fibromodulin regulate collagen fibrillogenesis in developing mouse tendons. J Cell Biol 151, 779–788. doi: 10.1083/jcb.151.4.779 11076963 PMC2169450

[80] Kashiwagi M , Enghild JJ , Gendron C , Hughes C , Caterson B , Itoh Y & Nagase H (2004) Altered proteolytic activities of ADAMTS‐4 expressed by C‐terminal processing. J Biol Chem 279, 10109–10119. doi: 10.1074/jbc.M312123200 Erratum in: J Biol Chem. 2004;279(21):22786. 14662755

[81] Tasheva ES , Koester A , Paulsen AQ , Garrett AS , Boyle DL , Davidson HJ , Song M , Fox N & Conrad GW (2002) Mimecan/osteoglycin‐deficient mice have collagen fibril abnormalities. Mol Vis 31, 407–415. 12432342

[82] Ge G , Seo NS , Liang X , Hopkins DR , Höök M & Greenspan DS (2004) Bone morphogenetic protein‐1/tolloid‐related metalloproteinases process osteoglycin and enhance its ability to regulate collagen fibrillogenesis. J Biol Chem 279, 41626–41633. doi: 10.1074/jbc.M406630200 15292192

[83] Bagley RG , Weber W , Rouleau C & Teicher BA (2005) Pericytes and endothelial precursor cells: cellular interactions and contributions to malignancy. Cancer Res 65, 9741–9750. doi: 10.1158/0008-5472.CAN-04-4337 16266995

[84] Ayloo S , Lazo CG , Sun S , Zhang W , Cui B & Gu C (2022) Pericyte‐to‐endothelial cell signaling via vitronectin‐integrin regulates blood‐CNS barrier. Neuron 110, 1641–1655. doi: 10.1016/j.neuron.2022.02.017 35294899 PMC9119930

[85] Rajendran S , Narayansamy A , Annamalai R , Cruze LD , Kathiresan P & Kuppan K (2025) Proteome of pericytes from retinal vasculature of diabetic donor eyes. Exp Eye Res 251, 110178. doi: 10.1016/j.exer.2024.110178 39580044

[86] Tigges U , Boroujerdi A , Welser‐Alves JV & Milner R (2013) TNF‐α promotes cerebral pericyte remodeling in vitro, via a switch from α1 to α2 integrins. J Neuroinflammation 1, 33. doi: 10.1186/1742-2094-10-33 PMC3616978 23448258

[87] Schvartz I , Seger D & Shaltiel S (1999) Vitronectin. Int J Biochem Cell Biol 31, 539–544. doi: 10.1016/s1357-2725(99)00005-9 10399314

[88] Hohenester E & Yurchenco PD (2013) Laminins in basement membrane assembly. Cell Adh Migr 7, 56–63. doi: 10.4161/cam.21831 23076216 PMC3544787

[89] Hallmann R , Horn N , Selg M , Wendler O , Pausch F & Sorokin LM (2005) Expression and function of laminins in the embryonic and mature vasculature. Physiol Rev 85, 979–1000. doi: 10.1152/physrev.00014.2004 15987800

[90] Gautam J , Zhang X & Yao Y (2016) The role of pericytic laminin in blood brain barrier integrity maintenance. Sci Rep 3, 36450. doi: 10.1038/srep36450 PMC5093438 27808256

[91] Di Russo J , Luik AL , Yousif L , Budny S , Oberleithner H , Hofschröer V , Klingauf J , van Bavel E , Bakker EN , Hellstrand P *et al*. (2017) Endothelial basement membrane laminin 511 is essential for shear stress response. EMBO J 36, 183–201. doi: 10.15252/embj.201694756 27940654 PMC5239996

[92] Bader BL , Smyth N , Nedbal S , Miosge N , Baranowsky A , Mokkapati S , Murshed M & Nischt R (2005) Compound genetic ablation of nidogen 1 and 2 causes basement membrane defects and perinatal lethality in mice. Mol Cell Biol 25, 6846–6856. doi: 10.1128/MCB.25.15.6846-6856.2005 16024816 PMC1190363

[93] Sasaki T , Göhring W , Pan TC , Chu ML & Timpl R (1995) Binding of mouse and human fibulin‐2 to extracellular matrix ligands. J Mol Biol 254, 892–899. doi: 10.1006/jmbi.1995.0664 7500359

[94] Hopf M , Göhring W , Kohfeldt E , Yamada Y & Timpl R (1999) Recombinant domain IV of perlecan binds to nidogens, laminin‐nidogen complex, fibronectin, fibulin‐2 and heparin. Eur J Biochem 259, 917–925. doi: 10.1046/j.1432-1327.1999.00127.x 10092882

[95] Canals F , Colomé N , Ferrer C , Plaza‐Calonge Mdel C & Rodríguez‐Manzaneque JC (2006) Identification of substrates of the extracellular protease ADAMTS1 by DIGE proteomic analysis. Proteomics 6 (Suppl 1), S28–S35. doi: 10.1002/pmic.200500446 16511810

[96] Martino‐Echarri E , Fernández‐Rodríguez R , Rodríguez‐Baena FJ , Barrientos‐Durán A , Torres‐Collado AX , Plaza‐Calonge Mdel C , Amador‐Cubero S , Cortés J , Reynolds LE , Hodivala‐Dilke KM *et al*. (2013) Contribution of ADAMTS1 as a tumor suppressor gene in human breast carcinoma. Linking its tumor inhibitory properties to its proteolytic activity on nidogen‐1 and nidogen‐2. Int J Cancer 133, 2315–2324. doi: 10.1002/ijc.28271 23681936

[97] Schrimpf C , Xin C , Campanholle G , Gill SE , Stallcup W , Lin SL , Davis GE , Gharib SA , Humphreys BD & Duffield JS (2012) Pericyte TIMP3 and ADAMTS1 modulate vascular stability after kidney injury. J Am Soc Nephrol 23, 868–883. doi: 10.1681/ASN.2011080851 22383695 PMC3338296

[98] Buizza C , Enström A , Carlsson R & Paul G (2024) The transcriptional landscape of Pericytes in acute ischemic stroke. Transl Stroke Res 15, 714–728. doi: 10.1007/s12975-023-01169-x 37378751 PMC11226519

[99] Avolio E , Mangialardi G , Slater SC , Alvino VV , Gu Y , Cathery W , Beltrami AP , Katare R , Heesom K , Caputo M *et al*. (2021) Secreted protein acidic and cysteine rich Matricellular protein is enriched in the bioactive fraction of the human vascular Pericyte Secretome. Antioxid Redox Signal 34, 1151–1164. doi: 10.1089/ars.2019.7969 33226850

[100] Awwad K , Hu J , Shi L , Mangels N , Abdel Malik R , Zippel N , Fisslthaler B , Eble JA , Pfeilschifter J , Popp R *et al*. (2015) Role of secreted modular calcium‐binding protein 1 (SMOC1) in transforming growth factor β signalling and angiogenesis. Cardiovasc Res 106, 284–294. doi: 10.1093/cvr/cvv098 25750188

[101] Gao X , Ye J , Huang X , Huang S , Luo W , Zeng D , Li S , Tang M , Mai R , Li Y *et al*. (2024) Research progress of the netrins and their receptors in cancer. J Cell Mol Med 28, e18241. doi: 10.1111/jcmm.18241 38546656 PMC10977403

[102] Reuten R , Zendehroud S , Nicolau M , Fleischhauer L , Laitala A , Kiderlen S , Nikodemus D , Wullkopf L , Nielsen SR , McNeilly S *et al*. (2021) Basement membrane stiffness determines metastases formation. Nat Mater 20, 892–903. doi: 10.1038/s41563-020-00894-0 33495631

[103] He K , Dong X , Yang T , Li Z , Liu Y , He J , Wu M , Wei‐Zhang S , Kaysar P , Cui B *et al*. (2025) Smoking aggravates neovascular age‐related macular degeneration via Sema4D‐PlexinB1 axis‐mediated activation of pericytes. Nat Commun 16, 2821. doi: 10.1038/s41467-025-58074-0 40121188 PMC11929803

[104] Wu JH , Li YN , Chen AQ , Hong CD , Zhang CL , Wang HL , Zhou YF , Li PC , Wang Y , Mao L *et al*. (2020) Inhibition of Sema4D/PlexinB1 signaling alleviates vascular dysfunction in diabetic retinopathy. EMBO Mol Med 12, e10154. doi: 10.15252/emmm.201810154 31943789 PMC7005627

[105] Gerke V , Gavins FNE , Geisow M , Grewal T , Jaiswal JK , Nylandsted J & Rescher U (2024) Annexins‐a family of proteins with distinctive tastes for cell signaling and membrane dynamics. Nat Commun 15, 1574. doi: 10.1038/s41467-024-45954-0 38383560 PMC10882027

[106] Fassel H , Chen H , Ruisi M , Kumar N , DeSancho M & Hajjar KA (2021) Reduced expression of annexin A2 is associated with impaired cell surface fibrinolysis and venous thromboembolism. Blood 137, 2221–2230. doi: 10.1182/blood.2020008123 33512476 PMC8063089

[107] Zhang CL , Hong CD , Wang HL , Chen AQ , Zhou YF , Wan Y , Li YN & Hu B (2020) The role of semaphorins in small vessels of the eye and brain. Pharmacol Res 160, 105044. doi: 10.1016/j.phrs.2020.105044 32590102

[108] Perälä N , Sariola H & Immonen T (2012) More than nervous: the emerging roles of plexins. Differentiation 83, 77–91. doi: 10.1016/j.diff.2011.08.001 22099179

[109] Artigiani S , Conrotto P , Fazzari P , Gilestro GF , Barberis D , Giordano S , Comoglio PM & Tamagnone L (2004) Plexin‐B3 is a functional receptor for semaphorin 5A. EMBO Rep 5, 710–714. doi: 10.1038/sj.embor.7400189 15218527 PMC1299100

[110] Segarra M , Ohnuki H , Maric D , Salvucci O , Hou X , Kumar A , Li X & Tosato G (2012) Semaphorin 6A regulates angiogenesis by modulating VEGF signaling. Blood 120, 4104–4115. doi: 10.1182/blood-2012-02-410076 23007403 PMC3496961

[111] Myllyharju J (2003) Prolyl 4‐hydroxylases, the key enzymes of collagen biosynthesis. Matrix Biol 22, 15–24. doi: 10.1016/s0945-053x(03)00006-4 12714038

[112] Vallet SD & Ricard‐Blum S (2019) Lysyl oxidases: from enzyme activity to extracellular matrix cross‐links. Essays Biochem 63, 349–364. doi: 10.1042/EBC20180050 31488698

[113] Zemskov EA , Janiak A , Hang J , Waghray A & Belkin AM (2006) The role of tissue transglutaminase in cell‐matrix interactions. Front Biosci 1, 1057–1076. doi: 10.2741/1863 16146797

[114] Telci D & Griffin M (2006) Tissue transglutaminase (TG2)‐a wound response enzyme. Front Biosci 1, 867–882. doi: 10.2741/1843 16146777

[115] Barry‐Hamilton V , Spangler R , Marshall D , McCauley S , Rodriguez HM , Oyasu M , Mikels A , Vaysberg M , Ghermazien H , Wai C *et al*. (2010) Allosteric inhibition of lysyl oxidase‐like‐2 impedes the development of a pathologic microenvironment. Nat Med 16, 1009–1017. doi: 10.1038/nm.2208 20818376

[116] Chen CW , Okada M , Proto JD , Gao X , Sekiya N , Beckman SA , Corselli M , Crisan M , Saparov A , Tobita K *et al*. (2013) Human pericytes for ischemic heart repair. Stem Cells 31, 305–316. doi: 10.1002/stem.1285 23165704 PMC3572307

[117] Tarallo S , Beltramo E , Berrone E , Dentelli P & Porta M (2010) Effects of high glucose and thiamine on the balance between matrix metalloproteinases and their tissue inhibitors in vascular cells. Acta Diabetol 47, 105–111. doi: 10.1007/s00592-009-0124-5 19404565

[118] Lafleur MA , Forsyth PA , Atkinson SJ , Murphy G & Edwards DR (2001) Perivascular cells regulate endothelial membrane type‐1 matrix metalloproteinase activity. Biochem Biophys Res Commun 282, 463–473. doi: 10.1006/bbrc.2001.4596 11401482

[119] Dave JM , Mirabella T , Weatherbee SD & Greif DM (2018) Pericyte ALK5/TIMP3 Axis contributes to endothelial morphogenesis in the developing brain. Dev Cell 44, 665–678. doi: 10.1016/j.devcel.2018.01.018. 29456135 PMC5871595

[120] Takata F , Dohgu S , Matsumoto J , Takahashi H , Machida T , Wakigawa T , Harada E , Miyaji H , Koga M , Nishioku T *et al*. (2011) Brain pericytes among cells constituting the blood‐brain barrier are highly sensitive to tumor necrosis factor‐α, releasing matrix metalloproteinase‐9 and migrating in vitro. J Neuroinflammation 26, 106. doi: 10.1186/1742-2094-8-106 PMC3182916 21867555

[121] Risman RA , Kirby NC , Bannish BE , Hudson NE & Tutwiler V (2023) Fibrinolysis: an illustrated review. Res Pract Thromb Haemost 7, 100081. doi: 10.1016/j.rpth.2023.100081 36942151 PMC10024051

[122] Lijnen HR (2001) Plasmin and matrix metalloproteinases in vascular remodeling. Thromb Haemost 86, 324–333. 11487021

[123] Stöcker W , Grams F , Baumann U , Reinemer P , Gomis‐Rüth FX , McKay DB & Bode W (1995) The metzincins‐topological and sequential relations between the astacins, adamalysins, serralysins, and matrixins (collagenases) define a superfamily of zinc‐peptidases. Protein Sci 4, 823–840. doi: 10.1002/pro.5560040502 7663339 PMC2143131

[124] Gomis‐Rüth FX (2003) Structural aspects of the metzincin clan of metalloendopeptidases. Mol Biotechnol 24, 157–202. doi: 10.1385/MB:24:2:157 12746556

[125] Rempe RG , Hartz AMS & Bauer B (2016) Matrix metalloproteinases in the brain and blood‐brain barrier: versatile breakers and makers. J Cereb Blood Flow Metab 36, 1481–1507. doi: 10.1177/0271678X16655551 27323783 PMC5012524

[126] Visse R & Nagase H (2003) Matrix metalloproteinases and tissue inhibitors of metalloproteinases: structure, function, and biochemistry. Circ Res 92, 827–839. doi: 10.1161/01.RES.0000070112.80711.3D 12730128

[127] Itoh Y (2015) Membrane‐type matrix metalloproteinases: their functions and regulations. Matrix Biol 44–46, 207–223. doi: 10.1016/j.matbio.2015.03.004 25794647

[128] Santamaria S & de Groot R (2020) ADAMTS proteases in cardiovascular physiology and disease. Open Biol 10, 200333. doi: 10.1098/rsob.200333 33352066 PMC7776578

[129] Rose KWJ , Taye N , Karoulias SZ & Hubmacher D (2021) Regulation of ADAMTS proteases. Front Mol Biosci 29, 701959. 10.3389/fmolb.2021.701959 PMC8275829 34268335

[130] Iruela‐Arispe ML , Carpizo D & Luque A (2003) ADAMTS1: a matrix metalloprotease with angioinhibitory properties. Ann N Y Acad Sci 995, 183–190. doi: 10.1111/j.1749-6632.2003.tb03221.x 12814950

[131] Luque A , Carpizo DR & Iruela‐Arispe ML (2003) ADAMTS1/METH1 inhibits endothelial cell proliferation by direct binding and sequestration of VEGF165. J Biol Chem 278, 23656–23665. doi: 10.1074/jbc.M212964200 12716911

[132] Lee NV , Sato M , Annis DS , Loo JA , Wu L , Mosher DF & Iruela‐Arispe ML (2006) ADAMTS1 mediates the release of antiangiogenic polypeptides from TSP1 and 2. EMBO J 25, 5270–5283. doi: 10.1038/sj.emboj.7601400 17082774 PMC1636613

[133] Colige A , Monseur C , Crawley JTB , Santamaria S & de Groot R (2019) Proteomic discovery of substrates of the cardiovascular protease ADAMTS7. J Biol Chem 294, 8037–8045. doi: 10.1074/jbc.RA119.007492 30926607 PMC6527163

[134] Santamaria S , Buemi F , Nuti E , Cuffaro D , De Vita E , Tuccinardi T , Rossello A , Howell S , Mehmood S , Snijders AP *et al*. (2021) Development of a fluorogenic ADAMTS‐7 substrate. J Enzyme Inhib Med Chem 36, 2160–2169. doi: 10.1080/14756366.2021.1983808 34587841 PMC8494430

[135] Cuffaro D , Burkhard T , Bernardoni BL , Di Leo R , Zhang X , Galati S , Tuccinardi T , Macchia M , Rossello A , Santamaria S *et al*. (2024) Design, synthesis and biological evaluation of arylsulfonamides as ADAMTS7 inhibitors. RSC Med Chem 15, 2806–2825. doi: 10.1039/d4md00149d 39149096 PMC11324053

[136] Edwards DR , Handsley MM & Pennington CJ (2008) The ADAM metalloproteinases. Mol Aspects Med 29, 258–289. doi: 10.1016/j.mam.2008.08.001 18762209 PMC7112278

[137] Calligaris M , Cuffaro D , Bonelli S , Spanò DP , Rossello A , Nuti E & Scilabra SD (2021) Strategies to target ADAM17 in disease: from its discovery to the iRhom revolution. Molecules 26, 944. doi: 10.3390/molecules26040944 33579029 PMC7916773

[138] Kovac A , Erickson MA & Banks WA (2011) Brain microvascular pericytes are immunoactive in culture: cytokine, chemokine, nitric oxide, and LRP‐1 expression in response to lipopolysaccharide. J Neuroinflammation 13, 139. doi: 10.1186/1742-2094-8-139 PMC3207972 21995440

[139] Eyre JJ , Williams RL & Levis HJ (2020) A human retinal microvascular endothelial‐pericyte co‐culture model to study diabetic retinopathy in vitro. Exp Eye Res 201, 108293. doi: 10.1016/j.exer.2020.108293 33039459

[140] Sahin U , Weskamp G , Kelly K , Zhou HM , Higashiyama S , Peschon J , Hartmann D , Saftig P & Blobel CP (2004) Distinct roles for ADAM10 and ADAM17 in ectodomain shedding of six EGFR ligands. J Cell Biol 164, 769–779. 14993236 10.1083/jcb.200307137 PMC2172154

[141] Stratman AN , Schwindt AE , Malotte KM & Davis GE (2010) Endothelial‐derived PDGF‐BB and HB‐EGF coordinately regulate pericyte recruitment during vasculogenic tube assembly and stabilization. Blood 116, 4720–4730. doi: 10.1182/blood-2010-05-286872 20739660 PMC2996127

[142] Isogai Z , Ono RN , Ushiro S , Keene DR , Chen Y , Mazzieri R , Charbonneau NL , Reinhardt DP , Rifkin DB & Sakai LY (2003) Latent transforming growth factor beta‐binding protein 1 interacts with fibrillin and is a microfibril‐associated protein. J Biol Chem 278, 2750–2757. doi: 10.1074/jbc.M209256200 12429738

[143] Beaufort N , Scharrer E , Kremmer E , Lux V , Ehrmann M , Huber R , Houlden H , Werring D , Haffner C & Dichgans M (2014) Cerebral small vessel disease‐related protease HtrA1 processes latent TGF‐β binding protein 1 and facilitates TGF‐β signaling. Proc Natl Acad Sci U S A 111, 16496–16501. doi: 10.1073/pnas.1418087111 25369932 PMC4246310

[144] Karamanos NK , Piperigkou Z , Passi A , Götte M , Rousselle P & Vlodavsky I (2021) Extracellular matrix‐based cancer targeting. Trends Mol Med 27, 1000–1013. doi: 10.1016/j.molmed.2021.07.009 34389240

[145] Fonović M & Turk B (2014) Cysteine cathepsins and extracellular matrix degradation. Biochim Biophys Acta 1840, 2560–2570. doi: 10.1016/j.bbagen.2014.03.017 24680817

[146] Brew K & Nagase H (2010) The tissue inhibitors of metalloproteinases (TIMPs): an ancient family with structural and functional diversity. Biochim Biophys Acta 1803, 55–71. doi: 10.1016/j.bbamcr.2010.01.003 20080133 PMC2853873

[147] Kostallari E , Baba‐Amer Y , Alonso‐Martin S , Ngoh P , Relaix F , Lafuste P & Gherardi RK (2015) Pericytes in the myovascular niche promote post‐natal myofiber growth and satellite cell quiescence. Development 142, 1242–1253. doi: 10.1242/dev.115386 25742797

[148] Saunders WB , Bohnsack BL , Faske JB , Anthis NJ , Bayless KJ , Hirschi KK & Davis GE (2006) Coregulation of vascular tube stabilization by endothelial cell TIMP‐2 and pericyte TIMP‐3. J Cell Biol 175, 179–191. doi: 10.1083/jcb.200603176 17030988 PMC2064509

[149] Minns AF , Qi Y , Yamamoto K , Lee K , Ahnström J & Santamaria S (2023) The C‐terminal domains of ADAMTS1 contain exosites involved in its proteoglycanase activity. J Biol Chem 299, 103048. doi: 10.1016/j.jbc.2023.103048 36813235 PMC10033314

[150] Hashimoto G , Aoki T , Nakamura H , Tanzawa K & Okada Y (2001) Inhibition of ADAMTS4 (aggrecanase‐1) by tissue inhibitors of metalloproteinases (TIMP‐1, 2, 3 and 4). FEBS Lett 494, 192–195. doi: 10.1016/s0014-5793(01)02323-7 11311239

[151] Kashiwagi M , Tortorella M , Nagase H & Brew K (2001) TIMP‐3 is a potent inhibitor of aggrecanase 1 (ADAM‐TS4) and aggrecanase 2 (ADAM‐TS5). J Biol Chem 276, 12501–12504. doi: 10.1074/jbc.C000848200 11278243

[152] Martin DR , Sardelli G , Burkhard T , Fowkes MM , Minns AF , Moschini R , Del Corso A , de Groot R , Apte SS & Santamaria S (2025) Characterization of ADAMTS9 proteoglycanase activity: comparison with ADAMTS1, ADAMTS4, and ADAMTS5. J Biol Chem 301, 110301. doi: 10.1016/j.jbc.2025.110301 40449594 PMC12226136

[153] Amour A , Slocombe PM , Webster A , Butler M , Knight CG , Smith BJ , Stephens PE , Shelley C , Hutton M , Knäuper V *et al*. (1998) TNF‐alpha converting enzyme (TACE) is inhibited by TIMP‐3. FEBS Lett 435, 39–44. doi: 10.1016/s0014-5793(98)01031-x 9755855

[154] Yu WH , Yu S , Meng Q , Brew K & Woessner JF Jr (2000) TIMP‐3 binds to sulfated glycosaminoglycans of the extracellular matrix. J Biol Chem 275, 31226–31232. doi: 10.1074/jbc.M000907200 10900194

[155] Qi JH , Ebrahem Q , Moore N , Murphy G , Claesson‐Welsh L , Bond M , Baker A & Anand‐Apte B (2003) A novel function for tissue inhibitor of metalloproteinases‐3 (TIMP3): inhibition of angiogenesis by blockage of VEGF binding to VEGF receptor‐2. Nat Med 9, 407–415. doi: 10.1038/nm846 12652295

[156] Qi JH , Ebrahem Q , Ali M , Cutler A , Bell B , Prayson N , Sears J , Knauper V , Murphy G & Anand‐Apte B (2013) Tissue inhibitor of metalloproteinases‐3 peptides inhibit angiogenesis and choroidal neovascularization in mice. PLoS One 8, e55667. doi: 10.1371/journal.pone.0055667 23469166 PMC3585964

[157] van Gent D , Sharp P , Morgan K & Kalsheker N (2003) Serpins: structure, function and molecular evolution. Int J Biochem Cell Biol 35, 1536–1547. doi: 10.1016/s1357-2725(03)00134-1 12824063

[158] Kemp SS , Aguera KN , Cha B & Davis GE (2020) Defining endothelial cell‐derived factors that promote Pericyte recruitment and capillary network assembly. Arterioscler Thromb Vasc Biol 40, 2632–2648. doi: 10.1161/ATVBAHA.120.314948 32814441 PMC7939110

[159] Shimizu F , Sano Y , Abe MA , Maeda T , Ohtsuki S , Terasaki T & Kanda T (2011) Peripheral nerve pericytes modify the blood‐nerve barrier function and tight junctional molecules through the secretion of various soluble factors. J Cell Physiol 226, 255–266. doi: 10.1002/jcp.22337 20665675

[160] Tu Z , Li Y , Smith DS , Sheibani N , Huang S , Kern T & Lin F (2011) Retinal pericytes inhibit activated T cell proliferation. Invest Ophthalmol Vis Sci 52, 9005–9010. doi: 10.1167/iovs.11-8008 22003106 PMC3231798

[161] Zheng K , Lin L , Jiang W , Chen L , Zhang X , Zhang Q , Ren Y & Hao J (2022) Single‐cell RNA‐seq reveals the transcriptional landscape in ischemic stroke. J Cereb Blood Flow Metab 42, 56–73. doi: 10.1177/0271678X211026770 34496660 PMC8721774

[162] Dobie R , Wilson‐Kanamori JR , Henderson BEP , Smith JR , Matchett KP , Portman JR , Wallenborg K , Picelli S , Zagorska A , Pendem SV *et al*. (2019) Single‐cell Transcriptomics uncovers zonation of function in the mesenchyme during liver fibrosis. Cell Rep 29, 1832–1847. doi: 10.1016/j.celrep.2019.10.024 31722201 PMC6856722

[163] Teichert M , Milde L , Holm A , Stanicek L , Gengenbacher N , Savant S , Ruckdeschel T , Hasanov Z , Srivastava K , Hu J *et al*. (2017) Pericyte‐expressed Tie2 controls angiogenesis and vessel maturation. Nat Commun 18, 16106. doi: 10.1038/ncomms16106 PMC5520106 28719590

[164] Hellström M , Gerhardt H , Kalén M , Li X , Eriksson U , Wolburg H & Betsholtz C (2001) Lack of pericytes leads to endothelial hyperplasia and abnormal vascular morphogenesis. J Cell Biol 153, 543–553. doi: 10.1083/jcb.153.3.543 11331305 PMC2190573

[165] Bjarnegård M , Enge M , Norlin J , Gustafsdottir S , Fredriksson S , Abramsson A , Takemoto M , Gustafsson E , Fässler R & Betsholtz C (2004) Endothelium‐specific ablation of PDGFB leads to pericyte loss and glomerular, cardiac and placental abnormalities. Development 131, 1847–1857. doi: 10.1242/dev.01080 15084468

[166] Augustin HG , Koh GY , Thurston G & Alitalo K (2009) Control of vascular morphogenesis and homeostasis through the angiopoietin‐tie system. Nat Rev Mol Cell Biol 10, 165–177. doi: 10.1038/nrm2639 19234476

[167] Stenzel D , Nye E , Nisancioglu M , Adams RH , Yamaguchi Y & Gerhardt H (2009) Peripheral mural cell recruitment requires cell‐autonomous heparan sulfate. Blood 114, 915–924. doi: 10.1182/blood-2008-10-186239 19398718

[168] Eklund L & Olsen BR (2006) Tie receptors and their angiopoietin ligands are context‐dependent regulators of vascular remodeling. Exp Cell Res 312, 630–641. doi: 10.1016/j.yexcr.2005.09.002 16225862

[169] Sato TN , Tozawa Y , Deutsch U , Wolburg‐Buchholz K , Fujiwara Y , Gendron‐Maguire M , Gridley T , Wolburg H , Risau W & Qin Y (1995) Distinct roles of the receptor tyrosine kinases Tie‐1 and Tie‐2 in blood vessel formation. Nature 376, 70–74. doi: 10.1038/376070a0 7596437

[170] Navarro R , Compte M , Álvarez‐Vallina L & Sanz L (2016) Immune regulation by Pericytes: modulating innate and adaptive immunity. Front Immunol 4, 480. doi: 10.3389/fimmu.2016.00480 PMC5095456 27867386

[171] Lindahl P , Johansson BR , Levéen P & Betsholtz C (1997) Pericyte loss and microaneurysm formation in PDGF‐B‐deficient mice. Science 277, 242–245. doi: 10.1126/science.277.5323.242 9211853

[172] Ausprunk DH & Folkman J (1977) Migration and proliferation of endothelial cells in preformed and newly formed blood vessels during tumor angiogenesis. Microvasc Res 14, 53–65. doi: 10.1016/0026-2862(77)90141-8 895546

[173] Errede M , Mangieri D , Longo G , Girolamo F , De Trizio I , Vimercati A , Serio G , Frei K , Perris R & Virgintino D (2018) Tunneling nanotubes evoke pericyte/endothelial communication during normal and tumoral angiogenesis. Fluids Barriers CNS 15, 28. doi: 10.1186/s12987-018-0114-5 30290761 PMC6173884

[174] von Tell D , Armulik A & Betsholtz C (2006) Pericytes and vascular stability. Exp Cell Res 312, 623–629. doi: 10.1016/j.yexcr.2005.10.019 16303125

[175] Levy DE , Lapierre F , Liang W , Ye W , Lange CW , Li X , Grobelny D , Casabonne M , Tyrrell D , Holme K *et al*. (1998) Matrix metalloproteinase inhibitors: a structure‐activity study. J Med Chem 41, 199–223. doi: 10.1021/jm970494j 9457244

[176] Piperigkou Z , Mangani S , Kremmydas S , Koletsis NE & Karamanos NK (2025) A guide to the types, structures, and multifaceted functions of matrix metalloproteinases in cancer. FEBS J 293, 3717–3757. doi: 10.1111/febs.70296 41159838 PMC13326533

[177] You WK , Yotsumoto F , Sakimura K , Adams RH & Stallcup WB (2014) NG2 proteoglycan promotes tumor vascularization via integrin‐dependent effects on pericyte function. Angiogenesis 17, 61–76. doi: 10.1007/s10456-013-9378-1 23925489 PMC3898355

[178] Feng D , Zhou J , Liu H , Wu X , Li F , Zhao J , Zhang Y , Wang L , Chao M , Wang Q *et al*. (2022) Astrocytic NDRG2‐PPM1A interaction exacerbates blood‐brain barrier disruption after subarachnoid hemorrhage. Sci Adv 8, eabq2423. doi: 10.1126/sciadv.abq2423 36179025 PMC9524825

[179] Chantrain CF , Henriet P , Jodele S , Emonard H , Feron O , Courtoy PJ , DeClerck YA & Marbaix E (2006) Mechanisms of pericyte recruitment in tumour angiogenesis: a new role for metalloproteinases. Eur J Cancer 42, 310–318. doi: 10.1016/j.ejca.2005.11.010 16406506

[180] Courtoy PJ & Boyles J (1983) Fibronectin in the microvasculature: localization in the pericyte‐endothelial interstitium. J Ultrastruct Res 83, 258–273. doi: 10.1016/s0022-5320(83)90133-8 6348302

[181] Bergers G , Brekken R , McMahon G , Vu TH , Itoh T , Tamaki K , Tanzawa K , Thorpe P , Itohara S , Werb Z *et al*. (2000) Matrix metalloproteinase‐9 triggers the angiogenic switch during carcinogenesis. Nat Cell Biol 2, 737–744. doi: 10.1038/35036374 11025665 PMC2852586

[182] Voisin MB , Pröbstl D & Nourshargh S (2010) Venular basement membranes ubiquitously express matrix protein low‐expression regions: characterization in multiple tissues and remodeling during inflammation. Am J Pathol 176, 482–495. doi: 10.2353/ajpath.2010.090510 20008148 PMC2797906

[183] Ayres‐Sander CE , Lauridsen H , Maier CL , Sava P , Pober JS & Gonzalez AL (2013) Transendothelial migration enables subsequent transmigration of neutrophils through underlying pericytes. PLoS One 8, e60025. doi: 10.1371/journal.pone.0060025 23555870 PMC3608600

[184] Lauridsen HM , Pober JS & Gonzalez AL (2014) A composite model of the human postcapillary venule for investigation of microvascular leukocyte recruitment. FASEB J 28, 1166–1180. doi: 10.1096/fj.13-240986 24297702 PMC3929680

[185] Schlaepfer DD , Broome MA & Hunter T (1997) Fibronectin‐stimulated signaling from a focal adhesion kinase‐c‐Src complex: involvement of the Grb2, p130cas, and Nck adaptor proteins. Mol Cell Biol 17, 1702–1713. doi: 10.1128/MCB.17.3.1702 9032297 PMC231895

[186] Liu L , Ratner BD , Sage EH & Jiang S (2007) Endothelial cell migration on surface‐density gradients of fibronectin, VEGF, or both proteins. Langmuir 23, 11168–11173. doi: 10.1021/la701435x 17892312

[187] Reijerkerk A , Kooij G , van der Pol SM , Khazen S , Dijkstra CD & de Vries HE (2006) Diapedesis of monocytes is associated with MMP‐mediated occludin disappearance in brain endothelial cells. FASEB J 20, 2550–2552. doi: 10.1096/fj.06-6099fje 17065217

[188] Ozerdem U & Stallcup WB (2004) Pathological angiogenesis is reduced by targeting pericytes via the NG2 proteoglycan. Angiogenesis 7, 269–276. doi: 10.1007/s10456-004-4182-6 15609081 PMC1350818

[189] Huang FJ , You WK , Bonaldo P , Seyfried TN , Pasquale EB & Stallcup WB (2010) Pericyte deficiencies lead to aberrant tumor vascularizaton in the brain of the NG2 null mouse. Dev Biol 344, 1035–1046. doi: 10.1016/j.ydbio.2010.06.023 20599895 PMC3197744

[190] Gibby K , You WK , Kadoya K , Helgadottir H , Young LJ , Ellies LG , Chang Y , Cardiff RD & Stallcup WB (2012) Early vascular deficits are correlated with delayed mammary tumorigenesis in the MMTV‐PyMT transgenic mouse following genetic ablation of the NG2 proteoglycan. Breast Cancer Res 14, R67. doi: 10.1186/bcr3174 22531600 PMC3446402

[191] Sweeney MD , Ayyadurai S & Zlokovic BV (2016) Pericytes of the neurovascular unit: key functions and signaling pathways. Nat Neurosci 19, 771–783. doi: 10.1038/nn.4288 27227366 PMC5745011

[192] Uemura MT , Maki T , Ihara M , Lee VMY & Trojanowski JQ (2020) Brain microvascular Pericytes in vascular cognitive impairment and dementia. Front Aging Neurosci 14, 80. doi: 10.3389/fnagi.2020.00080 PMC7171590 32317958

[193] Peppiatt CM , Howarth C , Mobbs P & Attwell D (2006) Bidirectional control of CNS capillary diameter by pericytes. Nature 443, 700–704. doi: 10.1038/nature05193 17036005 PMC1761848

[194] Levéen P , Pekny M , Gebre‐Medhin S , Swolin B , Larsson E & Betsholtz C (1994) Mice deficient for PDGF B show renal, cardiovascular, and hematological abnormalities. Genes Dev 8, 1875–1887. doi: 10.1101/gad.8.16.1875 7958863

[195] Soriano P (1994) Abnormal kidney development and hematological disorders in PDGF beta‐receptor mutant mice. Genes Dev 8, 1888–1896. doi: 10.1101/gad.8.16.1888 7958864

[196] Tallquist MD , French WJ & Soriano P (2003) Additive effects of PDGF receptor beta signaling pathways in vascular smooth muscle cell development. PLoS Biol 1, E52. doi: 10.1371/journal.pbio.0000052 14624252 PMC261889

[197] Tiraboschi P , Hansen LA , Thal LJ & Corey‐Bloom J (2004) The importance of neuritic plaques and tangles to the development and evolution of AD. Neurology 62, 1984–1989. doi: 10.1212/01.wnl.0000129697.01779.0a 15184601

[198] Thomsen MS , Routhe LJ & Moos T (2017) The vascular basement membrane in the healthy and pathological brain. J Cereb Blood Flow Metab 37, 3300–3317. doi: 10.1177/0271678X17722436 28753105 PMC5624399

[199] Verbeek MM , de Waal RM , Schipper JJ & Van Nostrand WE (1997) Rapid degeneration of cultured human brain pericytes by amyloid beta protein. J Neurochem 68, 1135–1141. doi: 10.1046/j.1471-4159.1997.68031135.x 9048759

[200] Castillo GM , Ngo C , Cummings J , Wight TN & Snow AD (1997) Perlecan binds to the beta‐amyloid proteins (a beta) of Alzheimer's disease, accelerates a beta fibril formation, and maintains a beta fibril stability. J Neurochem 69, 2452–2465. doi: 10.1046/j.1471-4159.1997.69062452.x 9375678

[201] Schultz N , Nielsen HM , Minthon L & Wennström M (2014) Involvement of matrix metalloproteinase‐9 in amyloid‐β 1‐42‐induced shedding of the pericyte proteoglycan NG2. J Neuropathol Exp Neurol 73, 684–692. doi: 10.1097/NEN.0000000000000084 24918635 PMC4072439

[202] Sagare AP , Sweeney MD , Makshanoff J & Zlokovic BV (2015) Shedding of soluble platelet‐derived growth factor receptor‐β from human brain pericytes. Neurosci Lett 21, 97–101. doi: 10.1016/j.neulet.2015.09.025 PMC4631673 26407747

[203] Zlokovic BV , Deane R , Sagare AP , Bell RD & Winkler EA (2010) Low‐density lipoprotein receptor‐related protein‐1: a serial clearance homeostatic mechanism controlling Alzheimer's amyloid β‐peptide elimination from the brain. J Neurochem 115, 1077–1089. doi: 10.1111/j.1471-4159.2010.07002.x 20854368 PMC2972355

[204] Yan P , Hu X , Song H , Yin K , Bateman RJ , Cirrito JR , Xiao Q , Hsu FF , Turk JW , Xu J *et al*. (2006) Matrix metalloproteinase‐9 degrades amyloid‐beta fibrils in vitro and compact plaques in situ. J Biol Chem 281, 24566–24574. doi: 10.1074/jbc.M602440200 16787929

[205] White AR , Du T , Laughton KM , Volitakis I , Sharples RA , Xilinas ME , Hoke DE , Holsinger RM , Evin G , Cherny RA *et al*. (2006) Degradation of the Alzheimer disease amyloid beta‐peptide by metal‐dependent up‐regulation of metalloprotease activity. J Biol Chem 281, 17670–17680. doi: 10.1074/jbc.M602487200 16648635

[206] Göritz C , Dias DO , Tomilin N , Barbacid M , Shupliakov O & Frisén J (2011) A pericyte origin of spinal cord scar tissue. Science 333, 238–242. doi: 10.1126/science.1203165 21737741

[207] Dias DO , Kalkitsas J , Kelahmetoglu Y , Estrada CP , Tatarishvili J , Holl D , Jansson L , Banitalebi S , Amiry‐Moghaddam M , Ernst A *et al*. (2021) Pericyte‐derived fibrotic scarring is conserved across diverse central nervous system lesions. Nat Commun 12, 5501. doi: 10.1038/s41467-021-25585-5 34535655 PMC8448846

[208] Dias DO , Kim H , Holl D , Werne Solnestam B , Lundeberg J , Carlén M , Göritz C & Frisén J (2018) Reducing Pericyte‐derived scarring promotes recovery after spinal cord injury. Cell 173, 153–165. doi: 10.1016/j.cell.2018.02.004 29502968 PMC5871719

[209] Rustenhoven J , Aalderink M , Scotter EL , Oldfield RL , Bergin PS , Mee EW , Graham ES , Faull RL , Curtis MA , Park TI *et al*. (2016) TGF‐beta1 regulates human brain pericyte inflammatory processes involved in neurovasculature function. J Neuroinflammation 11, 37. doi: 10.1186/s12974-016-0503-0 PMC4751726 26867675

[210] Takahashi Y , Maki T , Liang AC , Itoh K , Lok J , Osumi N & Arai K (2014) p38 MAP kinase mediates transforming‐growth factor‐β1‐induced upregulation of matrix metalloproteinase‐9 but not ‐2 in human brain pericytes. Brain Res 17, 1–8. doi: 10.1016/j.brainres.2014.10.029 PMC4254496 25451097

[211] De La Fuente AG , Lange S , Silva ME , Gonzalez GA , Tempfer H , van Wijngaarden P , Zhao C , Di Canio L , Trost A , Bieler L *et al*. (2017) Pericytes stimulate oligodendrocyte progenitor cell differentiation during CNS Remyelination. Cell Rep 20, 1755–1764. doi: 10.1016/j.celrep.2017.08.007 28834740 PMC5574064

[212] Silva ME , Lange S , Hinrichsen B , Philp AR , Reyes CR , Halabi D , Mansilla JB , Rotheneichner P , Guzman de la Fuente A , Couillard‐Despres S *et al*. (2019) Pericytes favor oligodendrocyte fate choice in adult neural stem cells. Front Cell Neurosci 27, 85. doi: 10.3389/fncel.2019.00085 PMC6446960 30971893

[213] Campbell BCV & Khatri P (2020) Stroke Lancet 396, 129–142. doi: 10.1016/S0140-6736(20)31179-X 32653056

[214] Fernández‐Klett F , Potas JR , Hilpert D , Blazej K , Radke J , Huck J , Engel O , Stenzel W , Genové G & Priller J (2013) Early loss of pericytes and perivascular stromal cell‐induced scar formation after stroke. J Cereb Blood Flow Metab 33, 428–439. doi: 10.1038/jcbfm.2012.187 23250106 PMC3587816

[215] Milner R & Campbell IL (2006) Increased expression of the beta4 and alpha5 integrin subunits in cerebral blood vessels of transgenic mice chronically producing the pro‐inflammatory cytokines IL‐6 or IFN‐alpha in the central nervous system. Mol Cell Neurosci 33, 429–440. doi: 10.1016/j.mcn.2006.09.004 17049262 PMC1847624

[216] Cailhier JF , Sirois I , Laplante P , Lepage S , Raymond MA , Brassard N , Prat A , Iozzo RV , Pshezhetsky AV & Hébert MJ (2008) Caspase‐3 activation triggers extracellular cathepsin L release and endorepellin proteolysis. J Biol Chem 283, 27220–27229. doi: 10.1074/jbc.M801164200 18658137

[217] Thanabalasundaram G , Schneidewind J , Pieper C & Galla HJ (2011) The impact of pericytes on the blood‐brain barrier integrity depends critically on the pericyte differentiation stage. Int J Biochem Cell Biol 43, 1284–1293. doi: 10.1016/j.biocel.2011.05.002 21601005

[218] Barr TL , Latour LL , Lee KY , Schaewe TJ , Luby M , Chang GS , El‐Zammar Z , Alam S , Hallenbeck JM , Kidwell CS *et al*. (2010) Blood‐brain barrier disruption in humans is independently associated with increased matrix metalloproteinase‐9. Stroke 41, e123–e128. doi: 10.1161/STROKEAHA.109.570515 20035078 PMC2827673

[219] Weekman EM & Wilcock DM (2016) Matrix metalloproteinase in blood‐brain barrier breakdown in dementia. J Alzheimer's Dis 49, 893–903. doi: 10.3233/JAD-150759 26599057

[220] Underly RG , Levy M , Hartmann DA , Grant RI , Watson AN & Shih AY (2017) Pericytes as inducers of rapid, matrix Metalloproteinase‐9‐dependent capillary damage during ischemia. J Neurosci 37, 129–140. doi: 10.1523/JNEUROSCI.2891-16.2016 28053036 PMC5214626

[221] Yang Y , Estrada EY , Thompson JF , Liu W & Rosenberg GA (2007) Matrix metalloproteinase‐mediated disruption of tight junction proteins in cerebral vessels is reversed by synthetic matrix metalloproteinase inhibitor in focal ischemia in rat. J Cereb Blood Flow Metab 27, 697–709. doi: 10.1038/sj.jcbfm.9600375 16850029

[222] Mun‐Bryce S & Rosenberg GA (1998) Matrix Metalloproteinases in cerebrovascular disease. J Cereb Blood Flow Metab 18, 1163–1172. doi: 10.1097/00004647-199811000-00001 9809504

[223] Fukuda S , Fini CA , Mabuchi T , Koziol JA , Eggleston LL Jr & del Zoppo GJ (2004) Focal cerebral ischemia induces active proteases that degrade microvascular matrix. Stroke 35, 998–1004. doi: 10.1161/01.STR.0000119383.76447.05 15001799 PMC2979008

[224] Machida T , Dohgu S , Takata F , Matsumoto J , Kimura I , Koga M , Nakamoto K , Yamauchi A & Kataoka Y (2017) Role of thrombin‐PAR1‐PKCθ/δ axis in brain pericytes in thrombin‐induced MMP‐9 production and blood‐brain barrier dysfunction in vitro. Neuroscience 14, 146–157. doi: 10.1016/j.neuroscience.2017.03.026 28344073

[225] Schulz GB , Wieland E , Wüstehube‐Lausch J , Boulday G , Moll I , Tournier‐Lasserve E & Fischer A (2015) Cerebral cavernous Malformation‐1 protein controls DLL4‐Notch3 Signaling between the endothelium and Pericytes. Stroke 46, 1337–1343. doi: 10.1161/STROKEAHA.114.007512 25791711

[226] Nees S , Weiss DR , Senftl A , Knott M , Förch S , Schnurr M , Weyrich P & Juchem G (2012) Isolation, bulk cultivation, and characterization of coronary microvascular pericytes: the second most frequent myocardial cell type in vitro. Am J Physiol Heart Circ Physiol 302, H69–H84. doi: 10.1152/ajpheart.00359.2011 22037185

[227] Avolio E , Campagnolo P , Katare R & Madeddu P (2024) The role of cardiac pericytes in health and disease: therapeutic targets for myocardial infarction. Nat Rev Cardiol 21, 106–118. doi: 10.1038/s41569-023-00913-y 37542118

[228] Lee LL , Khakoo AY & Chintalgattu V (2021) Cardiac pericytes function as key vasoactive cells to regulate homeostasis and disease. FEBS Open Bio 11, 207–225. doi: 10.1002/2211-5463.13021 PMC7780101 33135334

[229] Frangogiannis NG (2024) The fate and role of the pericytes in myocardial diseases. Eur J Clin Invest 54, e14204. doi: 10.1111/eci.14204 38586936

[230] Pettersson A , Nagy JA , Brown LF , Sundberg C , Morgan E , Jungles S , Carter R , Krieger JE , Manseau EJ , Harvey VS *et al*. (2000) Heterogeneity of the angiogenic response induced in different normal adult tissues by vascular permeability factor/vascular endothelial growth factor. Lab Invest 80, 99–115. doi: 10.1038/labinvest.3780013 10653008

[231] Siao CJ , Lorentz CU , Kermani P , Marinic T , Carter J , McGrath K , Padow VA , Mark W , Falcone DJ , Cohen‐Gould L *et al*. (2012) ProNGF, a cytokine induced after myocardial infarction in humans, targets pericytes to promote microvascular damage and activation. J Exp Med 209, 2291–2305. doi: 10.1084/jem.20111749 23091165 PMC3501352

[232] Lee SJ , Lee CK , Kang S , Park I , Kim YH , Kim SK , Hong SP , Bae H , He Y , Kubota Y *et al*. (2018) Angiopoietin‐2 exacerbates cardiac hypoxia and inflammation after myocardial infarction. J Clin Invest 128, 5018–5033. doi: 10.1172/JCI99659 30295643 PMC6205384

[233] Zymek P , Bujak M , Chatila K , Cieslak A , Thakker G , Entman ML & Frangogiannis NG (2006) The role of platelet‐derived growth factor signaling in healing myocardial infarcts. J Am Coll Cardiol 48, 2315–2323. doi: 10.1016/j.jacc.2006.07.060 17161265

[234] Birbrair A , Zhang T , Files DC , Mannava S , Smith T , Wang ZM , Messi ML , Mintz A & Delbono O (2014) Type‐1 pericytes accumulate after tissue injury and produce collagen in an organ‐dependent manner. Stem Cell Res Ther 5, 122. doi: 10.1186/scrt512 25376879 PMC4445991

[235] Katare R , Riu F , Mitchell K , Gubernator M , Campagnolo P , Cui Y , Fortunato O , Avolio E , Cesselli D , Beltrami AP *et al*. (2011) Transplantation of human pericyte progenitor cells improves the repair of infarcted heart through activation of an angiogenic program involving micro‐RNA‐132. Circ Res 109, 894–906. doi: 10.1161/CIRCRESAHA.111.251546 21868695 PMC3623091

[236] Cogan DG , Toussaint D & Kuwabara T (1961) Retinal vascular patterns. IV Diabetic Retinopathy Arch Ophthalmol 66, 366–378. doi: 10.1001/archopht.1961.00960010368014 13694291

[237] D'Esposito F , Cappellani F , Visalli F , Capobianco M , Rapisarda L , Avitabile A , Cannizzaro L , Malaguarnera R , Gagliano G , Maniaci A *et al*. (2025) Pericytes as key players in retinal diseases: a comprehensive narrative review. Biol‐Basel 14, 736. doi: 10.3390/biology14070736 PMC12292761 40723297

[238] Roy S , Sato T , Paryani G & Kao R (2003) Downregulation of fibronectin overexpression reduces basement membrane thickening and vascular lesions in retinas of galactose‐fed rats. Diabetes 52, 1229–1234. doi: 10.2337/diabetes.52.5.1229 12716757

[239] Resnikoff HA , Miller CG & Schwarzbauer JE (2022) Implications of fibrotic extracellular matrix in diabetic retinopathy. Exp Biol Med 247, 1093–1102. doi: 10.1177/15353702221087175 PMC9335512 35410521

[240] Gariano RF & Gardner TW (2005) Retinal angiogenesis in development and disease. Nature 438, 960–966. doi: 10.1038/nature04482 16355161

[241] Ashton N (1951) Retinal micro‐aneurysms in the non‐diabetic subject. Br J Ophthalmol 35, 189–212. doi: 10.1136/bjo.35.4.189 14830727 PMC1323722

[242] Ashton N (1963) Studies of the retinal capillaries in relation to diabetic and other retinopathies. Br J Ophthalmol 47, 521–538. doi: 10.1136/bjo.47.9.521 14189723 PMC505844

[243] López‐Luppo M , Nacher V , Ramos D , Catita J , Navarro M , Carretero A , Rodriguez‐Baeza A , Mendes‐Jorge L & Ruberte J (2017) Blood vessel basement membrane alterations in human retinal microaneurysms during aging. Invest Ophthalmol Vis Sci 58, 1116–1131. doi: 10.1167/iovs.16-19998 28196225

[244] Mandarino LJ , Sundarraj N , Finlayson J & Hassell HR (1993) Regulation of fibronectin and laminin synthesis by retinal capillary endothelial cells and pericytes in vitro. Exp Eye Res 57, 609–621. doi: 10.1006/exer.1993.1166 8282048

[245] Hammes HP (2005) Pericytes and the pathogenesis of diabetic retinopathy. Horm Metab Res 37 (Suppl 1), 39–43. doi: 10.1055/s-2005-861361 15918109

[246] Rübsam A , Parikh S & Fort PE (2018) Role of inflammation in diabetic retinopathy. Int J Mol Sci 19, 942. doi: 10.3390/ijms19040942 29565290 PMC5979417

[247] Lin SL , Chang FC , Schrimpf C , Chen YT , Wu CF , Wu VC , Chiang WC , Kuhnert F , Kuo CJ , Chen YM *et al*. (2011) Targeting endothelium‐pericyte cross talk by inhibiting VEGF receptor signaling attenuates kidney microvascular rarefaction and fibrosis. Am J Pathol 178, 911–923. doi: 10.1016/j.ajpath.2010.10.012 21281822 PMC3070546

[248] Basile DP (2004) Rarefaction of peritubular capillaries following ischemic acute renal failure: a potential factor predisposing to progressive nephropathy. Curr Opin Nephrol Hypertens 13, 1–7. doi: 10.1097/00041552-200401000-00001 15090853

[249] Chen YT , Chang FC , Wu CF , Chou YH , Hsu HL , Chiang WC , Shen J , Chen YM , Wu KD , Tsai TJ *et al*. (2011) Platelet‐derived growth factor receptor signaling activates pericyte‐myofibroblast transition in obstructive and post‐ischemic kidney fibrosis. Kidney Int 80, 1170–1181. doi: 10.1038/ki.2011.208 21716259

[250] Jung YJ , Kim DH , Lee AS , Lee S , Kang KP , Lee SY , Jang KY , Sung MJ , Park SK & Kim W (2009) Peritubular capillary preservation with COMP‐angiopoietin‐1 decreases ischemia‐reperfusion‐induced acute kidney injury. Am J Physiol Renal Physiol 297, F952–F960. doi: 10.1152/ajprenal.00064.2009 19656917

[251] Wühl E & Schaefer F (2011) Managing kidney disease with blood‐pressure control. Nat Rev Nephrol 7, 434–444. doi: 10.1038/nrneph.2011.73 21691318

[252] Caulfield JP & Farquhar MG (1974) The permeability of glomerular capillaries to graded dextrans. Identification of the basement membrane as the primary filtration barrier. J Cell Biol 63, 883–903. doi: 10.1083/jcb.63.3.883 4612049 PMC2109376

[253] Kuppe C , Ibrahim MM , Kranz J , Zhang X , Ziegler S , Perales‐Patón J , Jansen J , Reimer KC , Smith JR , Dobie R *et al*. (2021) Decoding myofibroblast origins in human kidney fibrosis. Nature 589, 281–286. doi: 10.1038/s41586-020-2941-1 33176333 PMC7611626

[254] Sasson A , Rachi E , Sakhneny L , Baer D , Lisnyansky M , Epshtein A & Landsman L (2016) Islet Pericytes are required for β‐cell maturity. Diabetes 65, 3008–3014. doi: 10.2337/db16-0365 27388217

[255] Almaça J , Weitz J , Rodriguez‐Diaz R , Pereira E & Caicedo A (2018) The Pericyte of the pancreatic islet regulates capillary diameter and local blood flow. Cell Metab 27, 630–644. doi: 10.1016/j.cmet.2018.02.016 29514070 PMC5876933

[256] Schonblum A , Ali Naser D , Ovadia S , Egbaria M , Puyesky S , Epshtein A , Wald T , Mercado‐Medrez S , Ashery‐Padan R & Landsman L (2024) Beneficial islet inflammation in health depends on pericytic TLR/MyD88 signaling. J Clin Invest 134, e179335. doi: 10.1172/JCI179335 38885342 PMC11245159

[257] Burganova G , Schonblum A , Sakhneny L , Epshtein A , Wald T , Tzaig M & Landsman L (2023) Pericytes modulate islet immune cells and insulin secretion through Interleukin‐33 production in mice. Front Endocrinol 9, 1142988. doi: 10.3389/fendo.2023.1142988 PMC10034381 36967785

[258] Sakhneny L , Rachi E , Epshtein A , Guez HC , Wald‐Altman S , Lisnyansky M , Khalifa‐Malka L , Hazan A , Baer D , Priel A *et al*. (2018) Pancreatic Pericytes support β‐cell function in a Tcf7l2‐dependent manner. Diabetes 67, 437–447. doi: 10.2337/db17-0697 29246974

[259] Guney MA , Petersen CP , Boustani A , Duncan MR , Gunasekaran U , Menon R , Warfield C , Grotendorst GR , Means AL , Economides AN *et al*. (2011) Connective tissue growth factor acts within both endothelial cells and beta cells to promote proliferation of developing beta cells. Proc Natl Acad Sci USA 108, 15242–15247. doi: 10.1073/pnas.1100072108 21876171 PMC3174622

[260] Bi H , Ma R , Chen Z , Wang Y & Ding X (2025) The stem cell within the vessel wall: multipotent pericytes modulating β‐cell function and diabetic complications. Stem Cell Res Ther 16, 568. doi: 10.1186/s13287-025-04669-9 41094620 PMC12522955

[261] Mateus Gonçalves L , Fahd Qadir MM , Boulina M , Makhmutova M , Pereira E & Almaça J (2023) Pericyte dysfunction and impaired vasomotion are hallmarks of islets during the pathogenesis of type 1 diabetes. Cell Rep 42, 112913. doi: 10.1016/j.celrep.2023.112913 37531253 PMC10529889

[262] Friedman SL & Pinzani M (2022) Hepatic fibrosis 2022: unmet needs and a blueprint for the future. Hepatology 75, 473–488. doi: 10.1002/hep.32285 34923653 PMC12179971

[263] Perepelyuk M , Terajima M , Wang AY , Georges PC , Janmey PA , Yamauchi M & Wells RG (2013) Hepatic stellate cells and portal fibroblasts are the major cellular sources of collagens and lysyl oxidases in normal liver and early after injury. Am J Physiol Gastrointest Liver Physiol 304, G605–G614. doi: 10.1152/ajpgi.00222.2012 23328207 PMC3602686

[264] Zhang W , Wu W , Zhang N , Li H , Sun Y , Ge X , Han H , Chen S , Xu A , Komakula SSB *et al*. (2025) Hepatic stellate cell‐derived microfibrillar‐associated protein 2 prevents liver fibrosis by regulating extracellular matrix and inflammation. Theranostics 15, 4033–4053. doi: 10.7150/thno.109771 40213670 PMC11980660

[265] Yasui Y , Sato‐Matsubara M , Enomoto M , Matsubara T , Kosugi M , Inoue K , Hoang TH , Yuasa H , Fujii H , Daikoku A *et al*. (2025) Plasma Fibulin‐5 as a novel marker for advanced fibrosis in chronic hepatitis C. Gastro Hep Adv 5, 100827. doi: 10.1016/j.gastha.2025.100827 41362817 PMC12681702

[266] Tsuchida T & Friedman SL (2017) Mechanisms of hepatic stellate cell activation. Nat Rev Gastroenterol Hepatol 14, 397–411. doi: 10.1038/nrgastro.2017.38 28487545

[267] An P , Wei G , Huang P , Matta H , Li W , Lin Y , Wang J , Gretchen B & Popov YV (2025) A novel cell‐permeable LOXL2 inhibitor PAT‐1251 potently suppresses biliary liver fibrosis via collagen crosslinking‐dependent and ‐independent mechanisms. Hepatol Commun 10, e0863. doi: 10.1097/HC9.0000000000000863 41385725 PMC12705046

[268] Fowell AJ , Collins JE , Duncombe DR , Pickering JA , Rosenberg WM & Benyon RC (2011) Silencing tissue inhibitors of metalloproteinases (TIMPs) with short interfering RNA reveals a role for TIMP‐1 in hepatic stellate cell proliferation. Biochem Biophys Res Commun 407, 277–282. doi: 10.1016/j.bbrc.2011.02.009 21300026

[269] Raica M & Cimpean AM (2010) Platelet‐derived growth factor (PDGF)/PDGF receptors (PDGFR) Axis as target for antitumor and antiangiogenic therapy. Pharmaceuticals 3, 572–599. doi: 10.3390/ph3030572 27713269 PMC4033970

[270] Paul G , Zachrisson O , Varrone A , Almqvist P , Jerling M , Lind G , Rehncrona S , Linderoth B , Bjartmarz H , Shafer LL *et al*. (2015) Safety and tolerability of intracerebroventricular PDGF‐BB in Parkinson's disease patients. J Clin Invest 125, 1339–1346. doi: 10.1172/JCI79635 25689258 PMC4362250

[271] Padel T , Özen I , Boix J , Barbariga M , Gaceb A , Roth M & Paul G (2016) Platelet‐derived growth factor‐BB has neurorestorative effects and modulates the pericyte response in a partial 6‐hydroxydopamine lesion mouse model of Parkinson's disease. Neurobiol Dis 94, 95–105. doi: 10.1016/j.nbd.2016.06.002 27288154

[272] Lebrin F , Srun S , Raymond K , Martin S , van den Brink S , Freitas C , Bréant C , Mathivet T , Larrivée B , Thomas JL *et al*. (2010) Thalidomide stimulates vessel maturation and reduces epistaxis in individuals with hereditary hemorrhagic telangiectasia. Nat Med 16, 420–428. doi: 10.1038/nm.2131 20364125

[273] Chaudhary V , Mar F , Amador MJ , Chang A , Gibson K , Joussen AM , Kim JE , Lee J , Margaron P , Saffar I *et al*. (2025) Emerging clinical evidence of a dual role for Ang‐2 and VEGF‐A blockade with faricimab in retinal diseases. Graefes Arch Clin Exp Ophthalmol 263, 1239–1247. doi: 10.1007/s00417-024-06695-4 39708087 PMC12148975

[274] Fisher TL , Reilly CA , Winter LA , Pandina T , Jonason A , Scrivens M , Balch L , Bussler H , Torno S , Seils J *et al*. (2016) Generation and preclinical characterization of an antibody specific for SEMA4D. MAbs 8, 150–162. doi: 10.1080/19420862.2015.1102813 26431358 PMC4966508

[275] Fields GB (2019) The rebirth of matrix metalloproteinase inhibitors: moving beyond the dogma. Cells 8, 984. doi: 10.3390/cells8090984 31461880 PMC6769477

[276] Ashley RA (1999) Clinical trials of a matrix metalloproteinase inhibitor in human periodontal disease. SDD clinical research team. Ann N Y Acad Sci 30, 335–346. doi: 10.1111/j.1749-6632.1999.tb07693.x 10415739

[277] Ayoup MS , Fouad MA , Abdel‐Hamid H , Ramadan ES , Abu‐Serie MM , Noby A & Teleb M (2020) Battle tactics against MMP‐9; discovery of novel non‐hydroxamate MMP‐9 inhibitors endowed with PI3K/AKT signaling attenuation and caspase 3/7 activation via Ugi bis‐amide synthesis. Eur J Med Chem 15, 111875. doi: 10.1016/j.ejmech.2019.111875 31740054

[278] Santamaria S & de Groot R (2019) Monoclonal antibodies against metzincin targets. Br J Pharmacol 176, 52–66. doi: 10.1111/bph.14186 29488211 PMC6284333

[279] Omote Y , Deguchi K , Kono S , Liu N , Liu W , Kurata T , Yamashita T , Ikeda Y & Abe K (2014) Neurovascular protection of cilostazol in stroke‐prone spontaneous hypertensive rats associated with angiogenesis and pericyte proliferation. J Neurosci Res 92, 369–374. doi: 10.1002/jnr.23327 24375726

[280] Deguchi K , Liu N , Liu W , Omote Y , Kono S , Yunoki T , Deguchi S , Yamashita T , Ikeda Y & Abe K (2014) Pericyte protection by edaravone after tissue plasminogen activator treatment in rat cerebral ischemia. J Neurosci Res 92, 1509–1519. doi: 10.1002/jnr.23420 24938625 PMC4263311

